# Development of prescribing indicators related to opioid-related harm in patients with chronic pain in primary care—a modified e-Delphi study

**DOI:** 10.1186/s12916-023-03213-x

**Published:** 2024-01-02

**Authors:** Neetu Bansal, Stephen M. Campbell, Chiu-Yi Lin, Darren M. Ashcroft, Li-Chia Chen

**Affiliations:** 1 Drug Usage and Pharmacy Practice Group, Division of Pharmacy and Optometry, Faculty of Biology, Medicine and Health, Centre for Pharmacoepidemiology and Drug Safety, Manchester Academic Health Science Centre, School of Health Sciences, University of Manchester, Oxford Road, Manchester, M13 9PT UK; 2 Department of Public Health Pharmacy and Management, School of Pharmacy, Sefako Makgatho Health Sciences University, Molotlegi Street, Pretoria, 0208 South Africa; 3 Centre for Epidemiology and Public Health, School of Health Sciences, University of Manchester, Manchester, M13 9PL UK; 4 Division of Pharmacy and Optometry, Faculty of Biology, Medicine and Health, Manchester Academic Health Science Centre, School of Health Sciences, University of Manchester, Oxford Road, Manchester, M13 9PT UK; 5 NIHR Greater Manchester Patient Safety Research Collaboration, University of Manchester, Manchester, M13 9PL UK

**Keywords:** Primary care, Chronic pain, Prescribing quality indicators, Opioid-related harms, Preventable medication problems

## Abstract

**Background:**

Long-term opioid use is associated with dependency, addiction, and serious adverse events. Although a framework to reduce inappropriate opioid prescribing exists, there is no consensus on prescribing indicators for preventable opioid-related problems in patients with chronic pain in primary care in the UK. This study aimed to identify opioid prescription scenarios for developing indicators for prescribing opioids to patients with chronic pain in primary care.

**Methods:**

Scenarios of opioid prescribing indicators were identified from a literature review, guidelines, and government reports. Twenty-one indicators were identified and presented in various opioid scenarios concerning opioid-related harm and adverse effects, drug-drug interactions, and drug-disease interactions in certain disease conditions. After receiving ethics approval, two rounds of electronic Delphi panel technique surveys were conducted with 24 expert panellists from the UK (clinicians, pharmacists, and independent prescribers) from August 2020 to February 2021. Each indicator was rated on a 1–9 scale from inappropriate to appropriate. The score’s median, 30th and 70th percentiles, and disagreement index were calculated.

**Results:**

The panel unanimously agreed that 15 out of the 21 opioid prescribing scenarios were inappropriate, primarily due to their potential for causing harm to patients. This consensus was reflected in the low appropriateness scores (median ranging from 1 to 3). There were no scenarios with a high consensus that prescribing was appropriate. The indicators were considered inappropriate due to drug-disease interactions (*n* = 8), drug-drug interactions (*n* = 2), adverse effects (*n* = 3), and prescribed dose and duration (*n* = 2). Examples included prescribing opioids during pregnancy, concurrently with benzodiazepines, long-term without a laxative prescription and prescribing > 120-mg morphine milligram equivalent per day or long-term duration over 3 months after surgery.

**Conclusions:**

The high agreement on opioid prescribing indicators indicates that these potentially hazardous consequences are relevant and concerning to healthcare practitioners. Future research is needed to evaluate the feasibility and implementation of these indicators within primary care settings. This research will provide valuable insights and evidence to support opioid prescribing and deprescribing strategies. Moreover, the findings will be crucial in informing primary care practitioners and shaping quality outcome frameworks and other initiatives to enhance the safety and quality of care in primary care settings.

**Supplementary Information:**

The online version contains supplementary material available at 10.1186/s12916-023-03213-x.

## Background

Opioids are potent analgesics with a dependent tendency that can effectively relieve acute surgical and cancer pain [1, 2]. They are used routinely, safely and effectively for people with life-limiting illnesses and in palliative care, often for cancer [3]. The World Health Organization (WHO) included three opioids, i.e. codeine, fentanyl and morphine, in the recent Model List of Essential Medicines (the 22nd list in 2021) as they satisfied the priority healthcare needs of a population [4]; in addition, methadone was on a complimentary list for managing cancer pain [4]. Opioids are also recommended as vital medicines of the multimodal analgesic regimen recommended to manage acute or intense surgical pain [5].

However, the utilisation of prescription opioids has risen sharply over the past two decades in developed countries [6–9], notably attributable to the extensive long-term use for managing chronic pain, i.e. pain lasting more than 3 months [3]. In the United States of America (USA) and Canada, this marked increase in persistent and high-dose prescribing has been paralleled by increases in opioid-related harms, including opioid overdose death and drug misuse [10, 11], referred to as a “global pain pandemic” and “burgeoning opioid overdose epidemic” [9]. Globally, approximately 500,000 deaths are attributable to drug use, with over 70% of these deaths related to opioids and over 30% caused by overdose [12].

In the United Kingdom (UK), previous research using anonymised primary care data, i.e. the Clinical Practice Research Datalink (CPRD) from 2000 to 2021, has already found that the marked increase in the prescription of strong opioids was mainly attributable to patients without cancer [13]. Moreover, the utilisation of opioids reduced after tramadol was classified as a Schedule 3 controlled substance in June 2014 and remained stable over the past 10 years [14]. Nevertheless, the safety concerns of persistent use of opioids, such as dependency, long-term addiction and opioid-related deaths, remained, as addressed in the Public Health England 2019 report [15]. With the implementation of various policy and medicine optimisation strategies, the recently published NHS England Framework revealed that opioid prescriptions have been reduced by almost half a million in 4 years; however, while the prescribing of high-dose opioids continues to decline, the prescribing of low-dose opioids has increased [16]. This upward trend in low-dose opioid prescribing still raises concerns regarding the persistent use of opioids, as previously mentioned.

To tackle opioid misuse in the USA, numerous interventions have been implemented to exert influence over the prescribing and utilisation of opioids [17]. Notably, the 2022 Centers for Disease Control and Prevention guidelines in the USA advocate the judicious use of opioids to manage chronic pain [18]. To identify potentially hazardous prescribing, researchers in the USA have developed consensus-based indicators through collaborative efforts [19–22]. The concept of prescribing indicators was first described over two decades ago by the WHO in collaboration with the International Network for Rational Use of Drugs to formulate a list of selected indicators to investigate drug use in health facilities [23]. The development and implementation of prescribing indicators in other settings have also effectively addressed this challenge of opioid overprescribing [24–26].

In the UK, the Wessex Academic Health Science Network (AHSN) and the NHS Business Services Authority have developed a set of opioid prescribing comparators based on aggregate-level prescription data available through ePACT2 (an online application which gives authorised users access to prescription data) dashboard [27]. The dashboard allows GP practices to understand the scale of their local opioid issues and which prescribing areas are most problematic locally [27]. However, it does not identify individual patients affected by opioid prescribing within clinical prescribing systems. Although these indicators exist, they do not fully encompass individual settings’ unique characteristics and challenges and the contexts of polypharmacy [28]. Additionally, while local and national prescribing guidance and recommendations are available, there is a lack of consensus on best practices [29]. Therefore, there is a clear need to develop opioid-specific indicators explicitly tailored to individual patients’ levels for use in the primary care setting within the UK.

This study aimed to establish a comprehensive set of prescribing indicators by applying the Delphi process to gather aggregate expert opinions and facilitate achieving consensus. The Delphi process has been used successfully to identify prescribing indicators and is beneficial in situations without robust evidence [26]. This approach ensures that relevant safety concerns are considered, and the most up-to-date guidelines are incorporated into the indicator development.

## Methods

### Study design

**Fig. 1:**
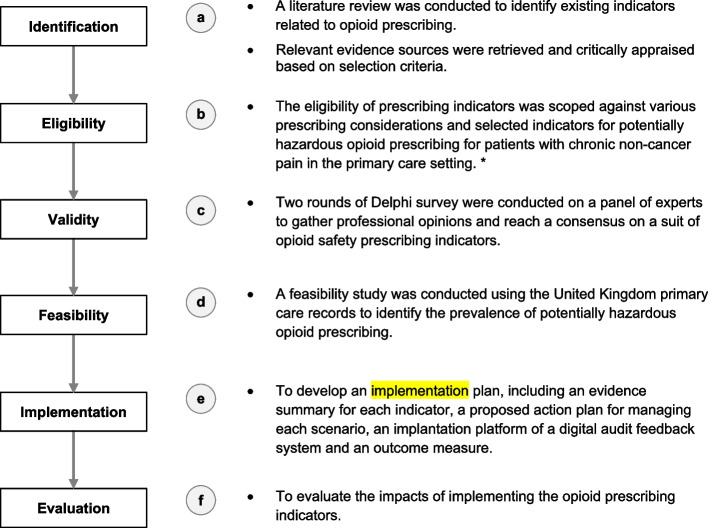
The process of developing opioid prescribing indicators. Note: This flowchart illustrates the ongoing development process, and this article presents the results for stages a, b and c. The study outcomes are summarised in Table 3, Table 4, and Additional File 8. *Semi-structured interviews with patients and healthcare professionals were conducted and identified the perceived usability of opioid prescribing indicators (Neetu Bansal, Chiu-Yi Lin, Li-Chia Chen. Patients and healthcare professionals’ perspectives on using opioids for managing chronic pain in primary care — a qualitative study in the Greater Manchester area. Poster presentation. Poster presentation. Prescribing and Research in Medicines Management (PRIMM) (UK & Ireland) conference 2022. Pharmacoepidemiology and Drug Safety, 2022. Pharmacoepidemiology and Drug Safety 2022 Vol. 31 Issue S1 Pages 14–15. DOI: https://doi.org/10.1002/pds.5499)

This study applied an electronic Delphi (e-Delphi) technique to develop a list of opioid prescribing indicators (Fig. 1a–c) [30, 31]. The Delphi technique is a structured process to obtain the written opinions or judgments of a panel of experts on a particular topic or problem. It uses a series of questionnaires or rounds where experts are asked their opinions on a particular issue and given anonymous feedback to achieve a consensus or convergence of opinions. The e-Delphi process in this study involved two rounds of online surveys conducted from August 2020 to February 2021. The first round was launched in August 2020, and the second was launched in January 2021.

### Identifying potential indicators

A review of local and national guidelines [29, 32–35] and relevant literature was conducted by two reviewers (N. B. and D. A.) by searching Google Scholar and PubMed (Additional file 1) to identify appropriate opioid prescribing indicators and guidelines available in the publicly accessible online domain (Additional file 2). This approach was chosen to provide an expedited and current overview of the available evidence, offering a more time-efficient alternative to conducting a systematic review. The research team further scoped these indicators against criteria, including the corresponding prescribing stages and the required data to identify those potential indicators that would be feasible to implement in general practices [36]. The selected indicators were further categorised by different prescribing problems (i.e. potentially inappropriate medication), such as inappropriate population, inadequate monitoring, drug-drug or drug-disease interaction, or inappropriate dose or duration [37].

### Questionnaire design

Each indicator described a scenario of prescribing opioid analgesics to typical adult patients with chronic noncancer pain in the general practice setting. The terminology, including patients, chronic noncancer pain, medical history, recent medical history, opioid analgesics, prescription of opioids (both acute and persistent prescription), and acute and persistent opioid prescription, was clearly described in the survey instructions (Additional file 3). In this study, the “*acute* prescription” refers to a prescription issued on a one-off basis for acute pain, including an “as-needed” prescription. “*Persistent* prescribing” refers to multiple prescriptions lasting 3 months or more.

For each scenario, expert panellists were asked to rate the appropriateness of the scenario using a 9-point Likert scale, where scores of 1 to 3 indicate “inappropriate” (i.e. no benefit, possible harms), 4 to 6 indicate “uncertain” (i.e. when harms and benefits are judged as approximately equal or when the best available evidence does not support judgement either way), and scores 7 to 9 indicate “appropriate” (i.e. benefits were judged to outweigh harms). This scaling method has been validated and used in published Delphi studies [38]. The rating system was clearly described in the questionnaire (Additional file 4).

Panellists were asked to provide written comments on each scenario regarding the clarity, wording or any other concerns and offer recommendations for the future development of opioid prescribing indicators. The web-based questionnaires were built on the JISC online survey platform (https://www.onlinesurveys.ac.uk) (Additional file 5, Additional file 6). The first round of the e-Delphi questionnaire was piloted with two senior hospital pharmacists to improve clarity and identify any ambiguities with the questions and the instructions. Feedback from the pilot was incorporated into the final version of the questionnaire.

### Participants and recruitment

Experts for the e-Delphi were defined as qualified healthcare professionals with experience and interest in opioid prescribing, including pharmacists, general practitioners (GPs), anaesthetists and academic researchers in the UK. The expert panel was selected from researchers’ professional and academic networks and evaluated carefully for their qualifications and expertise in pain management. Two reviewers (N. B. and L. C. C.) assessed candidates’ academic and professional backgrounds, including research publications, clinical experience and contributions to the field. Subsequently, the research team members (N. B., L. C. C., S. C., and D. M. A.) discussed and agreed on the final list.

Through introductory emails, a convenience sample [39] of potential experts with experience managing chronic pain patients was identified through researchers’ professional and social networks from various geographical areas to gather expressions of interest. Participants were invited via email and provided a participant information leaflet to ensure they were fully informed before accepting. Although the optimal size of a Delphi panel has not been defined in the literature, a target of a minimum of 20 experts was set before the study. All participants consented to this study before starting the first survey round. Participants were informed that they could withdraw anytime without reason. Each member’s identity was anonymous to other panel members and was only known to the research team.

### Data collection

In the first round of the e-Delphi survey, panellists received a hyperlink to access the online survey within 1 week after agreeing to participate and a reminder by email. They were given a 3-week window to complete the survey. A reminder was promptly sent to those who had not responded within this period to ensure a timely and thorough data collection process. The survey presented 20 scenarios and asked panellists to rate the appropriateness and comment on the clarity of each scenario for being a potential prescribing indicator. Panellists were also allowed to suggest new scenarios. All the data were downloaded onto the university’s password-protected drive for processing data analysis. According to participants’ comments in the first round of the e-Delphi survey, some scenarios were modified to enhance clarity. Similarly, in the second round of the e-Delphi survey, panellists were sent a reminder via email, and they were given a 3-week timeframe to complete the survey.

Panellists were presented with a summary of participants’ comments (in four categories) (Additional file 6) and a summary of the results from the first round, including the individual panellist’s rating, the number of panellists rating any of the nine scores, the median score of the panel, and the panel’s consensus (i.e. agreement or disagreement on the inappropriateness of the scenario) (Additional file 7). Panellists were also asked to rerate the appropriateness of original (*n* = 20) and modified (*n* = 13) scenarios and the feasibility of applying the indicators in general practice.

### Data analysis

**Fig. 2:**
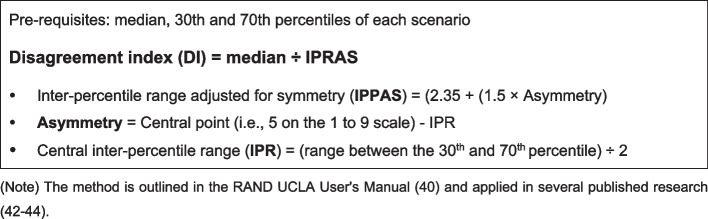
Formulas for calculating the disagreement index for each scenario. Note: The method is outlined in the RAND UCLA user’s manual [40] and applied in several published research [42–44]

After completing the first round, the median, 30th, and 70th percentiles of the score were calculated. In addition, the disagreement index (DI) was calculated by dividing the median by the inter-percentile range adjusted for symmetry (Fig. 2) [40]. If the DI is greater than 1, there is a disagreement across the participant’s responses. In contrast, if the DI is less than 1, the result reaches agreement. If *DI* < 1, the median score between 1 to 3, 4 to 6, and 7 to 9 indicated agreement on the scenario’s inappropriateness, equivocality, and appropriateness, respectively. These statistical methods were chosen because they have proven effective in measuring consensus in other Delphi studies [41] and have been used in various consensus studies [42–44]. Likewise, after completing the second survey round, the median and 30th and 70th percentiles of the score were used to calculate the DI for each indicator to judge the consensus. These results were descriptively presented in a table to compare the results in rounds 1 and 2.

In addition, the free-text comments provided by the panellists for each scenario in the first round of the survey were analysed qualitatively. The results were further categorised into four themes: concerns about the scenario, exceptions when the scenario might be appropriate, mitigation approaches for the inappropriate scenario and the feasibility of applying this scenario as an indicator in routine practice. Panellists’ comments were considered to modify or introduce new indicators in round 2.

## Results

### Potential indicators for opioid prescribing safety

A set of 32 eligible indicators was initially retrieved by the rapid review and scoped against attributes of quality indicators, such as appropriateness, content validity, and feasibility [45, 46] and narrowed down to 20 potential indicators for inappropriate opioid prescribing (Additional file 8) [47–101]. The evidence sources underpinning 20 potential indicators were retrieved and categorised based on the type of study (i.e. original study, systematic review, guidelines) to gain a clear overview of the strength and reliability of evidence (Additional file 9) [47–101].

The identified indicators described harmful opioid prescribing patterns most frequently in scenarios involving persistent opioid prescription in patients with specific morbidities (*n* = 8), including dementia, chronic obstructive pulmonary disease or asthma, hypothyroidism, myasthenia gravis, paralytic ileus, galactose intolerance, lactase deficiency or glucose-galactose malabsorption, and history of substance abuse or ventricular tachycardia (Table 1).

**Table 1:** Categorising the scenarios by types of prescribing problems

**Type of prescribing problems**^a^	**Number label of indicators**	**Number of A/IA**	
		**Round 1**	**Round 2**
Inappropriate for the population (*n* = 4)	2 (pregnancy), 15 (renal impairment), 18 (hepatic impairment), 19 (older people with a history of falls)	4	4
Opioid-disease interaction (*n* = 8)	1 (substance abuse history), 3 (hypothyroidism), 4 (paralytic ileus), 5 (dementia), 6 (chronic obstructive pulmonary disease or asthma), 8 (myasthenia gravis), 12 (galactose intolerance, lactase deficiency or glucose-galactose malabsorption), 20 (medical history of ventricular tachycardia)	4	5
Opioid-drug interaction (*n* = 4)	7 (carbamazepine, phenytoin, or phenobarbital), 9 (antidepressants), 10 (benzodiazepines), 11 (gabapentinoids)	1	2
Inappropriate dose (*n* = 1)	16 (≥ 120 mg of oral morphine equivalent dose per day)	1	1
Inappropriate duration (*n* = 1)	17 (> 3 months after surgery)	1	1
Omission (*n* = 2)	13 (patient with constipation and without a concurrently prescribed laxative), 14 (opioids ≥ 6 months without a concurrently prescribed laxative)	2	2

Note: *A/IA* agreement (disagreement index < 1) on the inappropriateness. ^a^The number (*n*) of indicators in each category is presented

Furthermore, the scenarios also included opioid prescribing in high-risk patient groups (pregnancy, renal or hepatic impairment, or older people with a history of falls; *n* = 4), co-prescription of opioids with other high-risk drug classes (antidepressants, benzodiazepines, gabapentinoids, carbamazepine, phenytoin, or phenobarbital; *n* = 4), inappropriate high dose (≥ 120 mg of oral morphine equivalent dose per day) and longer duration (> 3 months after surgery), and persistent prescription of opioids without appropriate preventative measures as co-prescribing a laxative (Table 1). No scenario was for inadequate monitoring of opioid prescribing.

### Characteristics of panellists

A total of 51 experts were invited to participate in the study, of whom 24 agreed. The first round of the e-Delphi was completed by all 24 experts who had initially agreed to participate, and the second stage was completed by 19 out of the 24 participants (Table 2). The expert panellists comprised academic pharmacists (*n* = 3), general practice pharmacists (*n* = 7), pharmacists working in clinical commissioning groups (*n* = 3), hospital pharmacists (*n* = 3), pain specialist nurses (*n* = 1), general practitioners (*n* = 5), and consultant anaesthetists (*n* = 2).

**Table 2:** Demographic details of 24 participants who took part in the e-Delphi survey

Position	Profession	Employer
Associate professor in clinical pharmacy practice and specialist pharmacist in pain management	Pharmacist	Academic institution
Advanced pharmacist practitioner; chair of the Primary Care Pharmacy Association pain group	Pharmacist	General practice
Consultant anaesthetist with an interest in pain	Doctor	NHS hospital
Pain specialist nurse	Nurse	NHS hospital
Consultant senior lecturer in primary health care	Doctor	Academic institution
Academic clinical lecturer in primary care and a GP	Doctor	Academic institution/general practice
Pharmacist prescriber	Pharmacist	General practice
Head of medicines optimisation	Pharmacist	Clinical commissioning group
Senior clinical pharmacist, team leader of neighbourhood integrated practice pharmacists in Salford	Pharmacist	General practice
GP project lead pharmacist	Pharmacist	General practice
Dean of the school of medicine	Doctor	
Senior GP pharmacist	Pharmacist	General practice
Head of medicines optimisation and prescribing	Pharmacist	Clinical commissioning group
Practice clinical pharmacist	Pharmacist	General practice
Practice clinical pharmacist	Pharmacist	General practice
Controlled drugs accountable officer	Pharmacist	Clinical commissioning group
Medicines information pharmacist	Pharmacist	NHS hospital
Professor in substance use	Pharmacist	Academic institution
Reader in pharmaceutical public health	Pharmacist	Academic institution
Consultant anaesthetist with an interest in pain	Doctor	NHS hospital
Professor of health policy and primary care	Doctor	Academic institution
Specialist pharmacy surgery	Pharmacist	NHS hospital
Lead pharmacist surgery	Pharmacist	NHS hospital
Medical director	Doctor	NHS hospital

### Consensus reached in the first round of the survey

Each scenario reached an agreement in the first round of the e-Delphi survey (*DI* < 1). Most of the scenarios (*n* = 13) were agreed to be inappropriate (median score: 1, 2, 2.5 or 2.5), except for 7 scenarios (scenarios 3, 6, 7, 8, 9, 11 and 12) that were agreed on a neutral opinion of the inappropriateness (median score: 4, 4.5 or 5) (Table 3).

**Table 3:** Consensus on the inappropriateness of prescribing scenarios rated in both rounds of the e-Delphi survey

No	Scenario	Round 1					Round 2						
		**Median** (30th, 70th)	**Number of ratings**			**Agreement** (index)	**Median** (30th, 70th)	**Number of ratings**				**Agreement** (index)	**Modified**
			1–3	4–6	7–9			1–3	4–6	7–9			
1	Persistent prescription of opioid analgesics to a patient with a medical history of alcohol addiction, abuse, or dependence [32, 47–49]	3 (2, 3)	18	5	1	A/IA (0.275)	2 (2, 3)	19	0	0		A/IA (0.220)	
2	Acute or persistent prescription of opioid analgesics to a woman during pregnancy [50–58]	2 (2, 3)	18	6	0	A/IA (0.220)	3 (2, 3)	17	2	0		A/IA (0.330)	#
3	Persistent prescription of opioid analgesics to a patient with hypothyroidism [59–61]	5 (4, 7)	7	10	7	Neutral (0.658)	5 (4, 6)	4	12	3		Neutral (0.599)	#
4	Persistent prescription of opioid analgesics to a patient with paralytic ileus [62, 63]	2 (1, 2)	23	1	0	A/IA (0.220)	2 (1, 2)	19	0	0		A/IA (0.220)	
5	Persistent prescription of opioid analgesics to a patient with dementia [64, 65]	3 (2, 4)	16	7	1	A/IA (0.299)	2 (2, 3)	15	4	0		A/IA (0.220)	
6	Persistent prescription of opioid analgesics to a patient with chronic obstructive pulmonary disease or asthma [66]	4 (3, 6)	10	10	4	Neutral (0.526)	4 (3, 4)	9	9	1		Neutral (0.440)	#
7	Co-prescription of opioid analgesics with carbamazepine, phenytoin, or phenobarbital to a patient with epilepsy [67–71]	4 (3, 6)	9	11	4	Neutral (0.502)	3 (3, 5)	10	8	1		A/IA^a^ (0.359)	#
8	Persistent prescription of opioid analgesics to a patient with myasthenia gravis [72–74]	4 (3, 5)	11	10	3	Neutral (0.458)	3 (2, 4)	10	7	2		A/IA^a^ (0.359)	
9	Acute or persistent co-prescription of opioid analgesics with antidepressants, i.e. monoamine oxidase inhibitors, selective serotonin reuptake inhibitors, or serotonin and norepinephrine [32, 68, 69, 75–78]	5 (4, 6)	7	13	4	Neutral (0.539)	4 (3, 5)	6	11	2		Neutral (0.479)	$
10	Acute or persistent co-prescription of opioid analgesics with a benzodiazepine [79–83]	4 (2, 5)	12	10	2	A/IA (0.461)	3 (2, 3)	14	4	1		A/IA (0.330)	#
11	Acute or persistent co-prescription of opioid analgesics with a gabapentinoid, i.e. gabapentin or pregabalin [84–87]	5 (3, 5)	11	10	3	Neutral (0.564)	4 (3, 4)	9	10	0	Neutral (0.440)		#
12	Acute or persistent prescription of opioid analgesics to a patient with galactose intolerance, lactase deficiency, or glucose-galactose malabsorption [88, 89]	5 (5, 6)	5	13	6	Neutral (0.549)	5 (3, 5)	6	13	0	Neutral (0.599)		#
13	Persistent prescription of opioid analgesics to a patient with constipation and without a concurrently prescribed laxative [90, 91]	2 (2, 3)	19	4	1	A/IA (0.229)	2 (2, 2)	18	1	0	A/IA (0.203)		#
14	Persistent prescription of opioid analgesics for greater than or equal to 6 months without a concurrently prescribed laxative [90, 91]	2 (2, 3)	18	5	1	A/IA (0.220)	2 (2, 2)	17	2	0	A/IA (0.203)		
15	Prescription of codeine or morphine to a patient with severe renal impairment, i.e. the most recent *eGFR* < 30 mL/min per 1.73 m^2^ [92, 93]	1 (2, 4)	13	8	3	A/IA (0.120)	2 (2, 2)	17	2	0	A/IA (0.203)		
16	Persistent prescription of one or more opioid analgesics at a dose above the equivalent of 120 mg of oral morphine per day [32, 61, 78]	2 (2, 2)	23	0	1	A/IA (0.211)	2 (2, 2)	19	0	0	A/IA (0.203)		#
17	Acute or persistent prescription of opioid analgesics to a patient for more than 3 months following the patient’s discharge from the hospital after surgery [94, 95]	2 (2, 2)	21	3	0	A/IA (0.203)	2 (2, 2)	19	0	0	A/IA (0.203)		#
18	Persistent prescription of opioid analgesics to a patient with at least moderate hepatic impairment [96–98]	2 (2, 5)	13	10	1	A/IA (0.251)	3 (2, 3)	17	2	0	A/IA (0.330)		
19	Persistent prescription of opioid analgesics to a patient aged over 65 years with a recent medical history of falling [81, 82, 99]	2 (2, 4)	16	8	0	A/IA (0.240)	2 (2, 2)	18	1	0	A/IA (0.203)		#
20	Persistent prescription of tramadol, buprenorphine, or oxycodone to a patient with a medical history of ventricular tachycardia [100, 101]	2 (2, 4)	17	6	1	A/IA (0.229)	2 (2,3)	15	4	0	A/IA (0.220)		

Note: *A/IA* agreement (disagreement index < 1) on the inappropriateness. Neutral, agreement (disagreement index < 1) on equivocality. ^a^Converged to the agreement on the inappropriateness in the second survey round. #Scenario modified or $split into two scenarios in the second survey round

Scenarios in four of the six prescribing problems (i.e. population, high dose, long duration of opioids and without co-prescribing laxatives) were fully agreed upon as inappropriate. However, only four out of eight scenarios in the opioid-disease interaction categories and one of four opioid-drug interaction categories were agreed upon as inappropriate, respectively (Table 1). Of the four opioid-disease scenarios agreed on equivocality (scenarios 3, 12, 6, 8); the median scores were higher in scenarios of prescribing opioids to patients with hypothyroidism, galactose intolerance, lactase deficiency or glucose-galactose malabsorption (median: 5) than for patients with chronic obstructive pulmonary disease or asthma and myasthenia gravis (median: 4). Coprescribing opioids with carbamazepine, phenytoin or phenobarbital to a patient with epilepsy or antidepressants and gabapentinoids (scenarios 7, 9, 11) were rated neither inappropriate nor appropriate (median score: 4 or 4.5).

### Qualitative results from the first round of the survey

Panellists also provided rich qualitative written information regarding their views on each scenario (Additional file 7), illustrating the complexity of prescribing decision-making and considerations not included in the scenarios. The lack of information on the indication of opioids, patient’s confirmed diagnosis, severity and management status of index diseases were common challenges to rate opioid-disease interaction scenarios as inappropriate (scenarios 3, 12, 6, 8). Generally, persistent prescribing of opioids at any dose was regarded as inappropriate. However, when co-prescribing opioids with other medications, panellists also required information on the indication, dose and duration for opioids and other co-prescribed medications and whether it was during a tapering (deprescribing) period (scenarios 7, 9, 11). Notably, panellists mentioned the high prevalence of co-prescribing opioids with antidepressants and gabapentinoids in primary care.

At the end of the first-round survey, panellists were asked to offer recommendations for the future development of opioid prescribing indicators. Various measures of factors influencing the safety of opioid prescribing, including indicators to measure medicine-related and patient-related risk factors, risk factors for aberrant opioid use behaviours and outcome-related indicators, were suggested (Additional file 10). Panellists suggested that future indicator development should focus on measurable clinical indicators rather than merely risk factors or contradictions. All the qualitative results were analysed and shared with panellists in the second-round survey. Considering the panellists’ comments, the wording of 11 scenarios was modified (mainly specifying acute or persistent opioid prescriptions). In addition, scenario 9 was split into two for specifying the co-prescribing antidepressant categories, i.e. monoamine oxidase inhibitors *vs.* selective serotonin reuptake inhibitors or serotonin-norepinephrine reuptake inhibitors.

### Consensus reached in the second round of the survey

In the second round of the e-Delphi survey, each of the 21 scenarios reached an agreement (*DI* < 1). Most of the scenarios (*n* = 15) were agreed to be inappropriate (median score: 2 or 3), except for five scenarios (scenarios 3, 6, 9, 11, 12) that were agreed to be equivocal on a neutral opinion of the inappropriateness (median score: 4 or 5) (Table 3). Two scenarios (scenarios 7 and 8) that converged from agreement on equivocality to inappropriateness in the second round were related to the opioid-disease (myasthenia gravis) and opioid-drug (carbamazepine, phenytoin or phenobarbital) interaction. In addition, four modified scenarios (derived from scenarios 6, 9, and 11) also converged from agreement on equivocality to inappropriateness (Table 4).

**Table 4:** Consensus on the inappropriateness of modified prescribing scenarios rated in the second round of the e-Delphi survey

No	Modified scenario	Round 2					Round 2 modified				
		**Median** (30th, 70th)	**Number of ratings**			**Agreement** (index)	**Median** (30th, 70th)	**Number of ratings**			**Agreement** (index)
			1–3	4–6	7–9			1–3	4–6	7–9	
2	Persistent prescription of opioid analgesics during pregnancy	3 (2, 3)	17	2	0	A/IA (0.330)	2 (2, 2)	18	0	1	A/IA (0.203)
3	Persistent prescription of opioid analgesics for a patient with untreated hypothyroidism	5 (4, 6)	4	12	3	Neutral (0.599)	4 (2, 4)	9	8	2	Neutral (0.479)
6	Persistent prescription of opioid analgesics to a patient with severe chronic obstructive pulmonary disease or asthma	4 (3, 4)	9	9	1	Neutral (0.440)	3 (2, 3)	14	5	0	A/IA^a^ (0.330)
7	Persistent prescription of tramadol or tapentadol with carbamazepine, phenytoin, or phenobarbital in a patient with epilepsy	3 (3, 5)	10	8	1	A/IA (0.359)	2 (2, 3)	15	4	0	A/IA (0.220)
9	Persistent prescription of tramadol, tapentadol, fentanyl, dextromethorphan, and pethidine with selective serotonin reuptake inhibitors or serotonin-norepinephrine reuptake inhibitors	4 (3, 5)	6	11	2	Neutral (0.479)	3 (2, 3)	14	4	1	A/IA^a^ (0.330)
	Prescription of tramadol, tapentadol, fentanyl, dextromethorphan, and pethidine with a monoamine oxidase inhibitor (MAOI), including the 14 days following the withdrawal of an MAOI						2 (2, 4)	13	3	3	A/IA^a^ (0.240)
10	Persistent prescription of opioid analgesics with a benzodiazepine	3 (2, 3)	14	4	1	A/IA (0.330)	2 (2, 3)	17	1	1	A/IA (0.220)
11	Persistent prescription of opioid analgesics with a gabapentinoid, i.e. gabapentin or pregabalin	4 (3, 4)	9	10	0	Neutral (0.440)	3 (2, 4)	12	7	0	A/IA^a^ (0.359)
12	Prescription of opioid analgesics to a patient with galactose intolerance, lactase deficiency, or glucose-galactose malabsorption	5 (3, 5)	6	13	0	Neutral (0.599)	5 (5, 5)	5	14	0	Neutral (0.508)
13	Persistent prescription of opioid analgesics without a concurrently prescribed laxative	2 (2, 2)	18	1	0	A/IA (0.203)	2 (2, 3)	14	5	0	A/IA (0.220)
16	Persistent prescription of one or more opioid analgesics at a total morphine equivalent load above 120 mg per day	2 (2, 2)	19	0	0	A/IA (0.203)	2 (2, 2)	19	0	0	A/IA (0.203)
17	Persistent prescription of opioid analgesics following the patient’s discharge from the hospital after surgery	2 (2, 2)	19	0	0	A/IA (0.203)	2 (2, 2)	19	0	0	A/IA (0.203)
19	Persistent prescription of opioid analgesics in a patient aged over 65 years with a medical history of falling	2 (2, 2)	18	1	0	A/IA (0.203)	2 (2, 3)	17	2	0	A/IA (0.220)

Note: *A/IA* agreement (disagreement index < 1) on the inappropriateness. Neutral, agreement (disagreement index < 1) on equivocality. ^a^The scenario converged to the agreement on the inappropriateness after modification

In contrast to the high agreement on inappropriateness, 14 of the 21 scenarios (modified version in the second round) reached an agreement (*ID* < 1), and only 4 scenarios were agreed upon as feasible (scenarios 8, 9, 14, 17, scenario 9 was for prescribing opioids with monoamine oxidase inhibitor) with a median score of 7. Two scenarios were agreed to be infeasible (scenarios 1 and 4) with a median score of 2 or 3, and the other eight scenarios (scenarios 2, 3, 5, 6, 11, 12, 18, 20) were agreed to be equivocal feasibility (Additional file 11).

## Discussion

A broad range of information sources were used to retrieve the indicators, including national and international guidelines. Following both rounds of the Delphi technique process, 15 of the 21 indicators were considered to describe harmful opioid prescribing patterns involving persistent opioid prescriptions in patients with specific comorbidities and high-risk patient groups. The 15 indicators should be subject to further testing for reliability, feasibility and implementation issues as part of improvement interventions, ideally using a validated testing protocol [102]. These indicators could serve as pivotal focal points for targeted improvement interventions.

Having a comprehensive set of prescribing indicators brings several benefits. First, these indicators can benefit the further development of the ePACT2 dashboard, enable the efficient monitoring of prescribing practices and identify areas of improvement, ultimately enhancing patient safety [23]. These indicators also support clinical decision-making by providing objective data and benchmarks for healthcare professionals to make informed prescribing choices for individual patients. Furthermore, the availability of prescribing indicators fosters collaboration and learning among healthcare professionals. By comparing their performance against these indicators, practitioners can share best practices and continuously improve their prescribing practices. Developing tailored prescribing indicators in primary care settings ultimately enhances prescribing practices’ quality, safety and appropriateness, benefiting patient care and outcomes.

These indicators help to operationalise the Wessex AHSN/NHS Business Services Authority ePACT2 dashboard by allowing the identification of individual patents affected by opioid prescribing within clinical prescribing systems [16]. Interestingly, while a high level of agreement was achieved on the inappropriateness of specific prescribing indicators, implying that clinicians are aware of the guidelines and the need to be cautious about prescribing opioids, the agreement on feasibility is not as strong. In addition to the lack of details in the scenarios to inform their judgement, this finding underscores the complexity of decision-making processes.

However, our study found the feasibility of implementing these indicators was challenged. Only four indicators were considered feasible for immediate implementation by the panel. This may be due to the nature of complex clinical judgement for caring for the target population (as mentioned in the qualitative results from the first round of the survey) and the lack of a standardised, explicit and straightforward definition of “feasibility”. Changing some highly prevalent, high-risk co-prescribing scenarios, such as prescribing opioids with gabapentinoids and antidepressants, may be challenged and considered “unfeasible”. Nevertheless, they are still vital issues that must be mitigated in clinical practice. Given appropriate implementation strategies, these scenarios can be reduced in high-risk opioid users in primary care [103].

Therefore, indicators with poor feasibility, meaning they are challenging to implement or identify potential risks, should be critically evaluated. Modifying or refining these indicators may be necessary to enhance their feasibility. This could involve simplifying measurement methods, clarifying data requirements or considering alternative approaches. Additionally, collaboration and engagement with relevant stakeholders can help to identify strategies to address feasibility challenges and ensure that indicators remain practical and effective in achieving their intended goals. Evidence has suggested an audit, and feedback system in primary care that provides practice with repeated comparable feedback could reduce opioid prescribing and prompt clinicians to consider alternatives [103]. However, additional efforts are needed, such as advocating the evidence-based summary of indicators, implementing collaborative strategies and involving key stakeholders, which are essential to influence prescribing behaviours [104].

Engaging various stakeholders and implementing collaborative strategies to include policymakers and healthcare professionals are crucial to making the indicators feasible. The NHS England Framework recently published provides a framework for action to support systems in developing plans to optimise standardised care for individuals taking medicines associated with dependence and withdrawal symptoms such as opioids. These indicators address the core objectives outlined in the framework, which include population health outcomes, addressing health inequalities in outcomes, experience and access, enhancing productivity and value for money and supporting broader social and economic development. By implementing these indicators, healthcare providers can effectively deliver care that ensures that opioids and other high-risk drugs, such as benzodiazepines and gabapentinoids, are gradually tapered off at the individual level [105].

Panellists provided rich qualitative information regarding their views on each scenario, illustrating the complexity of opioid prescribing decision-making for patients with chronic pain and considerations not included in the scenarios [106]. The lack of information on the indication of opioids, patient’s confirmed diagnosis, severity and management status of index diseases were common challenges to rate opioid-disease interaction scenarios as inappropriate. Generally, persistent prescribing of opioids is regarded as inappropriate, although it is also commonly seen in clinical practice. Panellists mentioned the high prevalence of co-prescribing opioids with antidepressants and gabapentinoids in primary care, which impeded the feasibility of applying these scenarios as indicators in clinical practice.

With all the efforts to tackle opioid use problems in the USA, a 5.1% decrease in opioid-related deaths was first observed in 2018 [107]. However, the rise in opioid prescribing and harm has been further aggravated by “an epidemic amid a pandemic” of COVID-19 [108]. COVID-19 has been associated with a higher proportion of patients prescribed opioids that can potentially invoke short-term and long-term patient harm [109].

It is also prudent to acknowledge that while there is apparent overuse and misuse of opioids in some developed countries, there is a parallel underuse of safe and effective opioids in low-income countries with a need for more equitable access to health and healthcare to address and reduce the “global pain divide”, with the poorest 50% of the world’s population having access to only 1% of the opioid medication [110]. The fundamental elements for ensuring high-quality healthcare systems comprise a workforce that is adequately trained to deliver services and ensures prompt patient access [111]. It is imperative to have well-informed and educated prescribers about the potential dangers of opioid medications, as well as the available alternatives. This education is crucial in the context of opioid stewardship. The aim is not only to reduce unnecessary opioid prescriptions, leading to cost savings, but also to lower the incidence of hospital admissions resulting from patient risks.

This is the first list of high-risk opioid prescribing indicators to be developed specifically for opioid medications in ambulatory care and general practice in the UK (Additional file 11). This study included the expert consensus panel involved in the Delphi process, which allowed inputs from different perspectives of staff involved in the use of opioids. This study uniquely focuses on the “inappropriateness” of prescribing scenarios, which differs from traditional “appropriateness” assessments. Overall, the results reached an excellent agreement; both rounds of the e-Delphi survey reached a consensus (*DI* < 1) for each scenario.

This study has some limitations. During the study, we encountered the COVID pandemic and lockdown, a period when there was unprecedented high pressure on the healthcare system. Therefore, there was a delay in receiving responses from participants. We are unsure whether the views on opioid safety and the use of opioids with other medications or in patients with certain conditions changed between the two rounds. It is also prudent to note that recruiting healthcare professionals was challenging during the COVID pandemic, and the composition of participants, such as the sample of clinicians being biased towards pharmacists, may skew the overall views on prescribing.

Moreover, as this was a developmental study, a rapid literature review was undertaken to identify potential scenarios/indicators. However, a systematic review might identify other potential opioid prescribing indicators. In addition, it should be noted that panel members were not provided with the evidence base for each indicator but were asked to rate the potential indicators based on their knowledge and experience following consensus methods. Providing an evidence base for each prescribing indicator can be challenging due to limited research availability, heterogeneity of evidence, contextual variations and time and resource constraints. Furthermore, the effectiveness of indicators can vary based on healthcare systems and patient populations. Patient and public involvement is also lacking in this study due to challenges posed by the COVID-related lockdown, which may have been valuable in mitigating potential bias in prioritisation and rating of the indicators.

Despite the limitations, there is potential to develop the identified indicators further to assist prescribing and deprescribing decisions to optimise opioids in patients with chronic pain, identify “at-risk” patients and prioritise care to prevent potential opioid-related harm and develop practice-based information technology systems to ensure the prescribing safety and quality of care in general practice.

## Conclusions

We have developed a list of opioid prescribing indicators to support healthcare practitioners in implementing appropriate opioid deprescribing strategies to identify and reduce hazardous prescribing in general practice. Each indicator reflects daily clinical care for patients with chronic pain treated in general practice. Future research is needed to test the feasibility of applying these identified indicators to identify the implementation and unintended consequences of applying the indicators.

### Supplementary Information

**Additional file 1.** Information sources for retrieving indicators related to the safety of opioid prescribing.

**Additional file 2.** Inclusion and exclusion criteria for identifying the potential opioid safety prescribing indicators.

**Additional file 3.** Key characteristics in each scenario.

**Additional file 4.** The nine-point Likert scale rating system.

**Additional file 5.** Consensus on Opioid Safety Prescribing Indicators Questionnaire –Round 1.

**Additional file 6.** Consensus on Opioid Safety Prescribing Indicators Questionnaire -Round 2.

**Additional file 7.** Summary of first-round results for one individual panellist.

**Additional file 8.** Scoping the identified potential opioid safety prescribing indicators.

**Additional file 9.** Evidence sources for the opioid safety prescribing indicators.

**Additional file 10.** Synopsis of the participants' comments on each scenario from the first round of a Delphi survey.

**Additional file 11.** Consensus on the feasibility of modified prescribing scenarios rated in the second round of the e-Delphi survey.

## Data Availability

The datasets used and analysed during the current study are available from the corresponding author upon reasonable request.

## Abbreviations

AHSN: Academic Health Science Network
CPRD: Clinical Practice Research Datalink
UK: United Kingdom
US: United States
WHO: World Health Organization
