# Supplementary material for: Development of prescribing indicators related to opioid-related harm in patients with chronic pain in primary care—a modified e-Delphi study
Source: BMC Med. 2024 Jan 2;22:5. doi: 10.1186/s12916-023-03213-x (PMC10763174; doi:10.1186/s12916-023-03213-x)
Supplement: Supplementary file 1 — Additional file 1. Information sources for retrieving indicators related to the safety of opioid prescribing. [file 12916_2023_3213_MOESM1_ESM.docx]

Additional file 1. Information sources for retrieving indicators related to the safety of opioid prescribing

| (1) Local prescribing indicators: Local hospital trusts in the UK  (2) Regional level indicators: Guidelines written by CCGs  (3) National-level opioid guidelines  (4) International guidelines from countries such as the United States of America, Canada and Brazil that conduct much research into opiates.  (5) Policies in countries such as the United States of America, Canada and Brazil that have conducted much research into opioids.  (6) SPC of opioids and adverse-drug reaction reports  (7) Guidelines from medical bodies concerned with the safe prescribing of medicines, such as the Faculty of Pain medicine  (8) Other bodies concerned with the safe use of opioids, such as Pain UK (https://painuk.org/).  (9) International bodies interested in opioid prescribing |
| --- |
