# Supplementary material for: Development of prescribing indicators related to opioid-related harm in patients with chronic pain in primary care—a modified e-Delphi study
Source: BMC Med. 2024 Jan 2;22:5. doi: 10.1186/s12916-023-03213-x (PMC10763174; doi:10.1186/s12916-023-03213-x)
Supplement: Supplementary file 2 — Additional file 2. Inclusion and exclusion criteria for identifying the potential opioid safety prescribing indicators. [file 12916_2023_3213_MOESM2_ESM.docx]

**Additional file 2.** **Inclusion and exclusion criteria for identifying the potential opioid safety prescribing indicators**

| **Inclusion Criteria** | **Exclusion Criteria** |
| --- | --- |
| - Mentions opioids currently prescribed or monitored in the UK | - Use of opioids in palliative care |
| - Mentions opioids that are prescribed in tertiary or secondary care but are monitored in primary care | - Acute prescribing of opioids |
| - Patients prescribed opioids for chronic non-cancer pain | - Use of opioids in cancer |
| - Describes a prescribing practice that could carry a potential risk or harm to a patient’s wellbeing | - Indicators attributed to prescribing error rather than prescribing quality |
| - Describes an opioid prescribing or monitoring scenario that could be modified for use in the UK GP system | - Describes a scenario that would only occur very rarely in the UK GP system |
|  | - The indicator includes an element that would not be operational through the GP electronic system |
