# Supplementary material for: Development of prescribing indicators related to opioid-related harm in patients with chronic pain in primary care—a modified e-Delphi study
Source: BMC Med. 2024 Jan 2;22:5. doi: 10.1186/s12916-023-03213-x (PMC10763174; doi:10.1186/s12916-023-03213-x)
Supplement: Supplementary file 3 — Additional file 3. Key characteristics in each scenario. [file 12916_2023_3213_MOESM3_ESM.docx]

**Additional file 3.** **Key characteristics in each scenario**

Each of the 'Opioid Safety Prescribing Indicators' describes a scenario of prescribing opioid analgesics to adult patients with chronic non-cancer pain in the general practice setting.

- The '**patients**' refer to the 'average' patients of any gender, aged over 18 years and registered with the general practice for at least six months.
- The '**chronic non-cancer pain**' refers to pain unrelated to cancer and persistent for over three months, such as low back pain, osteoarthritis, rheumatoid arthritis, neuropathic pain, fibromyalgia etc.
- Patients with acute pain or at the end-of-life stage, regardless of cancer, are not covered by the scenarios in this survey.

The following key characteristics apply to all scenarios except for some circumstances which will be specified.

- The '**medical history**' refers to any conditions documented in the patient's electronic health records.
- The '**recent medical history**' is medical conditions recorded in the patient's electronic health records in the past 12 months.
- The '**opioid analgesics**' refer to opioid preparations prescribed for pain relief. These include morphine, fentanyl, oxycodone, buprenorphine, hydromorphone, pethidine, tapentadol, tramadol, codeine, dihydrocodeine, dextropropoxyphene and meptazinol, based on the British National Formulary classification. Medication such as methadone and buprenorphine sublingual tablets which are indicated for opioid substitution therapy, is not included.
- The '**persistent**' prescribing refers to multiple prescriptions lasting three months or more.
- The '**prescription of opioids**' refers to both acute and persistent prescriptions of opioids. The acute prescription refers to a prescription issued on a one-off basis for acute pain, including an 'as needed' prescription.
