# Supplementary material for: Development of prescribing indicators related to opioid-related harm in patients with chronic pain in primary care—a modified e-Delphi study
Source: BMC Med. 2024 Jan 2;22:5. doi: 10.1186/s12916-023-03213-x (PMC10763174; doi:10.1186/s12916-023-03213-x)
Supplement: Supplementary file 4 — Additional file 4. The nine-point Likert scale rating system. [file 12916_2023_3213_MOESM4_ESM.docx]

**Additional file 4. The nine-point Likert scale rating system**

## Appropriateness rating

In this survey, the scoring system is based on a nine-point scale for the appropriateness and feasibility of each scenario.

To rate the appropriateness, score 1 indicating `extremely inappropriate` and 9 indicating `extremely appropriate’ to prescribe opioid analgesics to average adult patients with chronic noncancer pain in the general practice setting.

- Scores 1 to 3: **Inappropriate** (i.e. no benefit, possible harms).
- Scores 4 to 6: **Uncertainty** (i.e. when harms and benefits are judged as approximately equal or when the best available evidence does not support a judgement either way).
- Scores 7 to 9: **Appropriate** (i.e. benefits are judged to outweigh harms).

The vignettes of the scale are:

1. Inappropriate – no exceptions
2. Inappropriate – occasional exceptions
3. Inappropriate – some general exceptions
4. Equivocal but concerned about the average patient
5. Equivocal
6. Equivocal but probably OK in the average patient
7. Appropriate – some general exceptions
8. Appropriate – occasional exceptions
9. Appropriate – no exceptions

## Feasibility rating

To rate the feasibility, score 1 indicates `extremely unfeasible` and 9 indicates `extremely feasible’ to implement feasible to implement the indicators on average adult patients with chronic noncancer pain in the general practice setting.

You may consider various aspects of the feasibility, including data recording or reliability, human resources, availability of alternatives, and financial and other restraints.

- Scores 1 to 3: **Unfeasible** (i.e. limited resource and capacity, high risk of failure).
- Scores 4 to 6: **Uncertainty** (i.e. resource and capacity are judged approximately equal to challenges).
- Scores 7 to 9: **Feasible** (i.e. resource and capacity were judged to outweigh the risk of failure).

The vignettes of the scale are:

1. Unfeasible – no exceptions
2. Unfeasible – occasional exceptions
3. Unfeasible – some general exceptions
4. Equivocal but concerned about the average patient
5. Equivocal
6. Equivocal but probably OK in the average patient
7. Feasible – some general exceptions
8. Feasible – occasional exceptions
9. Feasible – no exceptions

Please remember to provide a 1-9 rating for each one of the scenarios. **Do not leave any spaces blank**.
