# Supplementary material for: Development of prescribing indicators related to opioid-related harm in patients with chronic pain in primary care—a modified e-Delphi study
Source: BMC Med. 2024 Jan 2;22:5. doi: 10.1186/s12916-023-03213-x (PMC10763174; doi:10.1186/s12916-023-03213-x)
Supplement: Supplementary file 5 — Additional file 5. Consensus on Opioid Safety Prescribing Indicators Questionnaire –Round 1. [file 12916_2023_3213_MOESM5_ESM.pdf]

Consensus on Opioid Safety Prescribing Indicators - Round 1

Page 1: Overview of this survey

Thank you for participating in this Delphi survey. We are a research team at the NIHR Greater Manchester Patient Safety Translational Research Centre at the University of Manchester. We seek to develop a set of Opioid Safety Prescribing Indicators (OSPIs) which can be considered in a clinical context to recognise appropriate responses for further ensuring the safety of opioid prescribing and optimising the opioid utilisation.

We have identified a set of indicators from a scoping review of the literature. The OSPIs are presented in the following 20 scenarios, and each scenario represents a separate indicator. The scenarios aim to reflect the day-to-day clinical practice and the prescribing decision-making on opioid analgesics for patients with chronic noncancer pain in the general practice setting. We would like to have your views on the OSPIs by rating the appropriateness of these scenarios relating to the safety of opioid prescribing in the general practice setting.

When scoring each scenario, please assume that you have all the presented clinical information available and rate the OSPIs according to your understanding and interpretation of the evidence in combination with your own clinical experience. In addition, we also would like to have your opinions on how to improve the indicators. Please refer to the following scoring instructions and vignettes for rating the OSPIs (Page 5).

This Delphi survey will be run twice to reach the experts' consensus. This is the first round of the survey. It is crucial that you answer all the questions, even if you are unsure. Providing an answer to each item is a prerequisite to moving on to the next question. It is also vital that you make every effort to complete all the rounds. Each round should take no more than **30 minutes** to complete.

On behalf of the research team, I would like to thank you again for your participation in this survey. Please let me know if you require any further information.

Dr Li-Chia Chen

Principal Investigator

Email: li-chia.chen@manchester.ac.uk

Page 2: Data protection

Any personal identifiable information we will collect in the course of this study will be processed in accordance with data protection laws as explained in the participant information sheet and the Privacy Notice for Research Participants (http://documents.manchester.ac.uk/display.aspx?DocID=37095).

In accordance with data protection law, The University of Manchester (UoM) is the Data Controller for this project. This means that we are responsible for making sure your personal information is kept secure, confidential and used only in the way you have been told it will be used. All researchers are trained with this in mind, and your data will be looked after in the following way:

Only the study team at UoM will have access to your personal information, but they will pseudonymise it as soon as possible. Only the research team will have access to the key that links this pseudonym to your personal information. Your consent form and contact details will be retained for five years. Your data will not be shared or transferred to any other organisation.

Page 3: Electronic consent

MANCHESTER 1824
The University of Manchester

If you are happy to participate in this survey, please consent to the following statements:

1. I confirm that I have read the attached information sheet (version 1.2, 26/03/2019) for the above study and have had the opportunity to consider the information and ask questions and had these answered satisfactorily. ✱ *Required*

- No, I do not confirm
- Yes, I confirm

2. I understand that my participation in the study is voluntary and that I am free to withdraw at any time without giving a reason and without detriment to myself. I understand that it will not be possible to remove my data from the project once it has been anonymised and forms part of the data set. I agree to take part on this basis. ✱ *Required*

- No, I do not confirm
- Yes, I confirm

3. I agree that any data collected may be published in anonymous form in academic books, reports or journals. ✱ *Required*

- No, I do not confirm
- Yes, I confirm

4. I understand that data collected during the study may be looked at by individuals from The University of Manchester or regulatory authorities, where it is relevant to my taking part in this research. I give permission for these individuals to have access to my data. ✱ *Required*

- No, I do not confirm
- Yes, I confirm

5. I agree that any anonymised data collected may be shared with collaborating researchers at The University of Manchester or at other institutions. ✱ *Required*

- No, I do not confirm
- Yes, I confirm

6. I agree that the researchers may retain my contact details in order to invite me to take part in future rounds of data collection and to provide me with a summary of findings for this study. ✱ *Required*

- No, I do not confirm
- Yes, I confirm

7. I agree to take part in this study. ✱ *Required*

- ○ No, I do not confirm
- ○ Yes, I confirm

Name of participant ✱ *Required*

Participant's current email address ✱ *Required*

Date of consent (DD/MM/YYYY) ✱ *Required*

Page 4: Rating system

The scoring system is based on a nine-point scale with 1 indicating `extremely inappropriate` and 9 indicating `extremely appropriate’ to prescribe opioid analgesics to adult patients with chronic noncancer pain in the general practice setting.

- Scores 1 to 3: **Inappropriate** (i.e. no benefit, possible harms).
- Scores 4 to 6: **Uncertainty** (i.e. when harms and benefits are judged as approximately equal, or when the best available evidence does not support a judgement either way).
- Scores 7 to 9: **Appropriate** (i.e. benefits were judged to outweigh harms).

The vignettes of the scale are:

1. Inappropriate – no exceptions
2. Inappropriate – occasional exceptions
3. Inappropriate – some general exceptions
4. Equivocal but concerns in the average patient
5. Equivocal
6. Equivocal but probably OK in the average patient
7. Appropriate – some general exceptions
8. Appropriate – occasional exceptions
9. Appropriate – no exceptions

Please remember to provide a 1-9 rating for each one of the 20 scenarios do not leave any spaces blank. Also, please provide your views on how to improve the indicators in the text box for each indicator.

Page 5: Key characteristics in each scenario

Each of the OSPI describes a scenario of prescribing opioid analgesics to adult patients with chronic noncancer pain in the general practice setting. The '**patients**' refer to the average patients of any gender, aged over 18 years and registered with the general practice for at least six months. The '**chronic noncancer pain**' refers to pain which is not related to cancer and persistent for more than three months, such as low back pain, osteoarthritis, rheumatoid arthritis, neuropathic pain, fibromyalgia etc.

The following key characteristics apply to all scenarios, except for some circumstances which will be specified.

- The '**medical history**' refers to any conditions which are documented in the patient's electronic health records.
- While the '**recent medical history**' is medical conditions recorded in the patients' electronic health records in the past 12 months.
- The '**opioid analgesics**' refer to opioid preparations prescribed for pain relief. These include morphine, fentanyl, oxycodone, buprenorphine, hydromorphone, pethidine, tapentadol, tramadol, codeine, dihydrocodeine, dextropropoxyphene and meptazinol, based on the British National Formulary classification. Medication such as methadone and buprenorphine sublingual tablets which are indicated for opioid substitution therapy is not included.
- An '**acute**' prescription refers to a prescription issued on a one-off basis for conditions that are often short-lived.
- The '**persistent**' prescribing refers to multiple prescriptions lasting three months or more.

Page 6: Scenario 1.

Please rate the appropriateness of this scenario regarding the safety of opioid prescribing for patients with chronic non-cancer pain in the general practice setting.

Persistent prescription of opioid analgesics to a patient with a medical history of alcohol addiction, abuse or dependence ✱ *Required*

⊞ More info

Please don't select more than 1 answer(s) per row.

Please select at least 1 answer(s).

	1. Inappropriate – no exceptions	2. Inappropriate – occasional exceptions	3. Inappropriate – some general exceptions	4. Equivocal but concerns in the average patient	5. Equivocal	6. Equivocal but probably OK in the average patient	7. Appropriate – some general exceptions	8. Appropriate – occasional exceptions	9. Appropriate – no exceptions
Appropriateness	☐	☐	☐	☐	☐	☐	☐	☐	☐

- The '**persistent'** prescribing refers to multiple prescriptions lasting three months or more.
- Scores 1 to 3: **Inappropriate** (i.e. no benefit, possible harms).
- Scores 4 to 6: **Uncertainty** (i.e. when harms and benefits are judged as approximately equal, or when the best available evidence does not support a judgement either way).
- Scores 7 to 9: **Appropriate** (i.e. benefits were judged to outweigh harms).

Do you have any comment on this scenario as an indicator for safe opioid prescribing? If you find this indicator is ambiguous or unclear, please suggest how the description and clarity can be improved.

Page 7: Scenario 2.

Please rate the appropriateness of this scenario regarding the safety of opioid prescribing for patients with chronic non-cancer pain in the general practice setting.

Acute or persistent prescription of opioid analgesics to a woman during pregnancy

Please don't select more than 1 answer(s) per row.

Please select at least 1 answer(s).

	1. Inappropriate – no exceptions	2. Inappropriate – occasional exceptions	3. Inappropriate – some general exceptions	4. Equivocal but concerns in the average patient	5. Equivocal	6. Equivocal but probably OK in the average patient	7. Appropriate – some general exceptions	8. Appropriate – occasional exceptions	9. Appropriate – no exceptions
Appropriateness	☐	☐	☐	☐	☐	☐	☐	☐	☐

- An **‘acute’** prescription refers to a prescription issued on a one-off basis for conditions that are often short-lived.
- The ‘**persistent’** prescribing refers to multiple prescriptions lasting three months or more.
- Scores 1 to 3: **Inappropriate** (i.e. no benefit, possible harms).
- Scores 4 to 6: **Uncertainty** (i.e. when harms and benefits are judged as approximately equal, or when the best available evidence does not support a judgement either way).
- Scores 7 to 9: **Appropriate** (i.e. benefits were judged to outweigh harms).

Do you have any comment on this scenario as an indicator for safe opioid prescribing? If you find this indicator is ambiguous or unclear, please suggest how the description and clarity can be improved.

Page 8: Scenario 3.

Please rate the appropriateness of this scenario regarding the safety of opioid prescribing for patients with chronic non-cancer pain in the general practice setting.

Persistent prescription of opioid analgesics to a patient with hypothyroidism

Please don't select more than 1 answer(s) per row.

Please select at least 1 answer(s).

	1. Inappropriate – no exceptions	2. Inappropriate – occasional exceptions	3. Inappropriate – some general exceptions	4. Equivocal but concerns in the average patient	5. Equivocal	6. Equivocal but probably OK in the average patient	7. Appropriate – some general exceptions	8. Appropriate – occasional exceptions	9. Appropriate – no exceptions
Appropriateness	☐	☐	☐	☐	☐	☐	☐	☐	☐

- The '**persistent'** prescribing refers to multiple prescriptions lasting three months or more.
- Scores 1 to 3: **Inappropriate** (i.e. no benefit, possible harms).
- Scores 4 to 6: **Uncertainty** (i.e. when harms and benefits are judged as approximately equal, or when the best available evidence does not support a judgement either way).
- Scores 7 to 9: **Appropriate** (i.e. benefits were judged to outweigh harms).

Do you have any comment on this scenario as an indicator for safe opioid prescribing? If you find this indicator is ambiguous or unclear, please suggest how the description and clarity can be improved.

Page 9: Scenario 4.

Please rate the appropriateness of this scenario regarding the safety of opioid prescribing for patients with chronic non-cancer pain in the general practice setting.

Persistent prescription of opioid analgesics to a patient with paralytic ileus

Please don't select more than 1 answer(s) per row.

Please select at least 1 answer(s).

	1. Inappropriate – no exceptions	2. Inappropriate – occasional exceptions	3. Inappropriate – some general exceptions	4. Equivocal but concerns in the average patient	5. Equivocal	6. Equivocal but probably OK in the average patient	7. Appropriate – some general exceptions	8. Appropriate – occasional exceptions	9. Appropriate – no exceptions
Appropriateness	☐	☐	☐	☐	☐	☐	☐	☐	☐

- The '***persistent***' prescribing refers to multiple prescriptions lasting three months or more.
- Scores 1 to 3: **Inappropriate** (i.e. no benefit, possible harms).
- Scores 4 to 6: **Uncertainty** (i.e. when harms and benefits are judged as approximately equal, or when the best available evidence does not support a judgement either way).
- Scores 7 to 9: **Appropriate** (i.e. benefits were judged to outweigh harms).

Do you have any comment on this scenario as an indicator for safe opioid prescribing? If you find this indicator is ambiguous or unclear, please suggest how the description and clarity can be improved.

Page 10: Scenario 5.

Please rate the appropriateness of this scenario regarding the safety of opioid prescribing for patients with chronic non-cancer pain in the general practice setting.

Persistent prescription of opioid analgesics to a patient with dementia

Please don't select more than 1 answer(s) per row.

Please select at least 1 answer(s).

	1. Inappropriate – no exceptions	2. Inappropriate – occasional exceptions	3. Inappropriate – some general exceptions	4. Equivocal but concerns in the average patient	5. Equivocal	6. Equivocal but probably OK in the average patient	7. Appropriate – some general exceptions	8. Appropriate – occasional exceptions	9. Appropriate – no exceptions
Appropriateness	☐	☐	☐	☐	☐	☐	☐	☐	☐

- The '***persistent'*** prescribing refers to multiple prescriptions lasting three months or more.
- Scores 1 to 3: **Inappropriate** (i.e. no benefit, possible harms).
- Scores 4 to 6: **Uncertainty** (i.e. when harms and benefits are judged as approximately equal, or when the best available evidence does not support a judgement either way).
- Scores 7 to 9: **Appropriate** (i.e. benefits were judged to outweigh harms).

Do you have any comment on this scenario as an indicator for safe opioid prescribing? If you find this indicator is ambiguous or unclear, please suggest how the description and clarity can be improved.

Page 11: Scenario 6.

Please rate the appropriateness of this scenario regarding the safety of opioid prescribing for patients with chronic non-cancer pain in the general practice setting.

Persistent prescription of opioid analgesics to a patient with chronic obstructive pulmonary disease or asthma

Please don't select more than 1 answer(s) per row.

Please select at least 1 answer(s).

	1. Inappropriate – no exceptions	2. Inappropriate – occasional exceptions	3. Inappropriate – some general exceptions	4. Equivocal but concerns in the average patient	5. Equivocal	6. Equivocal but probably OK in the average patient	7. Appropriate – some general exceptions	8. Appropriate – occasional exceptions	9. Appropriate – no exceptions
Appropriateness	☐	☐	☐	☐	☐	☐	☐	☐	☐

- The '**persistent'** prescribing refers to multiple prescriptions lasting three months or more.
- Scores 1 to 3: **Inappropriate** (i.e. no benefit, possible harms).
- Scores 4 to 6: **Uncertainty** (i.e. when harms and benefits are judged as approximately equal, or when the best available evidence does not support a judgement either way).
- Scores 7 to 9: **Appropriate** (i.e. benefits were judged to outweigh harms).

Do you have any comment on this scenario as an indicator for safe opioid prescribing? If you find this indicator is ambiguous or unclear, please suggest how the description and clarity can be improved.

Page 12: Scenario 7.

Please rate the appropriateness of this scenario regarding the safety of opioid prescribing for patients with chronic non-cancer pain in the general practice setting.

Co-prescription of opioid analgesics with carbamazepine, phenytoin or phenobarbital to a patient with epilepsy

Please don't select more than 1 answer(s) per row.

Please select at least 1 answer(s).

	1. Inappropriate – no exceptions	2. Inappropriate – occasional exceptions	3. Inappropriate – some general exceptions	4. Equivocal but concerns in the average patient	5. Equivocal	6. Equivocal but probably OK in the average patient	7. Appropriate – some general exceptions	8. Appropriate – occasional exceptions	9. Appropriate – no exceptions
Appropriateness	☐	☐	☐	☐	☐	☐	☐	☐	☐

- The '***persistent'*** prescribing refers to multiple prescriptions lasting three months or more.
- Scores 1 to 3: **Inappropriate** (i.e. no benefit, possible harms).
- Scores 4 to 6: **Uncertainty** (i.e. when harms and benefits are judged as approximately equal, or when the best available evidence does not support a judgement either way).
- Scores 7 to 9: **Appropriate** (i.e. benefits were judged to outweigh harms).

Do you have any comment on this scenario as an indicator for safe opioid prescribing? If you find this indicator is ambiguous or unclear, please suggest how the description and clarity can be improved.

Page 13: Scenario 8.

Please rate the appropriateness of this scenario regarding the safety of opioid prescribing for patients with chronic non-cancer pain in the general practice setting.

Persistent prescription of opioid analgesics to a patient with myasthenia gravis

Please don't select more than 1 answer(s) per row.

Please select at least 1 answer(s).

	1. Inappropriate – no exceptions	2. Inappropriate – occasional exceptions	3. Inappropriate – some general exceptions	4. Equivocal but concerns in the average patient	5. Equivocal	6. Equivocal but probably OK in the average patient	7. Appropriate – some general exceptions	8. Appropriate – occasional exceptions	9. Appropriate – no exceptions
Appropriateness	☐	☐	☐	☐	☐	☐	☐	☐	☐

- The '***persistent***' prescribing refers to multiple prescriptions lasting three months or more.
- Scores 1 to 3: **Inappropriate** (i.e. no benefit, possible harms).
- Scores 4 to 6: **Uncertainty** (i.e. when harms and benefits are judged as approximately equal, or when the best available evidence does not support a judgement either way).
- Scores 7 to 9: **Appropriate** (i.e. benefits were judged to outweigh harms).

Do you have any comment on this scenario as an indicator for safe opioid prescribing? If you find this indicator is ambiguous or unclear, please suggest how the description and clarity can be improved.

Page 14: Scenario 9.

Please rate the appropriateness of this scenario regarding the safety of opioid prescribing for patients with chronic non-cancer pain in the general practice setting.

Acute or persistent co-prescription of opioid analgesics with antidepressants, i.e. monoamine oxidase inhibitors, selective serotonin reuptake inhibitors, or serotonin and norepinephrine reuptake inhibitors

Please don't select more than 1 answer(s) per row.

Please select at least 1 answer(s).

	1. Inappropriate – no exceptions	2. Inappropriate – occasional exceptions	3. Inappropriate – some general exceptions	4. Equivocal but concerns in the average patient	5. Equivocal	6. Equivocal but probably OK in the average patient	7. Appropriate – some general exceptions	8. Appropriate – occasional exceptions	9. Appropriate – no exceptions
Appropriateness	☐	☐	☐	☐	☐	☐	☐	☐	☐

- An **‘acute’** prescription refers to a prescription issued on a one-off basis for conditions that are often short-lived.
- The ‘**persistent’** prescribing refers to multiple prescriptions lasting three months or more.
- Scores 1 to 3: **Inappropriate** (i.e. no benefit, possible harms).
- Scores 4 to 6: **Uncertainty** (i.e. when harms and benefits are judged as approximately equal, or when the best available evidence does not support a judgement either way).
- Scores 7 to 9: **Appropriate** (i.e. benefits were judged to outweigh harms).

Do you have any comment on this scenario as an indicator for safe opioid prescribing? If you find this indicator is ambiguous or unclear, please suggest how the description and clarity can be improved.

Page 15: Scenario 10.

Please rate the appropriateness of this scenario regarding the safety of opioid prescribing for patients with chronic non-cancer pain in the general practice setting.

Acute or persistent co-prescription of opioid analgesics with a benzodiazepine

Please don't select more than 1 answer(s) per row.

Please select at least 1 answer(s).

	1. Inappropriate – no exceptions	2. Inappropriate – occasional exceptions	3. Inappropriate – some general exceptions	4. Equivocal but concerns in the average patient	5. Equivocal	6. Equivocal but probably OK in the average patient	7. Appropriate – some general exceptions	8. Appropriate – occasional exceptions	9. Appropriate – no exceptions
Appropriateness	☐	☐	☐	☐	☐	☐	☐	☐	☐

- An **'acute'** prescription refers to a prescription issued on a one-off basis for conditions that are often short-lived.
- The '**persistent'** prescribing refers to multiple prescriptions lasting three months or more.
- Scores 1 to 3: **Inappropriate** (i.e. no benefit, possible harms).
- Scores 4 to 6: **Uncertainty** (i.e. when harms and benefits are judged as approximately equal, or when the best available evidence does not support a judgement either way).
- Scores 7 to 9: **Appropriate** (i.e. benefits were judged to outweigh harms).

Do you have any comment on this scenario as an indicator for safe opioid prescribing? If you find this indicator is ambiguous or unclear, please suggest how the description and clarity can be improved.

Page 16: Scenario 11.

Please rate the appropriateness of this scenario regarding the safety of opioid prescribing for patients with chronic non-cancer pain in the general practice setting.

Acute or persistent co-prescription of opioid analgesics with a gabapentinoid, i.e. gabapentin or pregabalin

Please don't select more than 1 answer(s) per row.

Please select at least 1 answer(s).

	1. Inappropriate – no exceptions	2. Inappropriate – occasional exceptions	3. Inappropriate – some general exceptions	4. Equivocal but concerns in the average patient	5. Equivocal	6. Equivocal but probably OK in the average patient	7. Appropriate – some general exceptions	8. Appropriate – occasional exceptions	9. Appropriate – no exceptions
Appropriateness	☐	☐	☐	☐	☐	☐	☐	☐	☐

- An **'acute'** prescription refers to a prescription issued on a one-off basis for conditions that are often short-lived.
- The '**persistent**' prescribing refers to multiple prescriptions lasting three months or more.
- Scores 1 to 3: **Inappropriate** (i.e. no benefit, possible harms).
- Scores 4 to 6: **Uncertainty** (i.e. when harms and benefits are judged as approximately equal, or when the best available evidence does not support a judgement either way).
- Scores 7 to 9: **Appropriate** (i.e. benefits were judged to outweigh harms).

Do you have any comment on this scenario as an indicator for safe opioid prescribing? If you find this indicator is ambiguous or unclear, please suggest how the description and clarity can be improved.

Page 17: Scenario 12.

Please rate the appropriateness of this scenario regarding the safety of opioid prescribing for patients with chronic non-cancer pain in the general practice setting.

Acute or persistent prescription of opioid analgesics to a patient with galactose intolerance, lactase deficiency or glucose-galactose malabsorption

Please don't select more than 1 answer(s) per row.

Please select at least 1 answer(s).

	1. Inappropriate – no exceptions	2. Inappropriate – occasional exceptions	3. Inappropriate – some general exceptions	4. Equivocal but concerns in the average patient	5. Equivocal	6. Equivocal but probably OK in the average patient	7. Appropriate – some general exceptions	8. Appropriate – occasional exceptions	9. Appropriate – no exceptions
Appropriateness	☐	☐	☐	☐	☐	☐	☐	☐	☐

- An **‘acute’** prescription refers to a prescription issued on a one-off basis for conditions that are often short-lived.
- The **‘persistent’** prescribing refers to multiple prescriptions lasting three months or more.
- Scores 1 to 3: **Inappropriate** (i.e. no benefit, possible harms).
- Scores 4 to 6: **Uncertainty** (i.e. when harms and benefits are judged as approximately equal, or when the best available evidence does not support a judgement either way).
- Scores 7 to 9: **Appropriate** (i.e. benefits were judged to outweigh harms).

Do you have any comment on this scenario as an indicator for safe opioid prescribing? If you find this indicator is ambiguous or unclear, please suggest how the description and clarity can be improved.

Page 18: Scenario 13.

Please rate the appropriateness of this scenario regarding the safety of opioid prescribing for patients with chronic non-cancer pain in the general practice setting.

Persistent prescription of opioid analgesics to a patient with constipation and without a concurrently prescribed laxative

Please don't select more than 1 answer(s) per row.

Please select at least 1 answer(s).

	1. Inappropriate – no exceptions	2. Inappropriate – occasional exceptions	3. Inappropriate – some general exceptions	4. Equivocal but concerns in the average patient	5. Equivocal	6. Equivocal but probably OK in the average patient	7. Appropriate – some general exceptions	8. Appropriate – occasional exceptions	9. Appropriate – no exceptions
Appropriateness	☐	☐	☐	☐	☐	☐	☐	☐	☐

- The **'persistent'** prescribing refers to multiple prescriptions lasting three months or more.
- Scores 1 to 3: **Inappropriate** (i.e. no benefit, possible harms).
- Scores 4 to 6: **Uncertainty** (i.e. when harms and benefits are judged as approximately equal, or when the best available evidence does not support a judgement either way).
- Scores 7 to 9: **Appropriate** (i.e. benefits were judged to outweigh harms).

Do you have any comment on this scenario as an indicator for safe opioid prescribing? If you find this indicator is ambiguous or unclear, please suggest how the description and clarity can be improved.

Page 19: Scenario 14.

Please rate the appropriateness of this scenario regarding the safety of opioid prescribing for patients with chronic non-cancer pain in the general practice setting.

Persistent prescription of opioid analgesics for greater than or equal to 6 months without a concurrently prescribed laxative

Please don't select more than 1 answer(s) per row.

Please select at least 1 answer(s).

	1. Inappropriate – no exceptions	2. Inappropriate – occasional exceptions	3. Inappropriate – some general exceptions	4. Equivocal but concerns in the average patient	5. Equivocal	6. Equivocal but probably OK in the average patient	7. Appropriate – some general exceptions	8. Appropriate – occasional exceptions	9. Appropriate – no exceptions
Appropriateness	☐	☐	☐	☐	☐	☐	☐	☐	☐

- The **'persistent'** prescribing refers to multiple prescriptions lasting three months or more.
- Scores 1 to 3: **Inappropriate** (i.e. no benefit, possible harms).
- Scores 4 to 6: **Uncertainty** (i.e. when harms and benefits are judged as approximately equal, or when the best available evidence does not support a judgement either way).
- Scores 7 to 9: **Appropriate** (i.e. benefits were judged to outweigh harms).

Do you have any comment on this scenario as an indicator for safe opioid prescribing? If you find this indicator is ambiguous or unclear, please suggest how the description and clarity can be improved.

Page 20: Scenario 15.

Please rate the appropriateness of this scenario regarding the safety of opioid prescribing for patients with chronic non-cancer pain in the general practice setting.

Prescription of codeine or morphine to a patient with severe renal impairment, i.e. the most recent eGFR<30 mL/min per 1.73 m^2

Please don't select more than 1 answer(s) per row.

Please select at least 1 answer(s).

	1. Inappropriate – no exceptions	2. Inappropriate – occasional exceptions	3. Inappropriate – some general exceptions	4. Equivocal but concerns in the average patient	5. Equivocal	6. Equivocal but probably OK in the average patient	7. Appropriate – some general exceptions	8. Appropriate – occasional exceptions	9. Appropriate – no exceptions
Appropriateness	☐	☐	☐	☐	☐	☐	☐	☐	☐

- Scores 1 to 3: **Inappropriate** (i.e. no benefit, possible harms).
- Scores 4 to 6: **Uncertainty** (i.e. when harms and benefits are judged as approximately equal, or when the best available evidence does not support a judgement either way).
- Scores 7 to 9: **Appropriate** (i.e. benefits were judged to outweigh harms).

Do you have any comment on this scenario as an indicator for safe opioid prescribing? If you find this indicator is ambiguous or unclear, please suggest how the description and clarity can be improved.

Page 21: Scenario 16.

Please rate the appropriateness of this scenario regarding the safety of opioid prescribing for patients with chronic non-cancer pain in the general practice setting.

Persistent prescription of one or more opioid analgesics at a dose above the equivalent of 120 mg of oral morphine per day

Please don't select more than 1 answer(s) per row.

Please select at least 1 answer(s).

	1. Inappropriate – no exceptions	2. Inappropriate – occasional exceptions	3. Inappropriate – some general exceptions	4. Equivocal but concerns in the average patient	5. Equivocal	6. Equivocal but probably OK in the average patient	7. Appropriate – some general exceptions	8. Appropriate – occasional exceptions	9. Appropriate – no exceptions
Appropriateness	☐	☐	☐	☐	☐	☐	☐	☐	☐

- Scores 1 to 3: **Inappropriate** (i.e. no benefit, possible harms).
- Scores 4 to 6: **Uncertainty** (i.e. when harms and benefits are judged as approximately equal, or when the best available evidence does not support a judgement either way).
- Scores 7 to 9: **Appropriate** (i.e. benefits were judged to outweigh harms).

Do you have any comment on this scenario as an indicator for safe opioid prescribing? If you find this indicator is ambiguous or unclear, please suggest how the description and clarity can be improved.

Page 22: Scenario 17.

Please rate the appropriateness of this scenario regarding the safety of opioid prescribing for patients with chronic non-cancer pain in the general practice setting.

Acute or persistent prescription of opioid analgesics to a patient for more than three months following the patient’s discharge from hospital after surgery

Please don't select more than 1 answer(s) per row.

Please select at least 1 answer(s).

	1. Inappropriate – no exceptions	2. Inappropriate – occasional exceptions	3. Inappropriate – some general exceptions	4. Equivocal but concerns in the average patient	5. Equivocal	6. Equivocal but probably OK in the average patient	7. Appropriate – some general exceptions	8. Appropriate – occasional exceptions	9. Appropriate – no exceptions
Appropriateness	☐	☐	☐	☐	☐	☐	☐	☐	☐

- An **‘acute’** prescription refers to a prescription issued on a one-off basis for conditions that are often short-lived.
- The **‘persistent’** prescribing refers to multiple prescriptions lasting three months or more.
- Scores 1 to 3: **Inappropriate** (i.e. no benefit, possible harms).
- Scores 4 to 6: **Uncertainty** (i.e. when harms and benefits are judged as approximately equal, or when the best available evidence does not support a judgement either way).
- Scores 7 to 9: **Appropriate** (i.e. benefits were judged to outweigh harms).

Do you have any comment on this scenario as an indicator for safe opioid prescribing? If you find this indicator is ambiguous or unclear, please suggest how the description and clarity can be improved.

Page 23: Scenario 18.

Please rate the appropriateness of this scenario regarding the safety of opioid prescribing for patients with chronic non-cancer pain in the general practice setting.

Persistent prescription of opioid analgesics to a patient with at least moderate hepatic impairment

Please don't select more than 1 answer(s) per row.

Please select at least 1 answer(s).

	1. Inappropriate – no exceptions	2. Inappropriate – occasional exceptions	3. Inappropriate – some general exceptions	4. Equivocal but concerns in the average patient	5. Equivocal	6. Equivocal but probably OK in the average patient	7. Appropriate – some general exceptions	8. Appropriate – occasional exceptions	9. Appropriate – no exceptions
Appropriateness	☐	☐	☐	☐	☐	☐	☐	☐	☐

- The **'persistent'** prescribing refers to multiple prescriptions lasting three months or more.
- Scores 1 to 3: **Inappropriate** (i.e. no benefit, possible harms).
- Scores 4 to 6: **Uncertainty** (i.e. when harms and benefits are judged as approximately equal, or when the best available evidence does not support a judgement either way).
- Scores 7 to 9: **Appropriate** (i.e. benefits were judged to outweigh harms).

Do you have any comment on this scenario as an indicator for safe opioid prescribing? If you find this indicator is ambiguous or unclear, please suggest how the description and clarity can be improved.

Page 24: Scenario 19.

Please rate the appropriateness of this scenario regarding the safety of opioid prescribing for patients with chronic non-cancer pain in the general practice setting.

Persistent prescription of opioid analgesics to a patient aged over 65 years with a recent medical history of falling

Please don't select more than 1 answer(s) per row.

Please select at least 1 answer(s).

	1. Inappropriate – no exceptions	2. Inappropriate – occasional exceptions	3. Inappropriate – some general exceptions	4. Equivocal but concerns in the average patient	5. Equivocal	6. Equivocal but probably OK in the average patient	7. Appropriate – some general exceptions	8. Appropriate – occasional exceptions	9. Appropriate – no exceptions
Appropriateness	☐	☐	☐	☐	☐	☐	☐	☐	☐

- The **'persistent'** prescribing refers to multiple prescriptions lasting three months or more.
- the **'recent medical history'** is medical conditions recorded in the patients' electronic health records in the past 12 months.
- Scores 1 to 3: **Inappropriate** (i.e. no benefit, possible harms).
- Scores 4 to 6: **Uncertainty** (i.e. when harms and benefits are judged as approximately equal, or when the best available evidence does not support a judgement either way).
- Scores 7 to 9: **Appropriate** (i.e. benefits were judged to outweigh harms).

Do you have any comment on this scenario as an indicator for safe opioid prescribing? If you find this indicator is ambiguous or unclear, please suggest how the description and clarity can be improved.

Page 25: Scenario 20.

Please rate the appropriateness of this scenario regarding the safety of opioid prescribing for patients with chronic non-cancer pain in the general practice setting.

Persistent prescription of tramadol, buprenorphine or oxycodone to a patient with a medical history of ventricular tachycardia

Please don't select more than 1 answer(s) per row.

Please select at least 1 answer(s).

	1. Inappropriate – no exceptions	2. Inappropriate – occasional exceptions	3. Inappropriate – some general exceptions	4. Equivocal but concerns in the average patient	5. Equivocal	6. Equivocal but probably OK in the average patient	7. Appropriate – some general exceptions	8. Appropriate – occasional exceptions	9. Appropriate – no exceptions
Appropriateness	☐	☐	☐	☐	☐	☐	☐	☐	☐

- The **'persistent'** prescribing refers to multiple prescriptions lasting three months or more.
- The **'medical history'** refers to any conditions which are documented in the patient's electronic health records.
- Scores 1 to 3: **Inappropriate** (i.e. no benefit, possible harms).
- Scores 4 to 6: **Uncertainty** (i.e. when harms and benefits are judged as approximately equal, or when the best available evidence does not support a judgement either way).
- Scores 7 to 9: **Appropriate** (i.e. benefits were judged to outweigh harms).

Do you have any comment on this scenario as an indicator for safe opioid prescribing? If you find this indicator is ambiguous or unclear, please suggest how the description and clarity can be improved.

Page 26: Additional Opioids Prescribing Safety Indicators

Is/are there any other potential prescribing safety indicator(s) not included in this survey that you think would be appropriate for monitoring opioids prescription in general practices?

More info

- [ ] No
- [ ] Yes

If yes, please list below.

Page 27: Final page

The University of Manchester

Thank you very much for completing this first round of the Delphi survey. You will shortly be contacted and invited to take part in a second round which will contain a summary of findings from this round. We look forward to your participation again in September 2020. If you have any question, please do not hesitate to contact the research team. Thank you.

Dr Li-Chia Chen (Email: li-chia.chen@manchester.ac.uk)
