# Supplementary material for: Development of prescribing indicators related to opioid-related harm in patients with chronic pain in primary care—a modified e-Delphi study
Source: BMC Med. 2024 Jan 2;22:5. doi: 10.1186/s12916-023-03213-x (PMC10763174; doi:10.1186/s12916-023-03213-x)
Supplement: Supplementary file 6 — Additional file 6. Consensus on Opioid Safety Prescribing Indicators Questionnaire -Round 2. [file 12916_2023_3213_MOESM6_ESM.pdf]

Consensus on Opioid Safety Prescribing Indicators - Round 2 (v1)

Page 1: Overview of this survey

Thank you for kindly participating in this Delphi survey, which is co-funded by the NIHR Greater Manchester Patient Safety Translational Research Centre.

We are conducting the second round of a Delphi survey to reach the experts' consensus of **Opioid Safety Prescribing Indicators**, which can be used to identify potentially hazardous events in primary care.

We are very grateful to receive many detailed and helpful comments on the 20 scenarios in the first round of the Delphi survey. We have summarised the rating results and provided a synopsis of all participants' comments in this second-round survey. According to participants' comments, some scenarios have been modified to enhance clarity (Page 4).

In this second-round survey, please consider the results from the previous round of the survey and rate the appropriateness of the 20 scenarios relating to the safety of opioid prescribing in the general practice setting again. We also would like you to rate the appropriateness of the modified scenarios. In this process it is important to rate original and modified scenarios to track overall panel ratings.

The scenarios aim to reflect the prescribing decision-making on opioid analgesics for patients with chronic non-cancer pain in the general practice setting. Each scenario represents a separate indicator. When scoring each scenario, please assume that you have all the presented clinical information available and rate the Opioid Safety Prescribing Indicators according to your understanding and interpretation of the evidence in combination with your own clinical experience.

In addition, please rate the feasibility to implement the indicators for an average adult patient with chronic non-cancer pain in an average general practice setting under average circumstances. Please refer to the following scoring instructions and vignettes for rating the appropriateness and feasibility of the Opioid Safety Prescribing Indicators (Page 5).

Please make every effort to complete this survey in full and leave no scales blank. Providing an answer to each item is a prerequisite to moving on to the next question. It should take no more than **45 minutes** to complete this survey. On behalf of the research team, I would like to thank you again for your participation in this survey. Please let me know if you require any further information.

Dr Li-Chia Chen

Li-Chia Chen

Principal Investigator

Email: li-chia.chen@manchester.ac.uk

Page 2: Data protection

We have received your consent to participant the two rounds of this Delphi survey. This page depicts the data protection policy.

Any personal identifiable information we will collect in the course of this study will be processed in accordance with data protection laws as explained in the participant information sheet and the Privacy Notice for Research Participants (http://documents.manchester.ac.uk/display.aspx?DocID=37095).

In accordance with data protection law, The University of Manchester (UoM) is the Data Controller for this project. This means that we are responsible for making sure your personal information is kept secure, confidential and used only in the way you have been told it will be used. All researchers are trained with this in mind, and your data will be looked after in the following way:

Only the study team at UoM will have access to your personal information, but they will pseudonymise it as soon as possible. Only the research team will have access to the key that links this pseudonym to your personal information. Your consent form and contact details will be retained for five years. Your data will not be shared or transferred to any other organisation.

Page 3: Key characteristics in each scenario

Each of the Opioid Safety Prescribing Indicator describes a scenario of prescribing opioid analgesics to adult patients with chronic non-cancer pain in the general practice setting.

- The ‘**patients**’ refer to the ‘average’ patients of any gender, aged over 18 years and registered with the general practice for at least six months.
- The ‘**chronic non-cancer pain**' refers to pain which is not related to cancer and persistent for more than three months, such as low back pain, osteoarthritis, rheumatoid arthritis, neuropathic pain, fibromyalgia etc.
- Patients who are with acute pain, or at the end of life stage regardless of cancer, are not covered by the scenarios in this survey.

The following key characteristics apply to all scenarios, except for some circumstances which will be specified.

- The ‘**medical history**’ refers to any conditions which are documented in the patient’s electronic health records.
- The ‘**recent medical history**' is medical conditions recorded in the patients’ electronic health records in the past 12 months.
- The ‘**opioid analgesics**’ refer to opioid preparations prescribed for pain relief. These include morphine, fentanyl, oxycodone, buprenorphine, hydromorphone, pethidine, tapentadol, tramadol, codeine, dihydrocodeine, dextropropoxyphene and meptazinol, based on the British National Formulary classification. Medication such as methadone and buprenorphine sublingual tablets which are indicated for opioid substitution therapy is not included.
- The ‘**persistent**’ prescribing refers to multiple prescriptions lasting three months or more.
- The ‘**prescription of opioids**’ refers to both acute and persistent prescription of opioids. The acute prescription refers to a prescription issued on a one-off basis for acute pain, including 'as needed' prescription.

Page 4: Results of the first-round survey

In each scenario, we present the results of the first-round Delphi survey to you.

Firstly, we present the number of participants rating on the 1-9 scale individually and indicate your choice (by the * sign). We also provide the median of panel's overall rating, the agreement reached on the appropriateness of each scenario. For example:

Category	Inappropriate			Equivocal			Appropriate		
**Scale**	**1**	**2**	**3**	**4**	**5**	**6**	**7**	**8**	**9**
**Number of choices**	*2*	*4*	*5**	*6*	*4*	*0*	*1*	*2*	*0*

Overall panel median: 4.0, agreement on equivocality (* indicates your rating in the first round).

We also provide a summary table for the result of overall panel and indicate your rating in the first round of Delphi survey (using the * sign). Please feel free to download this **overall summary table** (by clicking on the link).

Secondly, we present a synopsis of all participants' comments on each scenario from the first-round survey. The concise synopsis aims to reflect the opinions of all participants without any justification. For each of the 20 scenarios, we categorised the comments into four themes.

- The '**concern**' refers to the factors considered when rating the appropriateness of the scenario.
- The '**exception**' is the situation that this scenario might be appropriate. We include all the exceptions mentioned by participants, including those beyond the scope of this survey.
- The '**mitigation**' refers to any action that participants recommended on managing the situations presented in a scenario.
- The '**feasibility**' refers to participants' comments on the implementation of this indicator on patients with chronic non-cancer pain in primary care.

Page 5: Rating system

In this survey, the rating system is based on a nine-point scale for the appropriateness and feasibility of each scenario. The rating systeam is described as follows. Please feel free to download the **instruction page** (by clicking on the link).

Appropriateness rating

To rate the appropriateness, score 1 indicating 'extremely inappropriate' and 9 indicating 'extremely appropriate' to prescribe opioid analgesics to adult patients with chronic noncancer pain in the general practice setting.

- Scores 1 to 3: **Inappropriate** (i.e. no benefit, possible harms).
- Scores 4 to 6: **Uncertainty** (i.e. when harms and benefits are judged as approximately equal, or when the best available evidence does not support a judgement either way).
- Scores 7 to 9: **Appropriate** (i.e. benefits were judged to outweigh harms).

The vignettes of the scale are:

1. Inappropriate – no exceptions
2. Inappropriate – occasional exceptions
3. Inappropriate – some general exceptions
4. Equivocal but concerns in the average patient
5. Equivocal
6. Equivocal but probably OK in the average patient
7. Appropriate – some general exceptions
8. Appropriate – occasional exceptions
9. Appropriate – no exceptions

Feasibility rating

To rate the feasibility, score 1 indicating \`extremely unfeasible\` and 9 indicating \`extremely feasible’ to implement feasible to implement the indicators on average adult patients with chronic noncancer pain in the general practice setting. You may consider various aspects of feasibility, including data recording or reliability, human resource, availability of alternatives, financial and other restraints.

- Scores 1 to 3: **Unfeasible** (i.e. challenges to implement).
- Scores 4 to 6: **Uncertainty** (i.e. some problems to implement).
- Scores 7 to 9: **Feasible** (i.e. no problem to implement).

The vignettes of the scale are:

1. Unfeasible – no exceptions
2. Unfeasible – occasional exceptions
3. Unfeasible – some general exceptions
4. Equivocal but concerns in the average patient
5. Equivocal
6. Equivocal but probably OK in the average patient
7. Feasible – some general exceptions
8. Feasible – occasional exceptions
9. Feasible – no exceptions

Please remember to provide a 1-9 rating for each one of the 20 scenarios **do not leave any spaces blank**. Also, please provide your views on how to improve the indicators in the text box for each indicator.

Page 6: Scenario 1. Persistent prescription of opioid analgesics to a patient with a medical history of alcohol addiction, abuse or dependence.

Summary of Round 1 survey

Appropriateness rating

Category	Inappropriate			Equivocal			Appropriate		
**Scale**	**1**	**2**	**3**	**4**	**5**	**6**	**7**	**8**	**9**
**Result**	*3*	*9*	*6*	*5*	*0*	*0*	*0*	*1*	*0*

Overall panel median: 2.5, agreement on inappropriateness (* indicate your rating in the first round of Delphi sruvey).

Synopsis of comments

Concern

Persistent prescription (should generally be avoided); concomitant with other drugs; escalating dose due to abuse; other substance abuse; concurrent use substance other than alcohol; other risk factors (e.g. hepatic, cardiac and respiratory functions).

Exception

Patients who are at the end of life stage, whose pain and alcohol abuse are controlled, or pain is prioritised over other conditions.

Mitigation

At the three months of opioid prescribing, patients should be reviewed; if opioids are needed to be prescribed persistently, short-term prescription, more frequent monitoring, considering alternative drugs, informant patients about the risk, and accessing the alcohol addiction service.

Feasibility

Addition, abuse and dependence are not wholly recorded in the GP system. Once recorded, it is difficult to differentiate between the ongoing and the past addiction, abuse and dependence.

Appropriateness rating

Persistent prescription of opioid analgesics to a patient with a medical history of alcohol addiction, abuse or dependence. Please rate the appropriateness of this scenario regarding the safety of opioid prescribing for average patients with chronic non-cancer pain in the general practice setting. ✱ *Required*

More info

Please don't select more than 1 answer(s) per row.

Please select at least 1 answer(s).

	1. Inappropriate – no exceptions	2. Inappropriate – occasional exceptions	3. Inappropriate – some general exceptions	4. Equivocal but concerns in the average patient	5. Equivocal	6. Equivocal but probably OK in the average patient	7. Appropriate – some general exceptions	8. Appropriate – occasional exceptions	9. Appropriate – no exceptions
Appropriateness	☐	☐	☐	☐	☐	☐	☐	☐	☐

- The '**persistent'** prescribing refers to multiple prescriptions lasting three months or more.
- Scores 1 to 3: **Inappropriate** (i.e. no benefit, possible harms).
- Scores 4 to 6: **Uncertainty** (i.e. when harms and benefits are judged as approximately equal, or when the best available evidence does not support a judgement either way).
- Scores 7 to 9: **Appropriate** (i.e. benefits were judged to outweigh harms).

Feasibility rating

Persistent prescription of opioid analgesics to a patient with a medical history of alcohol addiction, abuse or dependence.

Please rate the feasibility of implementing this scenario regarding the safety of opioid prescribing for average patients with chronic non-cancer pain in the general practice setting.

⊞ More info

Please don't select more than 1 answer(s) per row.

Please select at least 1 answer(s).

	1. Unfeasible – no exceptions	2. Unfeasible – occasional exceptions	3. Unfeasible – some general exceptions	4. Equivocal but concerns in the average patient	5. Equivocal	6. Equivocal but probably OK in the average patient	7. Feasible – some general exceptions	8. Feasible – occasional exceptions	9. Feasible – no exceptions
Feasibility	☐	☐	☐	☐	☐	☐	☐	☐	☐

- Scores 1 to 3: **Unfeasible** (i.e. limited resource and capacity, high risk of failure).
- Scores 4 to 6: **Uncertainty** (i.e. resource and capacity are judged approximately equal to challenges).
- Scores 7 to 9: **Feasible** (i.e. resource and capacity were judged to outweigh the risk of failure).

Do you have any comment on this scenario as an indicator for safe opioid prescribing?

Page 7: Scenario 2. Acute or persistent prescription of opioid analgesics to a woman during pregnancy.

Summary of Round 1 survey

Appropriateness rating

Category	Inappropriate			Equivocal			Appropriate		
**Scale**	**1**	**2**	**3**	**4**	**5**	**6**	**7**	**8**	**9**
**Result**	*0*	*13*	*5*	*3*	*3*	*0*	*0*	*0*	*0*

Overall panel median: 2.0, agreement on inappropriateness (* indicate your rating in the first round of Delphi sruvey).

Synopsis of comments

Concerns

Persistent use in the late stage of pregnancy; indications for the prescription (e.g. tapper from previous analgesic use).

Exception

Acute use in the first trimester; short-term use (e.g. co-codamol 30/500 mg); tapping from prior opioid use; symphysis pubis dysfunction.

Mitigation

To conduct risk and benefit assessment, and if the prescription is needed, need to explain the risk to patients, manage expectations and agree on treatment duration. Dose escalation needs to be avoided.

Feasibility

Pregnancy Read code may stay on patients' records after they are pregnant. It is difficult to differentiate the ongoing or past pregnancy.

Appropriateness rating

Acute or persistent prescription of opioid analgesics to a woman during pregnancy.

Please rate the appropriateness of this scenario regarding the safety of opioid prescribing for average patients with chronic non-cancer pain in the general practice setting.

Please don't select more than 1 answer(s) per row.

Please select at least 1 answer(s).

	1. Inappropriate – no exceptions	2. Inappropriate – occasional exceptions	3. Inappropriate – some general exceptions	4. Equivocal but concerns in the average patient	5. Equivocal	6. Equivocal but probably OK in the average patient	7. Appropriate – some general exceptions	8. Appropriate – occasional exceptions	9. Appropriate – no exceptions
Appropriateness	☐	☐	☐	☐	☐	☐	☐	☐	☐

- An **'acute'** prescription refers to a prescription issued on a one-off basis for conditions that are often short-lived.
- The '**persistent'** prescribing refers to multiple prescriptions lasting three months or more.
- Scores 1 to 3: **Inappropriate** (i.e. no benefit, possible harms).
- Scores 4 to 6: **Uncertainty** (i.e. when harms and benefits are judged as approximately equal, or when the best available evidence does not support a judgement either way).
- Scores 7 to 9: **Appropriate** (i.e. benefits were judged to outweigh harms).

Appropriateness rating of revised scenario

Persistent prescription of opioid analgesics during pregnancy. Please rate the appropriateness of this scenario regarding the safety of opioid prescribing for average patients with chronic non-cancer pain in the general practice setting.

Please don't select more than 1 answer(s) per row.

Please select at least 1 answer(s).

	1. Inappropriate – no exceptions	2. Inappropriate – occasional exceptions	3. Inappropriate – some general exceptions	4. Equivocal but concerns in the average patient	5. Equivocal	6. Equivocal but probably OK in the average patient	7. Appropriate – some general exceptions	8. Appropriate – occasional exceptions	9. Appropriate – no exceptions
Appropriateness	☐	☐	☐	☐	☐	☐	☐	☐	☐

- An **'acute'** prescription refers to a prescription issued on a one-off basis for conditions that are often short-lived.
- The '**persistent'** prescribing refers to multiple prescriptions lasting three months or more.
- Scores 1 to 3: **Inappropriate** (i.e. no benefit, possible harms).
- Scores 4 to 6: **Uncertainty** (i.e. when harms and benefits are judged as approximately equal, or when the best available evidence does not support a judgement either way).
- Scores 7 to 9: **Appropriate** (i.e. benefits were judged to outweigh harms).

Feasibility rating of revised scenario

Persistent prescription of opioid analgesics during pregnancy. Please rate the feasibility of implementing this scenario regarding the safety of opioid prescribing for average patients with chronic non-cancer pain in the general practice setting.

⊞ More info

Please don't select more than 1 answer(s) per row.

Please select at least 1 answer(s).

	1. Unfeasible – no exceptions	2. Unfeasible – occasional exceptions	3. Unfeasible – some general exceptions	4. Equivocal but concerns in the average patient	5. Equivocal	6. Equivocal but probably OK in the average patient	7. Feasible – some general exceptions	8. Feasible – occasional exceptions	9. Feasible – no exceptions
Feasibility	☐	☐	☐	☐	☐	☐	☐	☐	☐

- Scores 1 to 3: **Unfeasible** (i.e. limited resource and capacity, high risk of failure).
- Scores 4 to 6: **Uncertainty** (i.e. resource and capacity are judged approximately equal to challenges).
- Scores 7 to 9: **Feasible** (i.e. resource and capacity were judged to outweigh the risk of failure).

Do you have any comment on this scenario as an indicator for safe opioid prescribing?

Page 8: Scenario 3. Persistent prescription of opioid analgesics to a patient with hypothyroidism.

Summary of Round 1 survey

Appropriateness rating

Category	Inappropriate			Equivocal			Appropriate		
**Scale**	**1**	**2**	**3**	**4**	**5**	**6**	**7**	**8**	**9**
**Result**	*0*	*3*	*4*	*2*	*4*	*4*	*2*	*3*	*2*

Overall panel median: 5.0, agreement on equivocality (* indicate your rating in the first round of Delphi sruvey).

Synopsis of comments

Concerns

Persistent opioids (generally inappropriate); the indication of opioids; type of opioids prescribed; untreated hypothyroidism (may include respiratory depression, low mood); opioids exacerbate hypothyroidism.

Exception

Acute prescription; well-controlled pain and thyroid function test result is OK.

Mitigation

Review all patients with persistent opioid prescriptions, and regular monitoring of relevant clinical indicators, e.g. thyroid function test, is needed.

Feasibility

No comment.

Appropriateness rating

Persistent prescription of opioid analgesics to a patient with hypothyroidism. Please rate the appropriateness of this scenario regarding the safety of opioid prescribing for average patients with chronic non-cancer pain in the general practice setting.

Please don't select more than 1 answer(s) per row.

Please select at least 1 answer(s).

	1. Inappropriate – no exceptions	2. Inappropriate – occasional exceptions	3. Inappropriate – some general exceptions	4. Equivocal but concerns in the average patient	5. Equivocal	6. Equivocal but probably OK in the average patient	7. Appropriate – some general exceptions	8. Appropriate – occasional exceptions	9. Appropriate – no exceptions
Appropriateness	☐	☐	☐	☐	☐	☐	☐	☐	☐

- The ‘**persistent’** prescribing refers to multiple prescriptions lasting three months or more.
- Scores 1 to 3: **Inappropriate** (i.e. no benefit, possible harms).
- Scores 4 to 6: **Uncertainty** (i.e. when harms and benefits are judged as approximately equal, or when the best available evidence does not support a judgement either way).
- Scores 7 to 9: **Appropriate** (i.e. benefits were judged to outweigh harms).

Appropriateness rating revised scenario

Persistent prescription of opioid analgesics for a patient with untreated hypothyroidism.

Please rate the appropriateness of this scenario regarding the safety of opioid prescribing for average patients with chronic non-cancer pain in the general practice setting.

Please don't select more than 1 answer(s) per row.

Please select at least 1 answer(s).

	1. Inappropriate – no exceptions	2. Inappropriate – occasional exceptions	3. Inappropriate – some general exceptions	4. Equivocal but concerns in the average patient	5. Equivocal	6. Equivocal but probably OK in the average patient	7. Appropriate – some general exceptions	8. Appropriate – occasional exceptions	9. Appropriate – no exceptions
Appropriateness	☐	☐	☐	☐	☐	☐	☐	☐	☐

- The ‘**persistent’** prescribing refers to multiple prescriptions lasting three months or more.
- Scores 1 to 3: **Inappropriate** (i.e. no benefit, possible harms).
- Scores 4 to 6: **Uncertainty** (i.e. when harms and benefits are judged as approximately equal, or when the best available evidence does not support a judgement either way).
- Scores 7 to 9: **Appropriate** (i.e. benefits were judged to outweigh harms).

Feasibility rating of revised scenario

Persistent prescription of opioid analgesics for a patient with untreated hypothyroidism.

Please rate the feasibility of implementing this scenario regarding the safety of opioid prescribing for average patients with chronic non-cancer pain in the general practice setting.

⊞ More info

Please don't select more than 1 answer(s) per row.

Please select at least 1 answer(s).

	1. Unfeasible – no exceptions	2. Unfeasible – occasional exceptions	3. Unfeasible – some general exceptions	4. Equivocal but concerns in the average patient	5. Equivocal	6. Equivocal but probably OK in the average patient	7. Feasible – some general exceptions	8. Feasible – occasional exceptions	9. Feasible – no exceptions
Feasibility	☐	☐	☐	☐	☐	☐	☐	☐	☐

- Scores 1 to 3: **Unfeasible** (i.e. limited resource and capacity, high risk of failure).
- Scores 4 to 6: **Uncertainty** (i.e. resource and capacity are judged approximately equal to challenges).
- Scores 7 to 9: **Feasible** (i.e. resource and capacity were judged to outweigh the risk of failure).

Do you have any comment on this scenario as an indicator for safe opioid prescribing?

Page 9: Scenario 4. Persistent prescription of opioid analgesics to a patient with paralytic ileus.

Summary of Round 1 survey

Appropriateness rating

Category	Inappropriate			Equivocal			Appropriate		
Scale	1	2	3	4	5	6	7	8	9
Result	*8*	*10*	*5*	*1*	*0*	*0*	*0*	*0*	*0*

Overall panel median: 2.0, agreement on inappropriateness (* indicate your rating in the first round of Delphi sruvey).

Synopsis of comments

Concerns

The severity of pain; causes of the paralytic ileus (e.g. opioid-induced, post-surgery or cancer); reasons for prescribing opioids; risk of exacerbating the paralytic ileus (due to opioid-induced constipation); patients would be dead (it is unlikely that paralytic ileus is untreated for three months).

Exception

Opioid epidural or non-oral preparations prescribed in a speciality or surgical setting; the end of life stage.

Mitigation

To discuss with patients at the diagnosis, at the initiation of opioids, about the long-term use of opioid or up-titration. Try to reduce the amount prescribing.

Feasibility

Paralytic ileus is normally diagnosed in a hospital setting, and generally not recoded in the GP system. Even a Read code of paralytic ileus is found, it is likely to be a past event.

Appropriateness rating

Persistent prescription of opioid analgesics to a patient with paralytic ileus.

Please rate the appropriateness of this scenario regarding the safety of opioid prescribing for average patients with chronic non-cancer pain in the general practice setting.

Please don't select more than 1 answer(s) per row.

Please select at least 1 answer(s).

	1. Inappropriate – no exceptions	2. Inappropriate – occasional exceptions	3. Inappropriate – some general exceptions	4. Equivocal but concerns in the average patient	5. Equivocal	6. Equivocal but probably OK in the average patient	7. Appropriate – some general exceptions	8. Appropriate – occasional exceptions	9. Appropriate – no exceptions

Appropriateness	☐	☐	☐	☐	☐	☐	☐	☐	☐

- The '**persistent'** prescribing refers to multiple prescriptions lasting three months or more.
- Scores 1 to 3: **Inappropriate** (i.e. no benefit, possible harms).
- Scores 4 to 6: **Uncertainty** (i.e. when harms and benefits are judged as approximately equal, or when the best available evidence does not support a judgement either way).
- Scores 7 to 9: **Appropriate** (i.e. benefits were judged to outweigh harms).

Feasibility rating

Persistent prescription of opioid analgesics to a patient with paralytic ileus.

Please rate the feasibility of implementing this scenario regarding the safety of opioid prescribing for average patients with chronic non-cancer pain in the general practice setting.

More info

Please don't select more than 1 answer(s) per row.

Please select at least 1 answer(s).

	1. Unfeasible – no exceptions	2. Unfeasible – occasional exceptions	3. Unfeasible – some general exceptions	4. Equivocal but concerns in the average patient	5. Equivocal	6. Equivocal but probably OK in the average patient	7. Feasible – some general exceptions	8. Feasible – occasional exceptions	9. Feasible – no exceptions
Feasibility	☐	☐	☐	☐	☐	☐	☐	☐	☐

- Scores 1 to 3: **Unfeasible** (i.e. limited resource and capacity, high risk of failure).
- Scores 4 to 6: **Uncertainty** (i.e. resource and capacity are judged approximately equal to challenges).
- Scores 7 to 9: **Feasible** (i.e. resource and capacity were judged to outweigh the risk of failure).

Do you have any comment on this scenario as an indicator for safe opioid prescribing?

Page 10: Scenario 5. Persistent prescription of opioid analgesics to a patient with dementia.

Summary of Round 1 survey

Appropriateness rating

Category	Inappropriate			Equivocal			Appropriate		
Scale	1	2	3	4	5	6	7	8	9
Result	*2*	*10*	*4*	*2*	*3*	*2*	*1*	*0*	*0*

Overall panel median: 2.5, agreement on inappropriateness (* indicate your rating in the first round of Delphi sruvey).

Synopsis of comments

Concerns

- Type of dementia, the manifestation of symptoms, comorbidities
- The assessment of pain, the severity of pain (pain causes distress, delirium, change of behaviours and exacerbate dementia),
- Type of opioids dose regimen, opioid-related side effects (e.g. fall, depression of central nervous system, cognitive impairment), persistent opioid use (should generally be avoided)
- Risk and benefit assessment: the risk of overdose and the side effects (e.g. falls) increased due to the memory loss and challenges of measuring pain.

Exception

Patients cannot articulate pain (on regular analgesics) or being very distress; patients need up-titrating of opioids; a low dose of opioids; the end of life stage.

Mitigation

Regular review; avoid dose escalation; gradually reduce or stop opioids; prevent accidental overdose; carers' awareness of side effect (e.g. drowsiness), storage, PRN dosing, and patient-specific caring guidance.

Feasibility

No comment.

Appropriateness rating

Persistent prescription of opioid analgesics to a patient with dementia. Please rate the appropriateness of this scenario regarding the safety of opioid prescribing for average patients with chronic non-cancer pain in the general practice setting.

Please don't select more than 1 answer(s) per row.

Please select at least 1 answer(s).

	1. Inappropriate – no exceptions	2. Inappropriate – occasional exceptions	3. Inappropriate – some general exceptions	4. Equivocal but concerns in the average patient	5. Equivocal	6. Equivocal but probably OK in the average patient	7. Appropriate – some general exceptions	8. Appropriate – occasional exceptions	9. Appropriate – no exceptions
Appropriateness	☐	☐	☐	☐	☐	☐	☐	☐	☐

- The '**persistent'** prescribing refers to multiple prescriptions lasting three months or more.
- Scores 1 to 3: **Inappropriate** (i.e. no benefit, possible harms).
- Scores 4 to 6: **Uncertainty** (i.e. when harms and benefits are judged as approximately equal, or when the best available evidence does not support a judgement either way).
- Scores 7 to 9: **Appropriate** (i.e. benefits were judged to outweigh harms).

Feasibility rating

Persistent prescription of opioid analgesics to a patient with dementia.

Please rate the feasibility of implementing this scenario regarding the safety of opioid prescribing for average patients with chronic non-cancer pain in the general practice setting.

⊞ More info

Please don't select more than 1 answer(s) per row.

Please select at least 1 answer(s).

	1. Unfeasible – no exceptions	2. Unfeasible – occasional exceptions	3. Unfeasible – some general exceptions	4. Equivocal but concerns in the average patient	5. Equivocal	6. Equivocal but probably OK in the average patient	7. Feasible – some general exceptions	8. Feasible – occasional exceptions	9. Feasible – no exceptions
Feasibility	☐	☐	☐	☐	☐	☐	☐	☐	☐

- Scores 1 to 3: **Unfeasible** (i.e. limited resource and capacity, high risk of failure).
- Scores 4 to 6: **Uncertainty** (i.e. resource and capacity are judged approximately equal to challenges).
- Scores 7 to 9: **Feasible** (i.e. resource and capacity were judged to outweigh the risk of failure).

Do you have any comment on this scenario as an indicator for safe opioid prescribing?

Page 11: Scenario 6. Persistent prescription of opioid analgesics to a patient with chronic obstructive pulmonary disease or asthma.

Summary of Round 1 survey

Appropriateness rating

Category	Inappropriate			Equivocal			Appropriate		
Scale	1	2	3	4	5	6	7	8	9
Result	*0*	*6*	*4*	*3*	*2*	*5*	*2*	*2*	*0*

Overall panel median: 4.0, agreement on equivocality (* indicate your rating in the first round of Delphi sruvey).

Synopsis of comments

Concerns

- Persistent opioids (should be avoided generally), the dose of opioids, opioid-related respiratory depression
- The severity of the respiratory diseases, Brittle asthma, whether COPD is well stable (e.g. frequency of exacerbation, oxygen supply)
- The severity of pain, whether the pain is controlled (e.g. mobility, quality of life)

Exception

Asthma; a low dose of opioids (e.g. morphine) for the dyspnoea in COPD or the end stage of COPD; the end of life stage.

Mitigation

Review complex patients with multiple comorbidities regularly; Pulse Oximetry test monitoring; reduce the dose of opioids; supply naloxone to patients or carers as a precaution.

Feasibility

No comment.

Appropriateness rating

Persistent prescription of opioid analgesics to a patient with chronic obstructive pulmonary disease or asthma.

Please rate the appropriateness of this scenario regarding the safety of opioid prescribing for average patients with chronic non-cancer pain in the general practice setting.

Please don't select more than 1 answer(s) per row.

Please select at least 1 answer(s).

	1. Inappropriate – no exceptions	2. Inappropriate – occasional exceptions	3. Inappropriate – some general exceptions	4. Equivocal but concerns in the average patient	5. Equivocal	6. Equivocal but probably OK in the average patient	7. Appropriate – some general exceptions	8. Appropriate – occasional exceptions	9. Appropriate – no exceptions

Appropriateness	☐	☐	☐	☐	☐	☐	☐	☐	☐

- The '**persistent'** prescribing refers to multiple prescriptions lasting three months or more.
- Scores 1 to 3: **Inappropriate** (i.e. no benefit, possible harms).
- Scores 4 to 6: **Uncertainty** (i.e. when harms and benefits are judged as approximately equal, or when the best available evidence does not support a judgement either way).
- Scores 7 to 9: **Appropriate** (i.e. benefits were judged to outweigh harms).

Appropriateness rating of revised scenario

Persistent prescription of opioid analgesics to a patient with severe chronic obstructive pulmonary disease or asthma.

Please rate the appropriateness of this scenario regarding the safety of opioid prescribing for average patients with chronic non-cancer pain in the general practice setting.

Please don't select more than 1 answer(s) per row.

Please select at least 1 answer(s).

	1. Inappropriate – no exceptions	2. Inappropriate – occasional exceptions	3. Inappropriate – some general exceptions	4. Equivocal but concerns in the average patient	5. Equivocal	6. Equivocal but probably OK in the average patient	7. Appropriate – some general exceptions	8. Appropriate – occasional exceptions	9. Appropriate – no exceptions
Appropriateness	☐	☐	☐	☐	☐	☐	☐	☐	☐

- The '**persistent'** prescribing refers to multiple prescriptions lasting three months or more.
- Scores 1 to 3: **Inappropriate** (i.e. no benefit, possible harms).
- Scores 4 to 6: **Uncertainty** (i.e. when harms and benefits are judged as approximately equal, or when the best available evidence does not support a judgement either way).
- Scores 7 to 9: **Appropriate** (i.e. benefits were judged to outweigh harms).

Feasibility rating of revised scenario

Persistent prescription of opioid analgesics to a patient with severe chronic obstructive pulmonary disease or asthma.

Please rate the feasibility of implementing this scenario regarding the safety of opioid prescribing for average patients with chronic non-cancer pain in the general practice setting.

More info

Please don't select more than 1 answer(s) per row.

Please select at least 1 answer(s).

	1. Unfeasible – no exceptions	2. Unfeasible – occasional exceptions	3. Unfeasible – some general exceptions	4. Equivocal but concerns in the average patient	5. Equivocal	6. Equivocal but probably OK in the average patient	7. Feasible – some general exceptions	8. Feasible – occasional exceptions	9. Feasible – no exceptions
Feasibility	☐	☐	☐	☐	☐	☐	☐	☐	☐

- Scores 1 to 3: **Unfeasible** (i.e. limited resource and capacity, high risk of failure).
- Scores 4 to 6: **Uncertainty** (i.e. resource and capacity are judged approximately equal to challenges).
- Scores 7 to 9: **Feasible** (i.e. resource and capacity were judged to outweigh the risk of failure).

Do you have any comment on this scenario as an indicator for safe opioid prescribing?

Page 12: Scenario 7. Co-prescription of opioid analgesics with carbamazepine, phenytoin or phenobarbital to a patient with epilepsy.

Summary of Round 1 survey

Appropriateness rating

Category	Inappropriate			Equivocal			Appropriate		
Scale	1	2	3	4	5	6	7	8	9
Result	*0*	*4*	*5*	*5*	*3*	*3*	*3*	*1*	*0*

Overall panel median: 4.0, agreement on equivocality (* indicate your rating in the first round of Delphi sruvey).

Synopsis of comments

Concerns

- Reason for prescribing opioids; whether the pain is controlled; whether epilepsy is stable.
- The type of opioids (tapentadol and tramadol); the dose of opioids.
- Opioid-related central nervous system effects; drug-drug interaction (e.g. additional drowsiness with phenobarbital, effects on metabolism).

Exception

Acute use; acute use at the post-discharge stage (no intention to continue); seizure caused by injury or trauma; if up-titrating is requested.

Mitigation

Cautiously monitoring; reduce the dose of opioids.

Feasibility

No comment.

Appropriateness rating

Co-prescription of opioid analgesics with carbamazepine, phenytoin or phenobarbital to a patient with epilepsy.

Please rate the appropriateness of this scenario regarding the safety of opioid prescribing for average patients with chronic non-cancer pain in the general practice setting.

Please don't select more than 1 answer(s) per row.

Please select at least 1 answer(s).

	1. Inappropriate – no exceptions	2. Inappropriate – occasional exceptions	3. Inappropriate – some general exceptions	4. Equivocal but concerns in the average patient	5. Equivocal	6. Equivocal but probably OK in the average patient	7. Appropriate – some general exceptions	8. Appropriate – occasional exceptions	9. Appropriate – no exceptions

Appropriateness	☐	☐	☐	☐	☐	☐	☐	☐	☐

- The ‘***persistent’*** prescribing refers to multiple prescriptions lasting three months or more.
- Scores 1 to 3: **Inappropriate** (i.e. no benefit, possible harms).
- Scores 4 to 6: **Uncertainty** (i.e. when harms and benefits are judged as approximately equal, or when the best available evidence does not support a judgement either way).
- Scores 7 to 9: **Appropriate** (i.e. benefits were judged to outweigh harms).

Appropriateness rating of revised scenario

Persistent prescription of tramadol or tapentadol with carbamazepine, phenytoin or phenobarbital in a patient with epilepsy.

Please rate the appropriateness of this scenario regarding the safety of opioid prescribing for average patients with chronic non-cancer pain in the general practice setting.

Please don't select more than 1 answer(s) per row.

Please select at least 1 answer(s).

	1. Inappropriate – no exceptions	2. Inappropriate – occasional exceptions	3. Inappropriate – some general exceptions	4. Equivocal but concerns in the average patient	5. Equivocal	6. Equivocal but probably OK in the average patient	7. Appropriate – some general exceptions	8. Appropriate – occasional exceptions	9. Appropriate – no exceptions
Appropriateness	☐	☐	☐	☐	☐	☐	☐	☐	☐

- The ‘***persistent’*** prescribing refers to multiple prescriptions lasting three months or more.
- Scores 1 to 3: **Inappropriate** (i.e. no benefit, possible harms).
- Scores 4 to 6: **Uncertainty** (i.e. when harms and benefits are judged as approximately equal, or when the best available evidence does not support a judgement either way).
- Scores 7 to 9: **Appropriate** (i.e. benefits were judged to outweigh harms).

Feasibility rating of revised scenario

Persistent prescription of tramadol or tapentadol with carbamazepine, phenytoin or phenobarbital in a patient with epilepsy.

Please rate the feasibility of implementing this scenario regarding the safety of opioid prescribing for average patients with chronic non-cancer pain in the general practice setting.

More info

Please don't select more than 1 answer(s) per row.

Please select at least 1 answer(s).

	1. Unfeasible – no exceptions	2. Unfeasible – occasional exceptions	3. Unfeasible – some general exceptions	4. Equivocal but concerns in the average patient	5. Equivocal	6. Equivocal but probably OK in the average patient	7. Feasible – some general exceptions	8. Feasible – occasional exceptions	9. Feasible – no exceptions
Feasibility	☐	☐	☐	☐	☐	☐	☐	☐	☐

- Scores 1 to 3: **Unfeasible** (i.e. limited resource and capacity, high risk of failure).
- Scores 4 to 6: **Uncertainty** (i.e. resource and capacity are judged approximately equal to challenges).
- Scores 7 to 9: **Feasible** (i.e. resource and capacity were judged to outweigh the risk of failure).

Do you have any comment on this scenario as an indicator for safe opioid prescribing?

Page 13: Scenario 8. Persistent prescription of opioid analgesics to a patient with myasthenia gravis.

Summary of Round 1 survey

Appropriateness rating

Category	Inappropriate			Equivocal			Appropriate		
**Scale**	**1**	**2**	**3**	**4**	**5**	**6**	**7**	**8**	**9**
**Result**	*2*	*4*	*5*	*6*	*4*	*0*	*1*	*2*	*0*

Overall panel median: 4.0, agreement on equivocality (* indicate your rating in the first round of Delphi sruvey).

Synopsis of comments

Concerns

- The severity of myasthenia gravis (e.g. affecting intercostal or ocular muscles) and whether myasthenia gravis is stable
- Persistent opioid prescription (generally considered inappropriate); increasing the risk of the central nervous system depression, drowsiness and fall.

Exception

Not a contraindication, depending on patients.

Mitigation

No comment.

Feasibility

No comment.

Appropriateness rating

Persistent prescription of opioid analgesics to a patient with myasthenia gravis.

Please rate the appropriateness of this scenario regarding the safety of opioid prescribing for average patients with chronic non-cancer pain in the general practice setting.

Please don't select more than 1 answer(s) per row.

Please select at least 1 answer(s).

	1. Inappropriate – no exceptions	2. Inappropriate – occasional exceptions	3. Inappropriate – some general exceptions	4. Equivocal but concerns in the average patient	5. Equivocal	6. Equivocal but probably OK in the average patient	7. Appropriate – some general exceptions	8. Appropriate – occasional exceptions	9. Appropriate – no exceptions
Appropriateness	☐	☐	☐	☐	☐	☐	☐	☐	☐

- The ‘**persistent’** prescribing refers to multiple prescriptions lasting three months or more.
- Scores 1 to 3: **Inappropriate** (i.e. no benefit, possible harms).
- Scores 4 to 6: **Uncertainty** (i.e. when harms and benefits are judged as approximately equal, or when the best available evidence does not support a judgement either way).
- Scores 7 to 9: **Appropriate** (i.e. benefits were judged to outweigh harms).

Feasibility rating

Persistent prescription of opioid analgesics to a patient with myasthenia gravis.

Please rate the feasibility of implementing this scenario regarding the safety of opioid prescribing for average patients with chronic non-cancer pain in the general practice setting.

More info

Please don't select more than 1 answer(s) per row.

Please select at least 1 answer(s).

	1. Unfeasible – no exceptions	2. Unfeasible – occasional exceptions	3. Unfeasible – some general exceptions	4. Equivocal but concerns in the average patient	5. Equivocal	6. Equivocal but probably OK in the average patient	7. Feasible – some general exceptions	8. Feasible – occasional exceptions	9. Feasible – no exceptions
Feasibility	☐	☐	☐	☐	☐	☐	☐	☐	☐

- Scores 1 to 3: **Unfeasible** (i.e. limited resource and capacity, high risk of failure).
- Scores 4 to 6: **Uncertainty** (i.e. resource and capacity are judged approximately equal to challenges).
- Scores 7 to 9: **Feasible** (i.e. resource and capacity were judged to outweigh the risk of failure).

Do you have any comment on this scenario as an indicator for safe opioid prescribing?

Page 14: Scenario 9. Acute or persistent co-prescription of opioid analgesics with antidepressants, i.e. monoamine oxidase inhibitors, selective serotonin reuptake inhibitors, or serotonin and norepinephrine reuptake inhibitors.

Summary of Round 1 survey

Appropriateness rating

Category	Inappropriate			Equivocal			Appropriate		
Scale	1	2	3	4	5	6	7	8	9
Result	*0*	*4*	*3*	*5*	*5*	*3*	*1*	*3*	*0*

Overall panel median: 4.0, agreement on equivocality (* indicate your rating in the first round of Delphi sruvey).

Synopsis of comments

Concerns

- Reasons for prescribing; comorbidities; whether patients' conditions are stable.
- Persistent opioid prescription (generally inappropriate); type of opioids (tramadol related serotonin syndrome); never use modified-release opioids; type of antidepressants (never use MAOIs as it causes severe adverse effects.
- Risk of (accidental or intentional) overdose, introducing a potential misused agent (opioids) to depressive patients.
- Drug-drug interaction (tramadol); increase sedation effects.

Exception

Acute use, e.g. for surgery or trauma; up-titrating.

Mitigation

Provide holistic approaches of pain management; ensure patients understand the expectations of treatments; review patients regularly, monitor usage and check interaction; reduce dose to avoid central nervous system depression.

Feasibility

This indicator will identify too many patients.

Appropriateness rating

Acute or persistent co-prescription of opioid analgesics with antidepressants, i.e. monoamine oxidase inhibitors, selective serotonin reuptake inhibitors, or serotonin and norepinephrine reuptake inhibitors. Please rate the appropriateness of this scenario regarding the safety of opioid prescribing for average patients with chronic non-cancer pain in the general practice setting.

Please don't select more than 1 answer(s) per row.

Please select at least 1 answer(s).

	1. Inappropriate – no exceptions	2. Inappropriate – occasional exceptions	3. Inappropriate – some general exceptions	4. Equivocal but concerns in the average patient	5. Equivocal	6. Equivocal but probably OK in the average patient	7. Appropriate – some general exceptions	8. Appropriate – occasional exceptions	9. Appropriate – no exceptions
Appropriateness	☐	☐	☐	☐	☐	☐	☐	☐	☐

- An **'acute'** prescription refers to a prescription issued on a one-off basis for conditions that are often short-lived.
- The '**persistent'** prescribing refers to multiple prescriptions lasting three months or more.
- Scores 1 to 3: **Inappropriate** (i.e. no benefit, possible harms).
- Scores 4 to 6: **Uncertainty** (i.e. when harms and benefits are judged as approximately equal, or when the best available evidence does not support a judgement either way).
- Scores 7 to 9: **Appropriate** (i.e. benefits were judged to outweigh harms).

Appropriateness rating of revised scenario (a)

Persistent prescription of tramadol, tapentadol, fentanyl, dextromethorphan, pethidine with selective serotonin reuptake inhibitors or serotonin norepinephrine reuptake inhibitors. Please rate the appropriateness of this scenario regarding the safety of opioid prescribing for average patients with chronic non-cancer pain in the general practice setting.

Please don't select more than 1 answer(s) per row.

Please select at least 1 answer(s).

	1. Inappropriate – no exceptions	2. Inappropriate – occasional exceptions	3. Inappropriate – some general exceptions	4. Equivocal but concerns in the average patient	5. Equivocal	6. Equivocal but probably OK in the average patient	7. Appropriate – some general exceptions	8. Appropriate – occasional exceptions	9. Appropriate – no exceptions
Appropriateness	☐	☐	☐	☐	☐	☐	☐	☐	☐

- An **'acute'** prescription refers to a prescription issued on a one-off basis for conditions that are often short-lived.
- The '**persistent'** prescribing refers to multiple prescriptions lasting three months or more.
- Scores 1 to 3: **Inappropriate** (i.e. no benefit, possible harms).
- Scores 4 to 6: **Uncertainty** (i.e. when harms and benefits are judged as approximately equal, or when the best available evidence does not support a judgement either way).
- Scores 7 to 9: **Appropriate** (i.e. benefits were judged to outweigh harms).

Feasibility rating of revised scenario (a)

Persistent prescription of tramadol, tapentadol, fentanyl, dextromethorphan, pethidine with selective serotonin reuptake inhibitors or serotonin norepinephrine reuptake inhibitors. Please rate the feasibility of implementing this scenario regarding the safety of opioid prescribing for average patients with chronic non-cancer pain in the general practice setting.

More info

Please don't select more than 1 answer(s) per row.

Please select at least 1 answer(s).

	1. Unfeasible – no exceptions	2. Unfeasible – occasional exceptions	3. Unfeasible – some general exceptions	4. Equivocal but concerns in the average patient	5. Equivocal	6. Equivocal but probably OK in the average patient	7. Feasible – some general exceptions	8. Feasible – occasional exceptions	9. Feasible – no exceptions
Feasibility	☐	☐	☐	☐	☐	☐	☐	☐	☐

- Scores 1 to 3: **Unfeasible** (i.e. limited resource and capacity, high risk of failure).
- Scores 4 to 6: **Uncertainty** (i.e. resource and capacity are judged approximately equal to challenges).
- Scores 7 to 9: **Feasible** (i.e. resource and capacity were judged to outweigh the risk of failure).

Appropriateness rating of revised scenario (b)

Prescription of tramadol, tapentadol, fentanyl, dextromethorphan, pethidine with a monoamine oxidase inhibitor (MAOI), including the 14-day period following the withdrawal of an MAOI.

Please rate the appropriateness of this scenario regarding the safety of opioid prescribing for average patients with chronic non-cancer pain in the general practice setting.

Please don't select more than 1 answer(s) per row.

Please select at least 1 answer(s).

	1. Inappropriate – no exceptions	2. Inappropriate – occasional exceptions	3. Inappropriate – some general exceptions	4. Equivocal but concerns in the average patient	5. Equivocal	6. Equivocal but probably OK in the average patient	7. Appropriate – some general exceptions	8. Appropriate – occasional exceptions	9. Appropriate – no exceptions
Appropriateness	☐	☐	☐	☐	☐	☐	☐	☐	☐

- An **'acute'** prescription refers to a prescription issued on a one-off basis for conditions that are often short-lived.
- The '**persistent'** prescribing refers to multiple prescriptions lasting three months or more.
- Scores 1 to 3: **Inappropriate** (i.e. no benefit, possible harms).
- Scores 4 to 6: **Uncertainty** (i.e. when harms and benefits are judged as approximately equal, or when the best available evidence does not support a judgement either way).
- Scores 7 to 9: **Appropriate** (i.e. benefits were judged to outweigh harms).

Feasibility rating of revised scenario (b)

Prescription of tramadol, tapentadol, fentanyl, dextromethorphan, pethidine with a monoamine oxidase inhibitor (MAOI), including the 14-day period following the withdrawal of an MAOI.

Please rate the feasibility of implementing this scenario regarding the safety of opioid prescribing for average patients with chronic non-cancer pain in the general practice setting.

⊞ More info

Please don't select more than 1 answer(s) per row.

Please select at least 1 answer(s).

	1. Unfeasible – no exceptions	2. Unfeasible – occasional exceptions	3. Unfeasible – some general exceptions	4. Equivocal but concerns in the average patient	5. Equivocal	6. Equivocal but probably OK in the average patient	7. Feasible – some general exceptions	8. Feasible – occasional exceptions	9. Feasible – no exceptions
Feasibility	☐	☐	☐	☐	☐	☐	☐	☐	☐

- Scores 1 to 3: **Unfeasible** (i.e. limited resource and capacity, high risk of failure).
- Scores 4 to 6: **Uncertainty** (i.e. resource and capacity are judged approximately equal to challenges).
- Scores 7 to 9: **Feasible** (i.e. resource and capacity were judged to outweigh the risk of failure).

Do you have any comment on this scenario as an indicator for safe opioid prescribing?

Page 15: Scenario 10. Acute or persistent co-prescription of opioid analgesics with a benzodiazepine.

Summary of Round 1 survey

Appropriateness rating

Category	Inappropriate			Equivocal			Appropriate		
Scale	1	2	3	4	5	6	7	8	9
Result	*0*	*8*	*4*	*4*	*4*	*2*	*1*	*1*	*0*

Overall panel median: 3.5, agreement on inappropriateness (* indicate your rating in the first round of Delphi sruvey).

Synopsis of comments

Concerns

- Persistent opioid use is generally inappropriate' but this combination is common in the elderly.
- Increased risk of overdose and side effects, e.g. central nervous system depression (e.g. sedation, drowsiness), respiratory depression (e.g. opioid-induced ventilatory impairment), falls.
- Balancing risk and benefit for the individual: considering the indication and dose of opioids and benzodiazepine, the muscle relaxant effects of benzodiazepines, and patients' other comorbidities (e.g. chronic obstructive pulmonary disease; myasthenia gravis) or substance use (e.g. alcohol).

Exception

Acute use; acute pain; post-surgical pain; end of life; low dose of benzodiazepine; up-titrating.

Mitigation

Ensure patients' awareness of the risk; supply naloxone to patients and carers and educate them about how to use it.

Feasibility

This is a common scenario, and the indicator will identify too many patients.

Appropriateness rating

Acute or persistent co-prescription of opioid analgesics with a benzodiazepine.

Please rate the appropriateness of this scenario regarding the safety of opioid prescribing for average patients with chronic non-cancer pain in the general practice setting.

Please don't select more than 1 answer(s) per row.

Please select at least 1 answer(s).

	1. Inappropriate – no exceptions	2. Inappropriate – occasional exceptions	3. Inappropriate – some general exceptions	4. Equivocal but concerns in the average patient	5. Equivocal	6. Equivocal but probably OK in the average patient	7. Appropriate – some general exceptions	8. Appropriate – occasional exceptions	9. Appropriate – no exceptions
Appropriateness	☐	☐	☐	☐	☐	☐	☐	☐	☐

- An **'acute'** prescription refers to a prescription issued on a one-off basis for conditions that are often short-lived.
- The '**persistent'** prescribing refers to multiple prescriptions lasting three months or more.
- Scores 1 to 3: **Inappropriate** (i.e. no benefit, possible harms).
- Scores 4 to 6: **Uncertainty** (i.e. when harms and benefits are judged as approximately equal, or when the best available evidence does not support a judgement either way).
- Scores 7 to 9: **Appropriate** (i.e. benefits were judged to outweigh harms).

Appropriateness rating of revised scenario

Persistent prescription of opioid analgesics with a benzodiazepine.

Please rate the appropriateness of this scenario regarding the safety of opioid prescribing for average patients with chronic non-cancer pain in the general practice setting.

Please don't select more than 1 answer(s) per row.

Please select at least 1 answer(s).

	1. Inappropriate – no exceptions	2. Inappropriate – occasional exceptions	3. Inappropriate – some general exceptions	4. Equivocal but concerns in the average patient	5. Equivocal	6. Equivocal but probably OK in the average patient	7. Appropriate – some general exceptions	8. Appropriate – occasional exceptions	9. Appropriate – no exceptions
Appropriateness	☐	☐	☐	☐	☐	☐	☐	☐	☐

- An **'acute'** prescription refers to a prescription issued on a one-off basis for conditions that are often short-lived.
- The '**persistent'** prescribing refers to multiple prescriptions lasting three months or more.
- Scores 1 to 3: **Inappropriate** (i.e. no benefit, possible harms).
- Scores 4 to 6: **Uncertainty** (i.e. when harms and benefits are judged as approximately equal, or when the best available evidence does not support a judgement either way).
- Scores 7 to 9: **Appropriate** (i.e. benefits were judged to outweigh harms).

Feasibility rating of revised scenario

Persistent prescription of opioid analgesics with a benzodiazepine.

Please rate the feasibility of implementing this scenario regarding the safety of opioid prescribing for average patients with chronic non-cancer pain in the general practice setting.

More info

Please don't select more than 1 answer(s) per row.

Please select at least 1 answer(s).

	1. Unfeasible – no exceptions	2. Unfeasible – occasional exceptions	3. Unfeasible – some general exceptions	4. Equivocal but concerns in the average patient	5. Equivocal	6. Equivocal but probably OK in the average patient	7. Feasible – some general exceptions	8. Feasible – occasional exceptions	9. Feasible – no exceptions
Feasibility	☐	☐	☐	☐	☐	☐	☐	☐	☐

- Scores 1 to 3: **Unfeasible** (i.e. limited resource and capacity, high risk of failure).
- Scores 4 to 6: **Uncertainty** (i.e. resource and capacity are judged approximately equal to challenges).
- Scores 7 to 9: **Feasible** (i.e. resource and capacity were judged to outweigh the risk of failure).

Do you have any comment on this scenario as an indicator for safe opioid prescribing?

Page 16: Scenario 11. Acute or persistent co-prescription of opioid analgesics with a gabapentinoid, i.e. gabapentin or pregabalin.

Summary of Round 1 survey

Appropriateness rating

Category	Inappropriate			Equivocal			Appropriate		
**Scale**	**1**	**2**	**3**	**4**	**5**	**6**	**7**	**8**	**9**
**Result**	*0*	*7*	*4*	*1*	*6*	*3*	*2*	*1*	*0*

Overall panel median: 4.5, agreement on equivocality (* indicate your rating in the first round of Delphi sruvey).

Synopsis of comments

Concerns

- Persistent opioid use (should be avoided).
- Nature of pain (neuropathic pain), indications for prescribing both opioids and gabapentinoids.
- Increase the risk of central nervous system depression, confusion, ataxia, falls, opioid-induced ventilation impairment.
- Dose and duration of opioids and gabapentinoids; dose escalation (increases sedative effect).

Exception

Acute use (e.g. injury or pain exacerbation); patients suffering from different types of pain; cross-tapering (gabapentinoids for neuropathic pain plus low-dose or PRN opioids).

Mitigation

Ensure the nature of pain is identified; review patients at the three months of opioid use; involve the Pain Management Team; avoid co-prescribing opioids and gabapentinoids; advise naloxone supply and education.

Feasibility

This is a common scenario, and the indicator will identify too many patients. Gabapentinoids are often not initiated with opioids at the same time; Co-codamol is commonly prescribed before gabapentinoids.

Appropriateness rating

Acute or persistent co-prescription of opioid analgesics with a gabapentinoid, i.e. gabapentin or pregabalin.

Please rate the appropriateness of this scenario regarding the safety of opioid prescribing for average patients with chronic non-cancer pain in the general practice setting.

Please don't select more than 1 answer(s) per row.

Please select at least 1 answer(s).

	1. Inappropriate – no exceptions	2. Inappropriate – occasional exceptions	3. Inappropriate – some general exceptions	4. Equivocal but concerns in the average patient	5. Equivocal	6. Equivocal but probably OK in the average patient	7. Appropriate – some general exceptions	8. Appropriate – occasional exceptions	9. Appropriate – no exceptions
Appropriateness	☐	☐	☐	☐	☐	☐	☐	☐	☐

- An **'acute'** prescription refers to a prescription issued on a one-off basis for conditions that are often short-lived.
- The '**persistent**' prescribing refers to multiple prescriptions lasting three months or more.
- Scores 1 to 3: **Inappropriate** (i.e. no benefit, possible harms).
- Scores 4 to 6: **Uncertainty** (i.e. when harms and benefits are judged as approximately equal, or when the best available evidence does not support a judgement either way).
- Scores 7 to 9: **Appropriate** (i.e. benefits were judged to outweigh harms).

Appropriateness rating of revised scenario

Persistent prescription of opioid analgesics with a gabapentinoid, i.e. gabapentin or pregabalin.

Please rate the appropriateness of this scenario regarding the safety of opioid prescribing for average patients with chronic non-cancer pain in the general practice setting.

Please don't select more than 1 answer(s) per row.

Please select at least 1 answer(s).

	1. Inappropriate – no exceptions	2. Inappropriate – occasional exceptions	3. Inappropriate – some general exceptions	4. Equivocal but concerns in the average patient	5. Equivocal	6. Equivocal but probably OK in the average patient	7. Appropriate – some general exceptions	8. Appropriate – occasional exceptions	9. Appropriate – no exceptions
Appropriateness	☐	☐	☐	☐	☐	☐	☐	☐	☐

- An **'acute'** prescription refers to a prescription issued on a one-off basis for conditions that are often short-lived.
- The '**persistent**' prescribing refers to multiple prescriptions lasting three months or more.
- Scores 1 to 3: **Inappropriate** (i.e. no benefit, possible harms).
- Scores 4 to 6: **Uncertainty** (i.e. when harms and benefits are judged as approximately equal, or when the best available evidence does not support a judgement either way).
- Scores 7 to 9: **Appropriate** (i.e. benefits were judged to outweigh harms).

Feasibility rating of revised scenario

Persistent prescription of opioid analgesics with a gabapentinoid, i.e. gabapentin or pregabalin.

Please rate the feasibility of implementing this scenario regarding the safety of opioid prescribing for average patients with chronic non-cancer pain in the general practice setting.

⊞ More info

Please don't select more than 1 answer(s) per row.

Please select at least 1 answer(s).

	1. Unfeasible – no exceptions	2. Unfeasible – occasional exceptions	3. Unfeasible – some general exceptions	4. Equivocal but concerns in the average patient	5. Equivocal	6. Equivocal but probably OK in the average patient	7. Feasible – some general exceptions	8. Feasible – occasional exceptions	9. Feasible – no exceptions
Feasibility	☐	☐	☐	☐	☐	☐	☐	☐	☐

- Scores 1 to 3: **Unfeasible** (i.e. limited resource and capacity, high risk of failure).
- Scores 4 to 6: **Uncertainty** (i.e. resource and capacity are judged approximately equal to challenges).
- Scores 7 to 9: **Feasible** (i.e. resource and capacity were judged to outweigh the risk of failure).

Do you have any comment on this scenario as an indicator for safe opioid prescribing?

Page 17: Scenario 12. Acute or persistent prescription of opioid analgesics to a patient with galactose intolerance, lactase deficiency or glucose-galactose malabsorption.

Summary of Round 1 survey

Appropriateness rating

Category	Inappropriate			Equivocal			Appropriate		
Scale	1	2	3	4	5	6	7	8	9
Result	*1*	*2*	*2*	*1*	*10*	*2*	*1*	*5*	*0*

Overall panel median: 5.0, agreement on equivocality (* indicate your rating in the first round of Delphi sruvey).

Synopsis of comments

Concerns

- The severity of symptom (diarrhoea or bloating)
- Type of opioid formulations (preparation) and comedications (lactose quantity in excipients).

Exception

Acute use.

Mitigation

Supply anti-mobility drugs; ensure patient’s awareness of the risk.

Feasibility

This diagnosis is rare in the primary care setting. One participant expressed never seen a patient documented with the condition in the past 30 years.

Appropriateness rating

Acute or persistent prescription of opioid analgesics to a patient with galactose intolerance, lactase deficiency or glucose-galactose malabsorption.

Please rate the appropriateness of this scenario regarding the safety of opioid prescribing for average patients with chronic non-cancer pain in the general practice setting.

Please don't select more than 1 answer(s) per row.

Please select at least 1 answer(s).

	1. Inappropriate – no exceptions	2. Inappropriate – occasional exceptions	3. Inappropriate – some general exceptions	4. Equivocal but concerns in the average patient	5. Equivocal	6. Equivocal but probably OK in the average patient	7. Appropriate – some general exceptions	8. Appropriate – occasional exceptions	9. Appropriate – no exceptions

Appropriateness	☐	☐	☐	☐	☐	☐	☐	☐	☐

- An **'acute'** prescription refers to a prescription issued on a one-off basis for conditions that are often short-lived.
- The **'persistent'** prescribing refers to multiple prescriptions lasting three months or more.
- Scores 1 to 3: **Inappropriate** (i.e. no benefit, possible harms).
- Scores 4 to 6: **Uncertainty** (i.e. when harms and benefits are judged as approximately equal, or when the best available evidence does not support a judgement either way).
- Scores 7 to 9: **Appropriate** (i.e. benefits were judged to outweigh harms).

Appropriateness rating of revised scenario

Prescription of opioid analgesics to a patient with galactose intolerance, lactase deficiency or glucose-galactose malabsorption.

Please rate the appropriateness of this scenario regarding the safety of opioid prescribing for average patients with chronic non-cancer pain in the general practice setting.

Please don't select more than 1 answer(s) per row.

Please select at least 1 answer(s).

	1. Inappropriate – no exceptions	2. Inappropriate – occasional exceptions	3. Inappropriate – some general exceptions	4. Equivocal but concerns in the average patient	5. Equivocal	6. Equivocal but probably OK in the average patient	7. Appropriate – some general exceptions	8. Appropriate – occasional exceptions	9. Appropriate – no exceptions
Appropriateness	☐	☐	☐	☐	☐	☐	☐	☐	☐

- An **'acute'** prescription refers to a prescription issued on a one-off basis for conditions that are often short-lived.
- The **'persistent'** prescribing refers to multiple prescriptions lasting three months or more.
- Scores 1 to 3: **Inappropriate** (i.e. no benefit, possible harms).
- Scores 4 to 6: **Uncertainty** (i.e. when harms and benefits are judged as approximately equal, or when the best available evidence does not support a judgement either way).
- Scores 7 to 9: **Appropriate** (i.e. benefits were judged to outweigh harms).

Feasibility rating of revised scenario

Prescription of opioid analgesics to a patient with galactose intolerance, lactase deficiency or glucose-galactose malabsorption.

Please rate the feasibility of implementing this scenario regarding the safety of opioid prescribing for average patients with chronic non-cancer pain in the general practice setting.

More info

Please don't select more than 1 answer(s) per row.

Please select at least 1 answer(s).

	1. Unfeasible – no exceptions	2. Unfeasible – occasional exceptions	3. Unfeasible – some general exceptions	4. Equivocal but concerns in the average patient	5. Equivocal	6. Equivocal but probably OK in the average patient	7. Feasible – some general exceptions	8. Feasible – occasional exceptions	9. Feasible – no exceptions

Feasibility	☐	☐	☐	☐	☐	☐	☐	☐	☐

- Scores 1 to 3: **Unfeasible** (i.e. limited resource and capacity, high risk of failure).
- Scores 4 to 6: **Uncertainty** (i.e. resource and capacity are judged approximately equal to challenges).
- Scores 7 to 9: **Feasible** (i.e. resource and capacity were judged to outweigh the risk of failure).

Do you have any comment on this scenario as an indicator for safe opioid prescribing?

Page 18: Scenario 13. Persistent prescription of opioid analgesics to a patient with constipation and without a concurrently prescribed laxative.

Summary of Round 1 survey

Appropriateness rating

Category	Inappropriate			Equivocal			Appropriate		
Scale	1	2	3	4	5	6	7	8	9
Result	*7*	*8*	*4*	*1*	*2*	*1*	*1*	*0*	*0*

Overall panel median: 2.0, agreement on inappropriateness (* indicate your rating in the first round of Delphi sruvey).

Synopsis of comments

Concerns

- Patients taking persistent opioids should be prescribed with laxatives.
- Indication for prescribing opioids; opioid-induced constipation.
- Considering alternative medicines, e.g. peripherally acting mu-opioid receptor antagonist.

Exception

No specific comment.

Mitigation

- If the constipation is not resolved, stop the opioids to resolve constipation.
- Ensure patient's awareness of side effects (including constipation) of opioids.

Feasibility

- The accuracy of this indicator is unclear. It may identify patients with a Read code for the past constipation event but is currently prescribed with laxative PRN.
- Patients may not fill the prescription and take laxative regularly, or patients may buy over-the-counter laxatives.

Appropriateness rating

Persistent prescription of opioid analgesics to a patient with constipation and without a concurrently prescribed laxative.

Please rate the appropriateness of this scenario regarding the safety of opioid prescribing for average patients with chronic non-cancer pain in the general practice setting.

Please don't select more than 1 answer(s) per row.

Please select at least 1 answer(s).

	1. Inappropriate – no exceptions	2. Inappropriate – occasional exceptions	3. Inappropriate – some general exceptions	4. Equivocal but concerns in the average patient	5. Equivocal	6. Equivocal but probably OK in the average patient	7. Appropriate – some general exceptions	8. Appropriate – occasional exceptions	9. Appropriate – no exceptions
Appropriateness	☐	☐	☐	☐	☐	☐	☐	☐	☐

- The **'persistent'** prescribing refers to multiple prescriptions lasting three months or more.
- Scores 1 to 3: **Inappropriate** (i.e. no benefit, possible harms).
- Scores 4 to 6: **Uncertainty** (i.e. when harms and benefits are judged as approximately equal, or when the best available evidence does not support a judgement either way).
- Scores 7 to 9: **Appropriate** (i.e. benefits were judged to outweigh harms).

Feasibility rating

Persistent prescription of opioid analgesics to a patient with constipation and without a concurrently prescribed laxative.

Please rate the feasibility of implementing this scenario regarding the safety of opioid prescribing for average patients with chronic non-cancer pain in the general practice setting.

More info

Please don't select more than 1 answer(s) per row.

Please select at least 1 answer(s).

	1. Unfeasible – no exceptions	2. Unfeasible – occasional exceptions	3. Unfeasible – some general exceptions	4. Equivocal but concerns in the average patient	5. Equivocal	6. Equivocal but probably OK in the average patient	7. Feasible – some general exceptions	8. Feasible – occasional exceptions	9. Feasible – no exceptions
Feasibility	☐	☐	☐	☐	☐	☐	☐	☐	☐

- Scores 1 to 3: **Unfeasible** (i.e. limited resource and capacity, high risk of failure).
- Scores 4 to 6: **Uncertainty** (i.e. resource and capacity are judged approximately equal to challenges).
- Scores 7 to 9: **Feasible** (i.e. resource and capacity were judged to outweigh the risk of failure).

Do you have any comment on this scenario as an indicator for safe opioid prescribing?

Page 19: Scenario 14. Persistent prescription of opioid analgesics for greater than or equal to 6 months without a concurrently prescribed laxative.

Summary of Round 1 survey

Appropriateness rating

Category	Inappropriate			Equivocal			Appropriate		
Scale	1	2	3	4	5	6	7	8	9
Result	*4*	*9*	*5*	*0*	*3*	*2*	*1*	*0*	*0*

Overall panel median: 2.0, agreement on inappropriateness (* indicate your rating in the first round of Delphi sruvey).

Synopsis of comments

Concerns

- Laxatives must be prescribed unless with other contraindicating factors. The timing of treatment is irrelevant as constipation occur very fast (from day 1).
- Identify the issue at six months is too late as dependence and side effects may have become problematic.
- Consider the dose and type of opioids (morphine, oxycodone, buprenorphine) prescribed.
- Whether constipation occurred; whether patients used over-the-counter laxatives.

Exception

Patients do not suffer from constipation.

Mitigation

- Regular review and stop or reduce opioids as soon as possible.
- Ensure that patients are aware of the risk of constipation and optimal compliance with laxatives.

Feasibility

This is a common scenario, and the indicator will identify too many patients. Many patients stick to persistent opioids despite suffering from constipation. Patients may also purchase over-the-counter laxatives.

Appropriateness rating

Persistent prescription of opioid analgesics for greater than or equal to 6 months without a concurrently prescribed laxative. Please rate the appropriateness of this scenario regarding the safety of opioid prescribing for average patients with chronic non-cancer pain in the general practice setting.

Please don't select more than 1 answer(s) per row.

Please select at least 1 answer(s).

	1. Inappropriate – no exceptions	2. Inappropriate – occasional exceptions	3. Inappropriate – some general exceptions	4. Equivocal but concerns in the average patient	5. Equivocal	6. Equivocal but probably OK in the average patient	7. Appropriate – some general exceptions	8. Appropriate – occasional exceptions	9. Appropriate – no exceptions
Appropriateness	☐	☐	☐	☐	☐	☐	☐	☐	☐

- The **'persistent'** prescribing refers to multiple prescriptions lasting three months or more.
- Scores 1 to 3: **Inappropriate** (i.e. no benefit, possible harms).
- Scores 4 to 6: **Uncertainty** (i.e. when harms and benefits are judged as approximately equal, or when the best available evidence does not support a judgement either way).
- Scores 7 to 9: **Appropriate** (i.e. benefits were judged to outweigh harms).

Appropriateness rating of revised scenario

Persistent prescription of opioid analgesics without a concurrently prescribed laxative.

Please rate the appropriateness of this scenario regarding the safety of opioid prescribing for average patients with chronic non-cancer pain in the general practice setting.

Please don't select more than 1 answer(s) per row.

Please select at least 1 answer(s).

	1. Inappropriate – no exceptions	2. Inappropriate – occasional exceptions	3. Inappropriate – some general exceptions	4. Equivocal but concerns in the average patient	5. Equivocal	6. Equivocal but probably OK in the average patient	7. Appropriate – some general exceptions	8. Appropriate – occasional exceptions	9. Appropriate – no exceptions
Appropriateness	☐	☐	☐	☐	☐	☐	☐	☐	☐

- The **'persistent'** prescribing refers to multiple prescriptions lasting three months or more.
- Scores 1 to 3: **Inappropriate** (i.e. no benefit, possible harms).
- Scores 4 to 6: **Uncertainty** (i.e. when harms and benefits are judged as approximately equal, or when the best available evidence does not support a judgement either way).
- Scores 7 to 9: **Appropriate** (i.e. benefits were judged to outweigh harms).

Feasibility rating of revised scenario

Persistent prescription of opioid analgesics without a concurrently prescribed laxative.

Please rate the feasibility of implementing this scenario regarding the safety of opioid prescribing for average patients with chronic non-cancer pain in the general practice setting.

⊞ More info

Please don't select more than 1 answer(s) per row.

Please select at least 1 answer(s).

	1. Unfeasible – no exceptions	2. Unfeasible – occasional exceptions	3. Unfeasible – some general exceptions	4. Equivocal but concerns in the average patient	5. Equivocal	6. Equivocal but probably OK in the average patient	7. Feasible – some general exceptions	8. Feasible – occasional exceptions	9. Feasible – no exceptions
Feasibility	☐	☐	☐	☐	☐	☐	☐	☐	☐

- Scores 1 to 3: **Unfeasible** (i.e. limited resource and capacity, high risk of failure).
- Scores 4 to 6: **Uncertainty** (i.e. resource and capacity are judged approximately equal to challenges).
- Scores 7 to 9: **Feasible** (i.e. resource and capacity were judged to outweigh the risk of failure).

Do you have any comment on this scenario as an indicator for safe opioid prescribing?

Page 20: Scenario 15. Prescription of codeine or morphine to a patient with severe renal impairment, i.e. the most recent eGFR less than 30 mL/min per 1.73 square meters.

Summary of Round 1 survey

Appropriateness rating

Category	Inappropriate			Equivocal			Appropriate		
**Scale**	**1**	**2**	**3**	**4**	**5**	**6**	**7**	**8**	**9**
**Result**	*6*	*6*	*1*	*5*	*2*	*1*	*2*	*0*	*1*

Overall panel median: 1.0, agreement on inappropriateness (* indicate your rating in the first round of Delphi sruvey).

Synopsis of comments

Concerns

- Indication, dose and formation of opioids.
- Type of opioids: avoiding morphine, but this indicator may increase prescribing of oxycodone and fentanyl.
- Patients' kidney function (assuming acute kidney injury).

Exception

Acute use of codeine or morphine at a low dose; low dose opioids with regular review and patents are stable; when there is no alternative.

Mitigation

Reducing dose; use CrCl for adjusting dose adjustment; prescribe other analgesics (e.g. alfentanil); regular monitoring eGFR; discuss with patients about the opioid prescription.

Feasibility

No comment.

Appropriateness rating

Prescription of codeine or morphine to a patient with severe renal impairment, i.e. the most recent eGFR<30 mL/min per 1.73 m^2. Please rate the appropriateness of this scenario regarding the safety of opioid prescribing for average patients with chronic non-cancer pain in the general practice setting.

Please don't select more than 1 answer(s) per row.

Please select at least 1 answer(s).

	1. Inappropriate – no exceptions	2. Inappropriate – occasional exceptions	3. Inappropriate – some general exceptions	4. Equivocal but concerns in the average patient	5. Equivocal	6. Equivocal but probably OK in the average patient	7. Appropriate – some general exceptions	8. Appropriate – occasional exceptions	9. Appropriate – no exceptions

Appropriateness	☐	☐	☐	☐	☐	☐	☐	☐	☐

- Scores 1 to 3: **Inappropriate** (i.e. no benefit, possible harms).
- Scores 4 to 6: **Uncertainty** (i.e. when harms and benefits are judged as approximately equal, or when the best available evidence does not support a judgement either way).
- Scores 7 to 9: **Appropriate** (i.e. benefits were judged to outweigh harms).

Feasibility rating

Prescription of codeine or morphine to a patient with severe renal impairment, i.e. the most recent eGFR<30 mL/min per 1.73 m^2.

Please rate the feasibility of implementing this scenario regarding the safety of opioid prescribing for average patients with chronic non-cancer pain in the general practice setting.

More info

Please don't select more than 1 answer(s) per row.

Please select at least 1 answer(s).

	1. Unfeasible – no exceptions	2. Unfeasible – occasional exceptions	3. Unfeasible – some general exceptions	4. Equivocal but concerns in the average patient	5. Equivocal	6. Equivocal but probably OK in the average patient	7. Feasible – some general exceptions	8. Feasible – occasional exceptions	9. Feasible – no exceptions
Feasibility	☐	☐	☐	☐	☐	☐	☐	☐	☐

- Scores 1 to 3: **Unfeasible** (i.e. limited resource and capacity, high risk of failure).
- Scores 4 to 6: **Uncertainty** (i.e. resource and capacity are judged approximately equal to challenges).
- Scores 7 to 9: **Feasible** (i.e. resource and capacity were judged to outweigh the risk of failure).

Do you have any comment on this scenario as an indicator for safe opioid prescribing?

Page 21: Scenario 16. Persistent prescription of one or more opioid analgesics at a dose above the equivalent of 120 mg of oral morphine per day.

Summary of Round 1 survey

Appropriateness rating

Category	Inappropriate			Equivocal			Appropriate		
Scale	1	2	3	4	5	6	7	8	9
Result	*7*	*12*	*4*	*0*	*0*	*0*	*0*	*1*	*0*

Overall panel median: 2.0, Agreement on inappropriateness (* indicate your rating in the first round of Delphi sruvey).

Synopsis of comments

Concerns

- Persistent use of opioids in such a dose has no benefit in pain management but increases the risk of dependence and long-term harm. Tolerance can be rapidly developed, long-term, high dose of morphine is inappropriate.
- Need to involve pain management team as this scenario is mostly associated with complex patients with other comorbidities with chronic pain.

Exception

Patients who are on reducing dose; achieve good functional improvement than at a reduced dose; under the advice of a pain specialist; patients with cancer or palliative care.

Mitigation

Involving the pain teams; offer other options of pain management to reduce the dose of opioids.

Feasibility

The calculation of total opioid load may identify:

(1) Patients are on the process of reducing dose from the previous higher dose.

(2) Patients are adding other opioids to avoid recurrent use of high dose opioids.

(3) Patients are on several opioids, but each prescribed with less than 12 mg oral morphine equivalent.

Appropriateness rating

Persistent prescription of one or more opioid analgesics at a dose above the equivalent of 120 mg of oral morphine per day.
Please rate the appropriateness of this scenario regarding the safety of opioid prescribing for average patients with chronic non-cancer pain in the general practice setting.

Please don't select more than 1 answer(s) per row.

Please select at least 1 answer(s).

	1. Inappropriate – no exceptions	2. Inappropriate – occasional exceptions	3. Inappropriate – some general exceptions	4. Equivocal but concerns in the average patient	5. Equivocal	6. Equivocal but probably OK in the average patient	7. Appropriate – some general exceptions	8. Appropriate – occasional exceptions	9. Appropriate – no exceptions
Appropriateness	☐	☐	☐	☐	☐	☐	☐	☐	☐

- Scores 1 to 3: **Inappropriate** (i.e. no benefit, possible harms).
- Scores 4 to 6: **Uncertainty** (i.e. when harms and benefits are judged as approximately equal, or when the best available evidence does not support a judgement either way).
- Scores 7 to 9: **Appropriate** (i.e. benefits were judged to outweigh harms).

Appropriateness rating of revised scenario

Persistent prescription of one or more opioid analgesics at a total morphine equivalent load above 120 mg per day. Please rate the appropriateness of this scenario regarding the safety of opioid prescribing for average patients with chronic non-cancer pain in the general practice setting.

Please don't select more than 1 answer(s) per row.

Please select at least 1 answer(s).

	1. Inappropriate – no exceptions	2. Inappropriate – occasional exceptions	3. Inappropriate – some general exceptions	4. Equivocal but concerns in the average patient	5. Equivocal	6. Equivocal but probably OK in the average patient	7. Appropriate – some general exceptions	8. Appropriate – occasional exceptions	9. Appropriate – no exceptions
Appropriateness	☐	☐	☐	☐	☐	☐	☐	☐	☐

- Scores 1 to 3: **Inappropriate** (i.e. no benefit, possible harms).
- Scores 4 to 6: **Uncertainty** (i.e. when harms and benefits are judged as approximately equal, or when the best available evidence does not support a judgement either way).
- Scores 7 to 9: **Appropriate** (i.e. benefits were judged to outweigh harms).

Feasibility rating of revised scenario

Persistent prescription of one or more opioid analgesics at a total morphine equivalent load above 120 mg per day. Please rate the feasibility of implementing this scenario regarding the safety of opioid prescribing for average patients with chronic non-cancer pain in the general practice setting.

More info

Please don't select more than 1 answer(s) per row.

Please select at least 1 answer(s).

	1. Unfeasible – no exceptions	2. Unfeasible – occasional exceptions	3. Unfeasible – some general exceptions	4. Equivocal but concerns in the average patient	5. Equivocal	6. Equivocal but probably OK in the average patient	7. Feasible – some general exceptions	8. Feasible – occasional exceptions	9. Feasible – no exceptions
Feasibility	☐	☐	☐	☐	☐	☐	☐	☐	☐

- Scores 1 to 3: **Unfeasible** (i.e. limited resource and capacity, high risk of failure).
- Scores 4 to 6: **Uncertainty** (i.e. resource and capacity are judged approximately equal to challenges).
- Scores 7 to 9: **Feasible** (i.e. resource and capacity were judged to outweigh the risk of failure).

Do you have any comment on this scenario as an indicator for safe opioid prescribing?

Page 22: Scenario 17. Acute or persistent prescription of opioid analgesics to a patient for more than three months following the patient’s discharge from hospital after surgery.

Summary of Round 1 survey

Appropriateness rating

Category	Inappropriate			Equivocal			Appropriate		
Scale	1	2	3	4	5	6	7	8	9
Result	*5*	*14*	*2*	*2*	*1*	*0*	*0*	*0*	*0*

Overall panel median: 2.0, agreement on inappropriateness (* indicate your rating in the first round of Delphi sruvey).

Synopsis of comments

Concerns

Type of surgery (rehabilitation time, tolerance of physiotherapy; post-surgical chronicity or complications); the reason for surgery (pre-existing conditions); and the indication of opioids (for surgical pain ongoing conditions). Three months may be too long as the tolerance and dependence may develop rapidly.

Exception

Acute use of opioids for complications in the recovery phase; for ongoing complications; problems are not improved or exacerbated; patients with cancer.

Mitigation

Give a full assessment of the condition and discuss the pain management plan with patients.

Feasibility

This indicator may identify patients with pre-existing conditions and on opioids before surgery. It will also identify the acute use of opioids for complications in the recovery phase, but it may be useful to identify the unintentionally chronic opioid use.

Appropriateness rating

Acute or persistent prescription of opioid analgesics to a patient for more than three months following the patient’s discharge from hospital after surgery. Please rate the appropriateness of this scenario regarding the safety of opioid prescribing for average patients with chronic non-cancer pain in the general practice setting.

Please don't select more than 1 answer(s) per row.

Please select at least 1 answer(s).

	1. Inappropriate – no exceptions	2. Inappropriate – occasional exceptions	3. Inappropriate – some general exceptions	4. Equivocal but concerns in the average patient	5. Equivocal	6. Equivocal but probably OK in the average patient	7. Appropriate – some general exceptions	8. Appropriate – occasional exceptions	9. Appropriate – no exceptions
Appropriateness	☐	☐	☐	☐	☐	☐	☐	☐	☐

- An **'acute'** prescription refers to a prescription issued on a one-off basis for conditions that are often short-lived.
- The **'persistent'** prescribing refers to multiple prescriptions lasting three months or more.
- Scores 1 to 3: **Inappropriate** (i.e. no benefit, possible harms).
- Scores 4 to 6: **Uncertainty** (i.e. when harms and benefits are judged as approximately equal, or when the best available evidence does not support a judgement either way).
- Scores 7 to 9: **Appropriate** (i.e. benefits were judged to outweigh harms).

Appropriateness rating of revised scenario

Persistent prescription of opioid analgesics following the patient's discharge from hospital after surgery. Please rate the appropriateness of this scenario regarding the safety of opioid prescribing for average patients with chronic non-cancer pain in the general practice setting.

Please don't select more than 1 answer(s) per row.

Please select at least 1 answer(s).

	1. Inappropriate – no exceptions	2. Inappropriate – occasional exceptions	3. Inappropriate – some general exceptions	4. Equivocal but concerns in the average patient	5. Equivocal	6. Equivocal but probably OK in the average patient	7. Appropriate – some general exceptions	8. Appropriate – occasional exceptions	9. Appropriate – no exceptions
Appropriateness	☐	☐	☐	☐	☐	☐	☐	☐	☐

- An **'acute'** prescription refers to a prescription issued on a one-off basis for conditions that are often short-lived.
- The **'persistent'** prescribing refers to multiple prescriptions lasting three months or more.
- Scores 1 to 3: **Inappropriate** (i.e. no benefit, possible harms).
- Scores 4 to 6: **Uncertainty** (i.e. when harms and benefits are judged as approximately equal, or when the best available evidence does not support a judgement either way).
- Scores 7 to 9: **Appropriate** (i.e. benefits were judged to outweigh harms).

Feasibility rating of revised scenario

Persistent prescription of opioid analgesics following the patient's discharge from hospital after surgery. Please rate the feasibility of implementing this scenario regarding the safety of opioid prescribing for average patients with chronic non-cancer pain in the general practice setting.

⊞ More info

Please don't select more than 1 answer(s) per row.

Please select at least 1 answer(s).

	1. Unfeasible – no exceptions	2. Unfeasible – occasional exceptions	3. Unfeasible – some general exceptions	4. Equivocal but concerns in the average patient	5. Equivocal	6. Equivocal but probably OK in the average patient	7. Feasible – some general exceptions	8. Feasible – occasional exceptions	9. Feasible – no exceptions
Feasibility	☐	☐	☐	☐	☐	☐	☐	☐	☐

- Scores 1 to 3: **Unfeasible** (i.e. limited resource and capacity, high risk of failure).
- Scores 4 to 6: **Uncertainty** (i.e. resource and capacity are judged approximately equal to challenges).
- Scores 7 to 9: **Feasible** (i.e. resource and capacity were judged to outweigh the risk of failure).

Do you have any comment on this scenario as an indicator for safe opioid prescribing?

Page 23: Scenario 18. Persistent prescription of opioid analgesics to a patient with at least moderate hepatic impairment.

Summary of Round 1 survey

Appropriateness rating

Category	Inappropriate			Equivocal			Appropriate		
Scale	1	2	3	4	5	6	7	8	9
Result	*0*	*9*	*4*	*4*	*3*	*3*	*1*	*0*	*0*

Overall panel median: 2.0, agreement on inappropriateness (* indicate your rating in the first round of Delphi sruvey).

Synopsis of comments

Concerns

- The nature and severity of hepatic impairment: biliary issues causing pain; need to know the liver function test and coagulation screening results. It is not a contraindication for moderate hepatic impairment.
- Avoided for patients with severe hepatic impairment, increase the risk of encephalopathy associated with constipation.
- Type and dose of opioids.

Exception

Acute use of opioids; patients with terminal conditions.

Mitigation

Opioid dose modification; avoid other medicines that may further impair hepatic metabolism.

Feasibility

It is challenging to define hepatic impairment.

Appropriateness rating

Persistent prescription of opioid analgesics to a patient with at least moderate hepatic impairment. Please rate the appropriateness of this scenario regarding the safety of opioid prescribing for average patients with chronic non-cancer pain in the general practice setting.

Please don't select more than 1 answer(s) per row.

Please select at least 1 answer(s).

	1. Inappropriate – no exceptions	2. Inappropriate – occasional exceptions	3. Inappropriate – some general exceptions	4. Equivocal but concerns in the average patient	5. Equivocal	6. Equivocal but probably OK in the average patient	7. Appropriate – some general exceptions	8. Appropriate – occasional exceptions	9. Appropriate – no exceptions

Appropriateness	☐	☐	☐	☐	☐	☐	☐	☐	☐

- The **'persistent'** prescribing refers to multiple prescriptions lasting three months or more.
- Scores 1 to 3: **Inappropriate** (i.e. no benefit, possible harms).
- Scores 4 to 6: **Uncertainty** (i.e. when harms and benefits are judged as approximately equal, or when the best available evidence does not support a judgement either way).
- Scores 7 to 9: **Appropriate** (i.e. benefits were judged to outweigh harms).

Feasibility rating

Persistent prescription of opioid analgesics to a patient with at least moderate hepatic impairment.

Please rate the feasibility of implementing this scenario regarding the safety of opioid prescribing for average patients with chronic non-cancer pain in the general practice setting.

⊞ More info

Please don't select more than 1 answer(s) per row.

Please select at least 1 answer(s).

	1. Unfeasible – no exceptions	2. Unfeasible – occasional exceptions	3. Unfeasible – some general exceptions	4. Equivocal but concerns in the average patient	5. Equivocal	6. Equivocal but probably OK in the average patient	7. Feasible – some general exceptions	8. Feasible – occasional exceptions	9. Feasible – no exceptions
Feasibility	☐	☐	☐	☐	☐	☐	☐	☐	☐

- Scores 1 to 3: **Unfeasible** (i.e. limited resource and capacity, high risk of failure).
- Scores 4 to 6: **Uncertainty** (i.e. resource and capacity are judged approximately equal to challenges).
- Scores 7 to 9: **Feasible** (i.e. resource and capacity were judged to outweigh the risk of failure).

Do you have any comment on this scenario as an indicator for safe opioid prescribing?

Page 24: Scenario 19. Persistent prescription of opioid analgesics to a patient aged over 65 years with a recent medical history of falling.

Summary of Round 1 survey

Appropriateness rating

Category	Inappropriate			Equivocal			Appropriate		
Scale	1	2	3	4	5	6	7	8	9
Result	*4*	*8*	*4*	*5*	*0*	*3*	*0*	*0*	*0*

Overall panel median: 2.0, agreement on inappropriateness (* indicate your rating in the first round of Delphi sruvey).

Synopsis of comments

Concerns

- Persistent use of opioids is inappropriate and should be avoided.
- Causes of falls; definition of 'recent' medical history.
- Reason for prescribing opioids (e.g. prescribing after falls); type and does of opioids.
- Other concomitant medication causing falls.

Exception

Acute use for severe pain or pain following fall; the end of life stage.

Mitigation

- Medication review to identify the risk of fall and avoid concomitant medication (e.g. stop loop diuretics).
- Patient education and supervision to reduce the risk of falling.

Feasibility

No comment.

Appropriateness rating

Persistent prescription of opioid analgesics to a patient aged over 65 years with a recent medical history of falling.

Please rate the appropriateness of this scenario regarding the safety of opioid prescribing for average patients with chronic non-cancer pain in the general practice setting.

Please don't select more than 1 answer(s) per row.

Please select at least 1 answer(s).

	1. Inappropriate – no exceptions	2. Inappropriate – occasional exceptions	3. Inappropriate – some general exceptions	4. Equivocal but concerns in the average patient	5. Equivocal	6. Equivocal but probably OK in the average patient	7. Appropriate – some general exceptions	8. Appropriate – occasional exceptions	9. Appropriate – no exceptions

Appropriateness	☐	☐	☐	☐	☐	☐	☐	☐	☐

- The **'persistent'** prescribing refers to multiple prescriptions lasting three months or more.
- the **'recent medical history'** is medical conditions recorded in the patients' electronic health records in the past 12 months.
- Scores 1 to 3: **Inappropriate** (i.e. no benefit, possible harms).
- Scores 4 to 6: **Uncertainty** (i.e. when harms and benefits are judged as approximately equal, or when the best available evidence does not support a judgement either way).
- Scores 7 to 9: **Appropriate** (i.e. benefits were judged to outweigh harms).

Appropriateness rating of revised scenario

Persistent prescription of opioid analgesics in a patient aged over 65 years with a medical history of falling. Please rate the appropriateness of this scenario regarding the safety of opioid prescribing for average patients with chronic non-cancer pain in the general practice setting.

Please don't select more than 1 answer(s) per row.

Please select at least 1 answer(s).

	1. Inappropriate – no exceptions	2. Inappropriate – occasional exceptions	3. Inappropriate – some general exceptions	4. Equivocal but concerns in the average patient	5. Equivocal	6. Equivocal but probably OK in the average patient	7. Appropriate – some general exceptions	8. Appropriate – occasional exceptions	9. Appropriate – no exceptions
Appropriateness	☐	☐	☐	☐	☐	☐	☐	☐	☐

- The **'persistent'** prescribing refers to multiple prescriptions lasting three months or more.
- the **'recent medical history'** is medical conditions recorded in the patients' electronic health records in the past 12 months.
- Scores 1 to 3: **Inappropriate** (i.e. no benefit, possible harms).
- Scores 4 to 6: **Uncertainty** (i.e. when harms and benefits are judged as approximately equal, or when the best available evidence does not support a judgement either way).
- Scores 7 to 9: **Appropriate** (i.e. benefits were judged to outweigh harms).

Feasibility rating of revised scenario

Persistent prescription of opioid analgesics in a patient aged over 65 years with a medical history of falling. Please rate the feasibility of implementing this scenario regarding the safety of opioid prescribing for average patients with chronic non-cancer pain in the general practice setting.

More info

Please don't select more than 1 answer(s) per row.

Please select at least 1 answer(s).

	1. Unfeasible – no exceptions	2. Unfeasible – occasional exceptions	3. Unfeasible – some general exceptions	4. Equivocal but concerns in the average patient	5. Equivocal	6. Equivocal but probably OK in the average patient	7. Feasible – some general exceptions	8. Feasible – occasional exceptions	9. Feasible – no exceptions

Feasibility	☐	☐	☐	☐	☐	☐	☐	☐	☐

- Scores 1 to 3: **Unfeasible** (i.e. limited resource and capacity, high risk of failure).
- Scores 4 to 6: **Uncertainty** (i.e. resource and capacity are judged approximately equal to challenges).
- Scores 7 to 9: **Feasible** (i.e. resource and capacity were judged to outweigh the risk of failure).

Do you have any comment on this scenario as an indicator for safe opioid prescribing?

Page 25: Scenario 20. Persistent prescription of tramadol, buprenorphine or oxycodone to a patient with a medical history of ventricular tachycardia.

Summary of Round 1 survey

Appropriateness rating

Category	Inappropriate			Equivocal			Appropriate		
**Scale**	**1**	**2**	**3**	**4**	**5**	**6**	**7**	**8**	**9**
**Result**	*0*	*14*	*3*	*1*	*3*	*2*	*1*	*0*	*0*

Overall panel median: 2.0, agreement on inappropriateness (* indicate your rating in the first round of Delphi sruvey).

Synopsis of comments

Concerns

- Reason for opioids.
- Whether patents had ablation or was on Sotalol or was trained to do self-carotid sinus massage?
- Methadone should be included as it is associated with the highest incidence of supraventricular tachycardia or ventricular tachycardia.

Exception

No comment.

Mitigation

No comment.

Feasibility

Ventricular tachycardia is poorly documented, and hence it is hard to identify patients with ventricular tachycardia or palpitation from the primary care records.

Appropriateness rating

Persistent prescription of tramadol, buprenorphine or oxycodone to a patient with a medical history of ventricular tachycardia.

Please rate the appropriateness of this scenario regarding the safety of opioid prescribing for average patients with chronic non-cancer pain in the general practice setting.

Please don't select more than 1 answer(s) per row.

Please select at least 1 answer(s).

	1. Inappropriate – no exceptions	2. Inappropriate – occasional exceptions	3. Inappropriate – some general exceptions	4. Equivocal but concerns in the average patient	5. Equivocal	6. Equivocal but probably OK in the average patient	7. Appropriate – some general exceptions	8. Appropriate – occasional exceptions	9. Appropriate – no exceptions

Appropriateness	☐	☐	☐	☐	☐	☐	☐	☐	☐

- The **'persistent'** prescribing refers to multiple prescriptions lasting three months or more.
- The **'medical history'** refers to any conditions which are documented in the patient's electronic health records.
- Scores 1 to 3: **Inappropriate** (i.e. no benefit, possible harms).
- Scores 4 to 6: **Uncertainty** (i.e. when harms and benefits are judged as approximately equal, or when the best available evidence does not support a judgement either way).
- Scores 7 to 9: **Appropriate** (i.e. benefits were judged to outweigh harms).

Feasibility rating

Persistent prescription of tramadol, buprenorphine or oxycodone to a patient with a medical history of ventricular tachycardia.

Please rate the feasibility of implementing this scenario regarding the safety of opioid prescribing for average patients with chronic non-cancer pain in the general practice setting.

More info

Please don't select more than 1 answer(s) per row.

Please select at least 1 answer(s).

	1. Unfeasible – no exceptions	2. Unfeasible – occasional exceptions	3. Unfeasible – some general exceptions	4. Equivocal but concerns in the average patient	5. Equivocal	6. Equivocal but probably OK in the average patient	7. Feasible – some general exceptions	8. Feasible – occasional exceptions	9. Feasible – no exceptions
Feasibility	☐	☐	☐	☐	☐	☐	☐	☐	☐

- Scores 1 to 3: **Unfeasible** (i.e. limited resource and capacity, high risk of failure).
- Scores 4 to 6: **Uncertainty** (i.e. resource and capacity are judged approximately equal to challenges).
- Scores 7 to 9: **Feasible** (i.e. resource and capacity were judged to outweigh the risk of failure).

Do you have any comment on this scenario as an indicator for safe opioid prescribing?

Page 26: Additional comments on Opioid Safety Prescribing Indicators

Synopsis of additional comments in the first round of Delphi survey

In the first round of Delphi survey, the panel's recommendations for future opioid safety prescribing indicators focused on the following risk factors. Besides, it was also suggested that future indicator development should focus on clinical indicators rather than risk factors or contradictions. You can download this **synposis page** (by clicking on the link).

Medicine-related risk factors

- Persistent prescription of opioids regardless of indication or comorbidities.
- Potency of opioids
- The dose of opioids: differentiate low and high dose, set a more restrict level of dose (e.g. 100 mg oral morphine equivalent per day), differentiate patients on a stable dose, reducing the dose and increasing dose,
- Concurrent opioid use: tramadol and fentanyl patch; buprenorphine and oxycodone modified-release; a combination of multiple low-potency opioids
- Opioid preparation: differentiate sustained release and intermediate dose, avoid modified-release opioids
- Combination of benzodiazepine, gabapentinoid and opioids.

Patient-related risk factors

- Whether patients are opioid naïve or already on opioids?
- History of suicide and self-harm (risk of overdose) or alcohol abuse.
- Elderly patients, women wjo are breastfeeding, males with co-prescribed medication for erectile dysfunction.
- Patients with certain professions (e.g. lorry or taxi driver).

Risk factors of aberrant opioid use behaviours

- Repeated prescription of opioids without consultation, patients with no record of indication for opioids, patients getting opioids from multiple sources.
- Request for an early prescription (ordering script before the due date), various dose missing (dropped bottles, loss of medicines, misplace medicine, the pharmacy did not supply correct amount)
- Vulnerable adults living with a family who might be with a risk of opioid diversion, patient’s home environment, partner or carer

Outcome-related indicators

- Pain is controlled.
- Reduction of non-elective hospital admission due to non-cancer pain or the adverse events of opioids prescribed for non-cancer pain
- Reduction of deaths in patients with chronic non-cancer pain (not at the end of life stage) who were prescribed with one or more opioids.
- Frequency of medication review done by a healthcare professional in the community.

Do you have any additional comment regarding the development of potential prescribing safety indicators for optimising opioid prescribing in general practices?

More info

○ No ○ Yes

If yes, please list below.

Page 27: Final page

Thank you very much for completing the second round of the Delphi survey. Your participation has been beneficial to this project. The insightful comments from participants are invaluable clinical practice knowledge for us to develop further work in medication safety. We look forward to sharing the results with you soon. If you have any question, please do not hesitate to contact the research team. Thank you.

Dr Li-Chia Chen (Email: li-chia.chen@manchester.ac.uk)
