# Supplementary material for: Development of prescribing indicators related to opioid-related harm in patients with chronic pain in primary care—a modified e-Delphi study
Source: BMC Med. 2024 Jan 2;22:5. doi: 10.1186/s12916-023-03213-x (PMC10763174; doi:10.1186/s12916-023-03213-x)
Supplement: Supplementary file 7 — Additional file 7. Summary of first-round results for one individual panellist. [file 12916_2023_3213_MOESM7_ESM.pdf]

Delphi Survey Round 1 Results

* indicates your rating in the first round.

No.	Scenario	Inappropriate			Equivocal			Appropriate			First round		Second round
		1	2	3	4	5	6	7	8	9	panel median	agreement	revision
1	Persistent prescription of opioid analgesics to a patient with a medical history of alcohol addiction, abuse or dependence.	3*	9	6	5	0	0	0	1	0	2.5	Inappropriateness	Unchange
2	Acute or persistent prescription of opioid analgesics to a woman during	0	13*	5	3	3	0	0	0	0	2	Inappropriateness	Revised
3	Persistent prescription of opioid analgesics to a patient with	0	3	4	2	4*	4	2	3	2	5	Equivocality	Revised
4	Persistent prescription of opioid analgesics to a patient with paralytic	8*	10	5	1	0	0	0	0	0	2	Inappropriateness	Unchange
5	Persistent prescription of opioid analgesics to a patient with dementia	2	10	4*	2	3	2	1	0	0	2.5	Inappropriateness	Unchange
6	Persistent prescription of opioid analgesics to a patient with chronic obstructive pulmonary disease or asthma	0	6	4	3	2*	5	2	2	0	4	Equivocality	Revised
7	Co-prescription of opioid analgesics with carbamazepine, phenytoin or phenobarbital to a patient with epilepsy	0	4	5	5	3	3*	3	1	0	4	Equivocality	Revised
8	Persistent prescription of opioid analgesics to a patient with myasthenia	2	4	5	6*	4	0	1	2	0	4	Equivocality	Unchange
9	Acute or persistent co-prescription of opioid analgesics with antidepressants, i.e. monoamine oxidase inhibitors, selective serotonin reuptake inhibitors, or serotonin and norepinephrine	0	4	3	5	5	3*	1	3	0	4.5	Equivocality	Revised
10	Acute or persistent co-prescription of opioid analgesics with a	0	8	4*	4	4	2	1	1	0	3.5	Inappropriateness	Revised
11	Acute or persistent co-prescription of opioid analgesics with a gabapentinoid, i.e. gabapentin or pregabalin	0	7	4*	1	6	3	2	1	0	4.5	Equivocality	Revised
12	Acute or persistent prescription of opioid analgesics to a patient with galactose intolerance, lactase deficiency or glucose-galactose	1	2	2	1	10*	2	1	5	0	5	Equivocality	Revised
13	Persistent prescription of opioid analgesics to a patient with constipation and without a concurrently prescribed laxative	7	8	4*	1	2	1	1	0	0	2	Inappropriateness	Unchange
14	Persistent prescription of opioid analgesics for greater than or equal to 6 months without a concurrently prescribed laxative	4	9	5*	0	3	2	1	0	0	2	Inappropriateness	Revised
15	Prescription of codeine or morphine to a patient with severe renal impairment, i.e. the most recent eGFR<30 mL/min per 1.73 m2	6*	6	1	5	2	1	2	0	1	1	Inappropriateness	Unchange
16	Persistent prescription of one or more opioid analgesics at a dose above the equivalent of 120 mg of oral morphine per day	7	12	4*	0	0	0	0	1	0	2	Inappropriateness	Revised
17	Acute or persistent prescription of opioid analgesics to a patient for more than three months following the patient's discharge from hospital	5	14*	2	2	1	0	0	0	0	2	Inappropriateness	Revised
18	Persistent prescription of opioid analgesics to a patient with at least moderate hepatic impairment	0	9	4*	4	3	3	1	0	0	2	Inappropriateness	Unchange
19	Persistent prescription of opioid analgesics to a patient aged over 65 years with a recent medical history of falling	4	8	4*	5	0	3	0	0	0	2	Inappropriateness	Revised
20	Persistent prescription of tramadol, buprenorphine or oxycodone to a patient with a medical history of ventricular tachycardia	0	14	3	1*	3	2	1	0	0	2	Inappropriateness	Unchange
