# Supplementary material for: Development of prescribing indicators related to opioid-related harm in patients with chronic pain in primary care—a modified e-Delphi study
Source: BMC Med. 2024 Jan 2;22:5. doi: 10.1186/s12916-023-03213-x (PMC10763174; doi:10.1186/s12916-023-03213-x)
Supplement: Supplementary file 9 — Additional file 9. Evidence sources for the opioid safety prescribing indicators. [file 12916_2023_3213_MOESM9_ESM.docx]

Additional file 9. Evidence sources for the opioid safety prescribing indicators

| **No.** | **Indicator** | **Original study** | **Systematic review** | **Guidelines** | **Other** |
| --- | --- | --- | --- | --- | --- |
| 1 | Persistent prescription of opioid analgesics to a patient with a medical history of alcohol addiction, abuse or dependence (1-4) [N=4] | CS [N=1]  RCT [N=1] | - | B [N=1]  A [N=1] | - |
| 2 | Acute or persistent prescription of opioid analgesics to a woman during pregnancy (5-13) [N=9] | CCS [N=2] | - | A [N=1]  B [N=6] | - |
| 3 | Persistent prescription of opioid analgesics to a patient with hypothyroidism (14-16) [N=3] | CSS [N=1] | + [N=1] | - | E [N=1] |
| 4 | Persistent prescription of opioid analgesics to a patient with paralytic ileus (17, 18) [N=2] | CCS [N=1] | - | - | E [N=1] |
| 5 | Persistent prescription of opioid analgesics to a patient with dementia (19, 20) [N=2] | - | + [N=1] | - | E [N=1] |
| 6 | Persistent prescription of opioid analgesics to a patient with chronic obstructive pulmonary disease or asthma (21) [N=1] | CS [N=1] | - | - | - |
| 7 | Co-prescription of opioid analgesics with carbamazepine, phenytoin or phenobarbital to a patient with epilepsy (22-26) [N=5] | - | - | B [N=2]  C [N=1] | E [N=2] |
| 8 | Persistent prescription of opioid analgesics to a patient with myasthenia gravis (27-29) [N=3] | - | - | B [N=1] | E [N=2] |
| 9 | Acute or persistent co-prescription of opioid analgesics with antidepressants, i.e. monoamine oxidase inhibitors, selective serotonin reuptake inhibitors, or serotonin and norepinephrine reuptake inhibitors (4, 23, 24, 30-33) [N=7] | - | + [N=1] | (A [N=1]  B [N=3] | E [N=1]  F [N=1] |
| 10 | Acute or persistent co-prescription of opioid analgesics with benzodiazepine (34-38) [N=5] | CS [N=2]  CCS [N=1] | + [N=1] | B [N=1] | - |
| 11 | Acute or persistent co-prescription of opioid analgesics with a gabapentinoid, i.e. gabapentin or pregabalin (39-42) [N=4] | CCS [N=2] | - | A [N=1] | E [N=2]  F [N=1] |
| 12 | Acute or persistent prescription of opioid analgesics to a patient with galactose intolerance, lactase deficiency or glucose-galactose malabsorption (43, 44) [N=2] | - | - | - | E [N=1]  F [N=1] |
| 13 | Persistent prescription of opioid analgesics to a patient with constipation and without a concurrently prescribed laxative (45, 46) [N=2] | - | - | - | E [N=2] |
| 14 | Persistent prescription of opioid analgesics for greater than or equal to 6 months without a concurrently prescribed laxative (45, 46) [N=2] | - | - | - | E [N=2] |
| 15 | Prescription of codeine or morphine to a patient with severe renal impairment, i.e. the most recent eGFR <30mL/min per 1.73 m2 (47, 48) [N=2] | CS [N=1] | - | - | E [N=1] |
| 16 | Persistent prescription of one or more opioid analgesics at a dose above the equivalent of 120mg of oral morphine per day (4, 16, 33, 49) [N=4] | - | ++ [N=2] | A [N=1]  B [N=1] | - |
| 17 | Acute or persistent prescription of opioid analgesics to a patient for more than three months following the patient's discharge from the hospital after surgery (50, 51) [N=2] | CS [N=2] | - | - | - |
| 18 | Persistent prescription of opioid analgesics to a patient with at least moderate hepatic impairment (52-54) [N=3] | - | - | - | E [N=3] |
| 19 | Persistent prescription of opioid analgesics to a patient aged over 65 years with a recent medication history of falling (36, 37, 55) [N=3] | CS [N=1]  CCS [N=1] | - | - | E [N=1] |
| 20 | Persistent prescription of tramadol, buprenorphine, and oxycodone to a patient with a medical history of ventricular tachycardia (56, 57) [N=2] | CS [N=1] | + | - | - |

(Note) CCS: case-control study; CS: a cohort study; RCT: randomised control trial; +: yes; -: No; A: local and national guidelines; B: recommendations from professional bodies; C: NICE guidelines; D: BNF; E: editorials; F: case study; G: professional opinions; H: webpages.

**References**

1. Midanik L. T., Tam T. W., Weisner C. Concurrent and simultaneous drug and alcohol use: results of the 2000 National Alcohol Survey. Drug Alcohol Depend. 2007;90(1):72-80.

2. Thomas S. Dangers of Mixing Alcohol and Opiates, including Hydrocodone, Oxycodone and Morphine: American Addiction Centres; 2019 [Available from: <https://americanaddictioncenters.org/alcoholism-treatment/mixing-opiates>.

3. Gianoulakis C., de Waele J. P., Thavundayil J. Implication of the endogenous opioid system in excessive ethanol consumption. Alcohol. 1996;13(1):19-23.

4. Wigan Borough CCG. (GMMMG) Opioid Prescribing for Chronic Pain: Resource Pack. 2018.

5. UK Medicines Information(UKMi) Q&A. Can opioids be used for pain relief during pregnancy (by OK Medicines Information (UKMi) pharmacists for NHS healthcare professionals) 2017 [Available from: <https://www.sps.nhs.uk/articles/can-opioids-be-used-for-pain-relief-during-pregnancy/>.

6. Yazdy M. M., Mitchell A. A., Tinker S. C., Parker S. E., Werler M. M. Periconceptional use of opioids and the risk of neural tube defects. Obstet Gynecol. 2013;122(4):838-44.

7. Broussard C. S., Rasmussen S. A., Reefhuis J., Friedman J. M., Jann M. W., Riehle-Colarusso T., et al. Maternal treatment with opioid analgesics and risk for birth defects. American Journal of Obstetrics and Gynecology. 2011;204(4):314.e1-11.

8. Summary of Product Characteristics. Codeine phosphate tablets 30mg: Actavis UK Ltd; 2017 [Available from: <https://www.medicines.org.uk/emc/product/2375/smpc>.

9. UK Teratology Information Service. Morphine in Pregnancy. Version 2 2016 [Available from: <http://www.toxbase.org/>

10. UK Teratology Information Service. Codeine or dihydrocodeine in pregnancy 2015 [Available from: <http://www.toxbase.org/>

11. UK Teratology Information Service. Tramadol in pregnancy. Version 2 2016 [Available from: <http://www.toxbase.org/>

12. UK Teratology Information Service. Oxycodone in Pregnancy 2016 [updated May 2016. Available from: <http://www.toxbase.org/>

13. UK Teratology Information Service. Fentanyl in pregnancy. Version 1 2012 [updated April 2012. Available from: <http://www.toxbase.org/>

14. Daniell H. W. Opioid endocrinopathy in women consuming prescribed sustained-action opioids for control of nonmalignant pain. Journal of Pain. 2008;9(1):28-36.

15. Seyfried O., Hester J. Opioids and endocrine dysfunction. British Journal of Pain. 2012;6(1):17-24.

16. Baldini A., Von Korff M., Lin E. H. A Review of Potential Adverse Effects of Long-Term Opioid Therapy: A Practitioner's Guide. The Primary Care Companion for CNS Disorders. 2012;14(3).

17. Kurz A., Sessler D. I. Opioid-induced bowel dysfunction: pathophysiology and potential new therapies. Drugs. 2003;63(7):649-71.

18. Goettsch W. G., Sukel M. P., van der Peet D. L., van Riemsdijk M. M., Herings R. M. In-hospital use of opioids increases rate of coded postoperative paralytic ileus. Pharmacoepidemiol Drug Safety. 2007;16(6):668-74.

19. Erdal A., Ballard C., Vahia I. V., Husebo B. S. Analgesic treatments in people with dementia - how safe are they? A systematic review. Expert Opinion on Drug Safety. 2019;30:1-12.

20. University of Exeter Dementia Research. Commonly prescribed medications linked to rise in harmful side effects in dementia 2018 [Available from: <http://www.exeter.ac.uk/dementia/news/#vpYT2U57FhajCbut.99>.

21. Vozoris N. T., Wang X., Fischer H. D., Bell C. M., O'Donnell D. E., Austin P. C., et al. Incident opioid drug use and adverse respiratory outcomes among older adults with COPD. European Respiratory Journal. 2016;48(3):683-93.

22. National Institute of Health and Care Excellence (NICE). Tramadol Interactions [Available from: <https://bnf.nice.org.uk/interaction/tramadol.html>

23. al. VNe. Patient considerations in the use of tapentadol for moderate to severe pain. Drug, Healthcare and Patient Safety. 2013;5:151-9.

24. Medicines and Healthcare products Regulatory Agency (MHRA). Tapentadol (Palexia): risk of seizures and reports of serotonin syndrome when co-administered with other medicines. Drug Safety Update. 2019;12(6):1.

25. Savage R. Serious Reactions with Tramadol: Seizures and Serotonin Syndrome. Prescriber Update 2007;28(1):11-3.

26. Carter E. L., Adapa R. M. Adult epilepsy and anaesthesia. Continuing Education in Anaesthesia Critical Care & Pain. 2015;15(3):111-7.

27. Haroutiunian S., Lecht S., Zur A. A., Hoffman A., Davidson E. The challenge of pain management in patients with myasthenia gravis. Journal of Pain and Palliative Care Pharmacotherapy. 2009;23(3):242-60.

28. Boneva N., Brenner T., Argov Z. Gabapentin may be hazardous in myasthenia gravis. Muscle Nerve. 2000;23(8):1204-8.

29. Pascuzzi R. M. Medications and Myasthenia Gravis (A Reference for Health Care Professionals) In: America MGFo, editor. 2000.

30. Nelson E. M., Philbrick A. M. Avoiding serotonin syndrome: the nature of the interaction between tramadol and selective serotonin reuptake inhibitors. Annals of Pharmacotherapy. 2012;46(12):1712-6.

31. Rang S.T., Field J., Irving C. Serotonin toxicity caused by an interaction between fentanyl and paroxetine. Canadian Journal of Anaesthesia. 2008;55(8):521-5.

32. All Wales Medicines Strategy Group. National Prescribing Indicators 2018-2019. 2018.

33. Faculty of Pain Medicine of the Royal College of Anaesthetists. Opioids Aware 2015 [Available from: <http://www.fpm.ac.uk/faculty-of-pain-medicine/opioidsaware>.

34. Jones J. D., Mogali S., Comer S. D. Polydrug abuse: A review of opioid and benzodiazepine combination use. Drug & Alcohol Dependence. 2012 125(1-2):8-18.

35. European Monitoring Centre for Drugs and Drug Addiction (EMCDDA). The misuse of benzodiazepines among high-risk opioid users in Europe 2018 [Available from: <http://www.emcdda.europa.eu/publications/pods/benzodiazepines_en>.

36. Machado-Dugue M. E., Castano-Montoya J. P., Medina-Morales D. A., Castro-Rodriguez A., Machado-Alba J. E. Association between the use of benzodiazepines and opioids with the risk of falls and hip fractures in older adults. International Psychogeriatrics 2018;30(7)::941-6.

37. Miller M., Sturmer T., Azrael D., Levin R., Solomon D. H. Opioid analgesics and the risk of fractures in older adults with arthritis. Journal of the American Geriatric Society. 2011;59(3):430-8.

38. Hirschtritt M. E., Delucchi K. L., Olfson M. Outpatient, combined use of opioid and benzodiazepine medications in the United States, 1993–2014. Preventative Medicine Reports. 2018;9:49-54

39. Goodman C. W., Brett A. S. Gabapentin and Pregabalin for Pain — Is Increased Prescribing a Cause for Concern? . The New England Journal of Medicine 2017(377):411-4.

40. RCP London. Medicines and Falls in Hospital: Guidance Sheet [Available from: <https://www.rcplondon.ac.uk/file/933/download?token=drzlaAJ2>

41. Gomes T., Greaves S., Brink W., Antoniou T., Mamdani M. M., Paterson J. M., et al. Pregabalin and the risk for opioid-related Death: A nested case–control study. Annals of Internal Medicine. 2018;169(10):732-4.

42. Gomes T., Juurlink D. N., Antoniou T., Mamdani M. M., Paterson J. M., Brink W. Gabapentin, opioids, and the risk of opioid-related death: A population-based nested case–control study. PLoS Medicine. 2017;14(10).

43. Bril S., Shoham Y., Marcus J. The 'mystery' of opioid-induced diarrhea. Pain Research and Managment. 2011;16(3):197-9.

44. Mill D., Dawson J., Johnson J. L. Managing acute pain in patients who report lactose intolerance: the safety of an old excipient re-examined. Therapeutic Advances in Drug Safety. 2018;9(5):227-35.

45. Gupta OSaM. Opioid Induced Constipation. Treasure Island (FL): StatPearls [Internet]. 2019.

46. Chokhavatia S., John E. S., Bridgeman M. B., Dixit D. Constipation in Elderly Patients with Noncancer Pain: Focus on Opioid-Induced Constipation. Drugs Aging. 2016;33(8):557-74.

47. Novick T. Lifetime Opiate and Cocaine Use and Chronic Kidney Disease. American Journal of Nephrology. 2016;44(6):447-53.

48. Dean M. Opioids in renal failure and dialysis patients. Journal of Pain Symptom Management. 2004;28(5):497-504.

49. Chou R., Turner J. A., Devine E. B., Hansen R. N., Sullivan S. D., Blazina I., et al. The Effectiveness and Risks of Long-Term Opioid Therapy for Chronic Pain: A Systematic Review for a National Institutes of Health Pathways to Prevention Workshop. Annals of Internal Medicine. 2015;162(4):276-86.

50. Stark N. Prevalence and predictors of persistent post-surgical opioid use: a prospective observational cohort study. Anaesthesia and Intensive Care. 2017;45(6):700-6.

51. Bateman B. T., Franklin J. M., Bykov K., Avorn J., Shrank W. H., Brennan T. A. Persistent opioid use following cesarean delivery: patterns and predictors among opioid-naïve women. American Journal of Obstetrics and Gynecology. 2016;215(3):353.e1-.e18.

52. Tegeder I., Lötsch J., Geisslinger G. Pharmacokinetics of opioids in liver disease. Clinical Pharmacokinetics. 1999;37(1):17-40.

53. Bosilkovska M., Walder B., Besson M., Daali Y., Desmeules J. Analgesics in patients with hepatic impairment: pharmacology and clinical implications. Drugs. 2012;72(12):1645-69.

54. Rakoski M., Goyal P., Spencer-Safier M., Weissman J., Mohr G., Volk M. Pain management in patients with cirrhosis. Clinical Liver Disease: A Multimedia Review Journal. 2018;11(6):135-40.

55. Thompson H. J., McCormick W. C., Kagan S. H. Traumatic brain injury in older adults: epidemiology, outcomes, and future implications. Journal of the American Geriatrics Society. 2006;54(10):1590-5.

56. Kao D. P., Haigney M. C., Mehler P. S., Krantz M J. Arrhythmia associated with buprenorphine and methadone reported to the Food and Drug Administration. Addiction 2015;110(9):1468-75.

57. Behzadi M., Joukar S., Beik A. Opioids and Cardiac Arrhythmia: A Literature Review. Medical Principles and Practice. 2018;27(5):401-14.
