# Supplementary material for: Development of prescribing indicators related to opioid-related harm in patients with chronic pain in primary care—a modified e-Delphi study
Source: BMC Med. 2024 Jan 2;22:5. doi: 10.1186/s12916-023-03213-x (PMC10763174; doi:10.1186/s12916-023-03213-x)
Supplement: Supplementary file 10 — Additional file 10. Synopsis of the participants' comments on each scenario from the first round of a Delphi survey. [file 12916_2023_3213_MOESM10_ESM.docx]

Additional file 10. Synopsis of the participants' comments on each scenario from the first round of a Delphi survey

| **No.** | **Indicator** | **Concerns** | **Exception** | **Mitigation** | **Feasibility** |
| --- | --- | --- | --- | --- | --- |
| 1 | Persistent prescription of opioid analgesics to a patient with a medical history of alcohol addiction, abuse, or dependence  (Agreement on Inappropriateness) | - Persistent opioid use (should generally be avoided regardless of alcohol misuse) - Concomitant use with other drugs/substances other than alcohol - Escalating dose due to abuse - Other substance abuse - Other risk factors such as hepatic, cardiac, and respiratory function | - Patients who are at their end-of-life stage whose pain and alcohol abuse are controlled or when pain is prioritised over other conditions | - Patients starting on opioids should be reviewed after three months. - Consider alternative drugs before prescribing opioids. - If persistent opioid treatment is needed, offer short-term prescriptions with more frequent monitoring, and ensure the patient knows the risks. | - Addiction, abuse, and dependence are not wholly recorded in the GP system. - It is difficult to differentiate between ongoing or past addiction, abuse, and dependence |
| 2 | Acute or persistent prescription of opioid analgesics to a woman during pregnancy  (Agreement on Inappropriateness) | - Persistent use in the late stage of pregnancy - Indications for the prescription (e.g., tapper from previous analgesic use) | - Short term use (e.g., co-codamol 30/500mg) - Tapping from prior use - Treatment for symphysis pubis dysfunction | - Conduct risk and benefit assessment; if the prescription is needed, discuss risk, expectations, and treatment duration with the patient. | - Pregnancy Read code may stay on patients' records after they are pregnant. It is difficult to differentiate the ongoing or past pregnancy |
| 3 | Persistent prescription of opioid analgesics to a patient with hypothyroidism  (Agreement on Equivocality) | - Lack of clarity- specify indicator for treated/untreated hypothyroidism. - Lack of information regarding the condition of hypothyroidism- untreated hypothyroidism (may include respiratory depression, low mood) - Persistent prescription (should generally be avoided) - Indication of opioids - Type of opioids prescribed | - Is hypothyroidism well managed? - It may depend on the type of opioids. - This patient group would not be viewed differently compared to any other patients as all persistent opioid Rx will be screened in primary care. - Acute prescription - Well-controlled pain and good thyroid function test result | - Review all patients with persistent opioid prescriptions. - Regular monitoring of relevant clinical indicators (e.g., thyroid function test) | No comment. |
| 4 | Persistent prescription of opioid analgesics to a patient with paralytic ileus  (Agreement on Inappropriateness) | - Severity of pain - Cause of paralytic ileus (opioid-induced, post-surgery or cancer) - Reasons for prescribing opioids - Risk of exacerbating the paralytic ileus (due to opioid-induced constipation) | - Non-oral preparations such as epidural prescribed in a speciality or surgical setting - Patients who are at the end-of-life stage | - Discuss risks of long-term use of opioids with the patient during diagnosis, initiation and titration of treatment - Try to reduce the amount prescribed | - Paralytic ileus is normally diagnosed in a hospital setting and generally not recorded in the GP system. - Even if a read code of paralytic ileus is detected, it is likely to be a past event |
| 5 | Persistent prescription of opioid analgesics to a patient with dementia  (Agreement on Inappropriateness) | - Type of dementia (including manifestation of symptoms and comorbidities) - The severity of pain and its impact on a patient with dementia (may cause distress, delirium, exacerbations, change of behaviour) - Type of opioids - Patient's compliance with the drug regimen - Impact of opioid-related side effects on patients with dementia (falls, depression of CNS, cognitive impairment) | - Intolerable pain even with regular analgesics - Very distressed patients - Patients at end-of-life stage | - Review patients on opioids with dementia regularly - Avoid dose escalation. - Consider tapering off the dose when opioids are deemed unnecessary. - Ensure carers are aware of risks and side effects. - Provide patient-specific caring guidance. | No comment. |
| 6 | Persistent prescription of opioid analgesics to a patient with chronic obstructive pulmonary disease or asthma  (Agreement on equivocality) | - Persistent opioid use (should generally be avoided) - Opioid-related respiratory depression - The severity of respiratory disease (frequency of exacerbations, oxygen supply) - The severity of pain and its impact on quality of life | - Asthma - A low dose of opioids (e.g., morphine) for dyspnoea in COPD - Patients at the end stage of COPD or life | - Review patients with complex conditions and multiple comorbidities regularly. - Pulse Oximetry test monitoring. - Reduce the dose of opioids. - Supply naloxone to patients or carers as a precaution | No comment. |
| 7 | Co-prescription of opioid analgesics with carbamazepine, phenytoin or phenobarbital to a patient with epilepsy  (Agreement on Equivocality) | - Reason for prescribing opioids (whether the pain is controlled & epilepsy is stable) - Type and dose of opioids prescribed. - Opioid-related CNS effects - Drug-drug interactions (affecting the metabolism and potentially causing more drowsiness with phenobarbital) | - Acute use with no intention of continuing long-term (e.g., post-discharge stage) - Seizure caused by injury or trauma. - When up-titration is required | - Cautious and regular monitoring - Reduce the dose of opioids | No comment. |
| 8 | Persistent prescription of opioid analgesics to a patient with myasthenia gravis  (Agreement on Inappropriateness) | - Persistent opioid use (should generally be avoided) - The severity of myasthenia gravis (e.g., affecting intercostal or ocular muscles) and whether myasthenia gravis is stable. - Increased risk of CNS depression, drowsiness, and fall | - In a case where it is not a contraindication, depending on patients | No comment. | No comment. |
| 9 | Acute or persistent co-prescription of opioid analgesics with antidepressants, i.e., monoamine oxidase inhibitors, selective serotonin reuptake inhibitors, or serotonin and norepinephrine reuptake inhibitors  (Agreement on Equivocality) | - Persistent opioid use (should generally be avoided) - Reason for prescribing considering comorbidities. - Stability of patient's condition - Type of opioids (avoid MR formulations, tramadol related serotonin syndrome) - Type of antidepressants (avoid MAOIs as it causes severe adverse effects) - Risk of (accidental or intentional) overdose by introducing depressive patients an agent with a potential of misuse - Drug-drug interaction (tramadol) and increased sedation effects | - Acute use (e.g., surgery or trauma) - When up-titration is required | - Provide holistic approaches to pain management. - Ensure patient understand the expectations of treatments. - Review patients regularly, monitor usage and check for interactions. - Reduce dose to avoid CNS depression | - This indicator would identify too many patients |
| 10 | Acute or persistent co-prescription of opioid analgesics with benzodiazepine  (Agreement on Inappropriateness) | - Persistent opioid use (should generally be avoided) - Common drug combination in elderly despite the risks - Increased risk of overdose and side effects (e.g., CNS depression, respiratory depression, falls) - Balancing risks and benefits in patients considering the indication of opioids, patient's comorbidities, and substance use (e.g., alcohol) | - Acute use (e.g., post-surgery) - When the patient is at end-of-life stage - The low dose of a benzodiazepine - When up-titration is required | - Ensure patient is aware of the risks. - Supply naloxone to patients and carers and educate them on how to use it in the case of an emergency | - This indicator would identify too many patients as this is a common scenario |
| 11 | Acute or persistent co-prescription of opioid analgesics with a gabapentinoid, i.e., gabapentin or pregabalin  (Agreement on Inappropriateness) | - Persistent opioid use (should generally be avoided) - Nature of pain (e.g., neuropathic pain) and indications of both opioids and gabapentinoids - Increased risks of CNS depression, confusion, ataxia, falls and opioid-induced ventilation impairment. - Dose and regimen of opioids and gabapentinoids - Dose escalation increases sedative effects | - Acute use (e.g., injury or pain exacerbation) - Patients suffering from different types of pain. - Cross-tapering (gabapentinoids for neuropathic pain + opioids at low dose or PRN) | - Ensure nature of pain is identified. - Review patients after 3 months of opioid use - Involve pain management team. - Avoid co-prescribing opioids and gabapentinoids whenever possible. - Supply naloxone and educate patients on how to use it in the case of an emergency | - This indicator would identify too many patients as this is a common scenario. - Gabapentinoids are often not initiated with opioids at the same time. - Co-codamol is commonly prescribed before gabapentinoids |
| 12 | Acute or persistent prescription of opioid analgesics to a patient with galactose intolerance, lactase deficiency or glucose-galactose malabsorption  (Agreement on Equivocality) | - The severity of symptoms (e.g., diarrhoea, bloating) - Type of opioid formulations and comedications (e.g., lactose quantity in excipients) | - Acute use | - Supply anti-mobility drugs. - Ensure patient is aware of the risks | - This diagnosis is rare in primary setting; a participant expressed that they've never encountered a patient with the documented condition in the past 30 years |
| 13 | Persistent prescription of opioid analgesics to a patient with constipation and without a concurrently prescribed laxative  (Agreement on Inappropriateness) | - Patients on opioids for the long-term should be prescribed laxatives. - Opioid-induced constipation - Reason for prescribing opioids | No comment. | - If constipation is not resolved, stop the opioids to resolve constipation. - Ensure the patient is aware of the side effects associated with opioids (including constipation) | - The accuracy of this indicator is unclear. - The indicator may identify patients with a Read code for past constipation event that has been resolved/ now prescribed with laxative PRN |
| 14 | Persistent prescription of opioid analgesics for greater than or equal to 6 months without a concurrently prescribed laxative  (Agreement on Inappropriateness) | - Laxatives must be prescribed regardless of the length of treatment unless contraindicated. - Identifying the issue in 6 months would be too late as dependence and side effects may have arisen. - Dose and type of opioid prescribed - Whether the patient has used OTC laxatives to treat constipation | - When patients do not suffer from constipation | - Review patient regularly - Stop or reduce opioids as soon as possible | - The indicator would identify too many patients as this is a common scenario. - Many patients stick to persistent opioids despite suffering from constipation. - Patients may purchase OTC laxatives. |
| 15 | Prescription of codeine or morphine to a patient with severe renal impairment, i.e., the most recent eGFR <30mL/min per 1.73 m^2^  (Agreement on Inappropriateness) | - Indication, dose, and formulation of opioids - Type of opioids (to avoid morphine); however, concerned with increased prescription of oxycodone and fentanyl because of the abovementioned recommendation. - Patient's kidney function | - Acute use of codeine or morphine at a low dose - Use of low dose opioids with regular reviews on patients with stable conditions - When there is no alternative | - Reducing dose - Use CrCl for dose adjustments. - Regular kidney function monitoring (eGFR) - Ensure patients are aware of the risks. - Prescribe other analgesics (e.g., alfentanil) | No comment. |
| 16 | Persistent prescription of one or more opioid analgesics at a dose above the equivalent to 120mg of oral morphine per day.  (Agreement on Inappropriateness) | - Persistent use of opioids in such a dose has no benefit in pain management and increases the risk of dependence and long-term harm (tolerance can be rapidly developed) - The pain management team needs to be involved as this scenario is mostly associated with patients with complex conditions and other comorbidities with chronic pain | - Patients who are on a reduced dose - The good functional improvement achieved. - Under the advice of a pain specialist - Patients with cancer or in palliative care | - Involve pain specialists. - Offer other options of pain management to reduce the dose of opioids whenever possible | - The calculation of the total opioid load may identify.  1. Patients in the process of dose reduction 2. Patients who are using additional opioids to avoid recurrent use of high dose opioids. 3. Patients who are on several opioids, but each prescribed with less than 12mg oral morphine equivalent |
| 17 | Acute or persistent prescription of opioid analgesics to a patient for more than three months following the patient's discharge from hospital after surgery.  (Agreement on Inappropriateness) | - Type of surgery (rehabilitation time, tolerance of physiotherapy, post-surgical chronicity, complications, etc) - Reason for surgery - Indication for persistent opioids as tolerance and dependence may develop rapidly in 3 months | - Acute use of opioids for complications in the recovery phase - Indicated for ongoing complications. - When the condition has not improved or has exacerbated - Patients with cancer | - Carry out a full assessment on the condition and discuss pain management plans with patients to ensure they are fully aware of the risks | - This indicator may identify patients with pre-existing conditions and are opioids even before surgery. - It may also identify the acute use of opioids for complications in the recovery phase. - This indicator may be useful to identify unintentional chronic opioid uses |
| 18 | Persistent prescription of opioid analgesics to a patient with at least moderate hepatic impairment.  (Agreement on Inappropriateness) | - The nature and severity of hepatic impairment - Increased risk of encephalopathy associated with constipation (avoided in patients with severe hepatic impairment) | - Acute use of opioids - Patients with terminal conditions | - Opioid dose modification - Avoid other medications that may further impair the hepatic metabolism | - It is challenging to define hepatic impairment |
| 19 | Persistent prescription of opioid analgesics to a patient aged over 65 years with a recent medication history of falling.  (Agreement on Inappropriateness) | - Persistent opioid use (should generally be avoided) - Cause of falls & recent medical history - Reason for prescribing opioids (was it due to falls or did opioid induce the falls?) - Type and dose of opioids - Other concomitant medication causing falls | - Acute use for severe pain - Pain following falls. - Patients at the end-of-life stage | - Medication review to identify the risk of fall. - Avoid concomitant medication (e.g., stop loop diuretics) - Patient education and supervision to reduce risk of falling | No comment. |
| 20 | Persistent prescription of tramadol, buprenorphine, oxycodone to a patient with a medical history of ventricular tachycardia  (Agreement on Inappropriateness) | - Reasons for prescribing opioids - Whether patients have had ablation or was on Sotalol or was trained to do self-carotid sinus massage - Methadone should be included as it is associated with the highest incidence of supraventricular tachycardia or ventricular tachycardia | No comment. | No comment. | - It is difficult to identify patients with ventricular tachycardia or palpitations as it is poorly documented in the primary care records |

(Note) CNS; central nervous system; COPD; chronic obstructive pulmonary disease; CrCl; creatinine clearance; eGFR; estimated glomerular filtration rate; GP; general practice; MAOIs; monoamine oxidase inhibitors ; MR; modified release; OTC; over-the-counter; PRN; as needed (pro re nata)
