# Supplementary material for: Development of prescribing indicators related to opioid-related harm in patients with chronic pain in primary care—a modified e-Delphi study
Source: BMC Med. 2024 Jan 2;22:5. doi: 10.1186/s12916-023-03213-x (PMC10763174; doi:10.1186/s12916-023-03213-x)
Supplement: Supplementary file 11 — Additional file 11. Consensus on the feasibility of modified prescribing scenarios rated in the second round of the e-Delphi survey. [file 12916_2023_3213_MOESM11_ESM.docx]

**Additional file 11.** **Consensus on the feasibility of modified prescribing scenarios rated in the second round of the e-Delphi survey**

| **No.** | **Indicator** | **Median** (30th, 70th) |  | | | **Agreement** (Index) |
| --- | --- | --- | --- | --- | --- | --- |
|  |  |  | 1-3 | 4-6 | 7-9 |  |
| 1 | Persistent prescription of opioid analgesics to a patient with a medical history of alcohol addiction, abuse or dependence | 3 (2, 5) | 11 | 3 | 5 | A/IF  (0.395) |
| 2 | Persistent prescription of opioid analgesics during pregnancy | 5 (3, 8) | 7 | 3 | 9 | Neutral  (0.820) |
| 3 | Persistent prescription of opioid analgesics for a patient with untreated hypothyroidism | 5 (4, 7) | 5 | 6 | 8 | Neutral  (0.658) |
| 4 | Persistent prescription of opioid analgesics to a patient with paralytic ileus | 2 (2, 6) | 12 | 2 | 5 | A/IF  (0.292) |
| 5 | Persistent prescription of opioid analgesics to a patient with dementia | 6 (4, 8) | 5 | 5 | 9 | Neutral  (0.876) |
| 6 | Persistent prescription of opioid analgesics to a patient with severe chronic obstructive pulmonary disease or asthma | 6 (4, 7) | 3 | 8 | 8 | Neutral  (0.789) |
| 7 | Persistent prescription of tramadol or tapentadol with carbamazepine, phenytoin or phenobarbital in a patient with epilepsy | 8 (5, 8) | 4 | 4 | 11 | Disagreement  (1.053) |
| 8 | Persistent prescription of opioid analgesics to a patient with myasthenia gravis | 7 (4, 7) | 4 | 4 | 11 | A/F  (0.921) |
| 9 | Persistent prescription of tramadol, tapentadol, fentanyl, dextromethorphan, and pethidine with selective serotonin reuptake inhibitors or serotonin-norepinephrine reuptake inhibitors | 7 (4, 8) | 4 | 3 | 12 | Disagreement  (1.022) |
|  | Prescription of tramadol, tapentadol, fentanyl, dextromethorphan, and pethidine with a monoamine oxidase inhibitor (MAOI), including the 14 days following the withdrawal of an MAOI | 7 (5, 8) | 5 | 4 | 10 | A/F  (0.921) |
| 10 | Persistent prescription of opioid analgesics with a benzodiazepine | 7 (3, 7) | 7 | 2 | 10 | Disagreement  (1.022) |
| 11 | Persistent prescription of opioid analgesics with a gabapentinoid, i.e. gabapentin or pregabalin | 6 (4, 7) | 4 | 7 | 8 | Neutral  (0.789) |
| 12 | Prescription of opioid analgesics to a patient with galactose intolerance, lactase deficiency or glucose-galactose malabsorption | 5 (3, 6) | 6 | 9 | 4 | Neutral  (0.658) |
| 13 | Persistent prescription of opioid analgesics to a patient with constipation and without a concurrently prescribed laxative | 6 (2, 8) | 6 | 5 | 8 | Disagreement  1.121 |
| 14 | Persistent prescription of opioid analgesics without a concurrently prescribed laxative | 7 (5, 7) | 4 | 5 | 10 | A/F  (0.838) |
| 15 | Prescription of codeine or morphine to a patient with severe renal impairment, i.e. the most recent eGFR<30 mL/min per 1.73 m2 | 7 (2, 7) | 7 | 1 | 11 | Disagreement  (1.148) |
| 16 | Persistent prescription of one or more opioid analgesics at a total morphine equivalent load above 120 mg per day | 7 (3, 8) | 6 | 3 | 10 | Disagreement  (1.148) |
| 17 | Persistent prescription of opioid analgesics following the patient’s discharge from the hospital after surgery | 7 (5, 8) | 5 | 3 | 11 | A/F  (0.921) |
| 18 | Persistent prescription of opioid analgesics to a patient with at least moderate hepatic impairment | 5 (3, 7) | 6 | 6 | 7 | Neutral  (0.730) |
| 19 | Persistent prescription of opioid analgesics in a patient aged over 65 years with a medical history of falling | 7 (3, 7) | 7 | 2 | 10 | Disagreement  (1.022) |
| 20 | Persistent prescription of tramadol, buprenorphine or oxycodone to a patient with a medical history of ventricular tachycardia | 5 (3, 5) | 6 | 8 | 5 | Neutral  (0.599) |

Please rate the feasibility of implementing this scenario regarding the safety of opioid prescribing for average patients with chronic non-cancer pain in the general practice setting.
